# The effects of public health and social measures (PHSM) implemented during the COVID‐19 pandemic: An overview of systematic reviews

**DOI:** 10.1002/cesm.12055

**Published:** 2024-04-29

**Authors:** Racha Fadlallah, Fadi El‐Jardali, Lama Bou Karroum, Nour Kalach, Reem Hoteit, Andrew Aoun, Lara Al‐Hakim, Francisca Verdugo‐Paiva, Gabriel Rada, Atle Fretheim, Simon Lewin, Ramona Ludolph, Elie A. Akl

**Affiliations:** ^1^ Center for Systematic Reviews for Health Policy and Systems Research American University of Beirut Beirut Lebanon; ^2^ Department of Health Management and Policy American University of Beirut Beirut Lebanon; ^3^ Knowledge to Policy (K2P) Center American University of Beirut Beirut Lebanon; ^4^ Clinical Research Institute American University of Beirut Beirut Lebanon; ^5^ Epistemonikos Foundation Santiago Chile; ^6^ Orofacial Pain and TMD Program, Facultad de Odontología Universidad Andrés Bello Santiago Chile; ^7^ Centre for Epidemic Interventions Research Norwegian Institute of Public Health Oslo Norway; ^8^ Faculty of Health Sciences Oslo Metropolitan University Oslo Norway; ^9^ Department of Health Sciences Ålesund Norwegian University of Science and Technology (NTNU) Trondheim Norway; ^10^ Health Systems Research Unit South African Medical Research Council Cape Town South Africa; ^11^ Department of Epidemic and Pandemic Preparedness and Prevention, WHO Health Emergencies Programme World Health Organization Geneva Switzerland; ^12^ Department of Internal Medicine American University of Beirut Beirut Lebanon

**Keywords:** COVID‐19 pandemic, effectiveness, nonpharmaceutical interventions, overview of systematic reviews, public health and social measures, reviews, unintended consequences

## Abstract

**Introduction:**

To systematically review the effectiveness and unintended health and socioeconomic consequences of public health and social measures (PHSM) aimed at reducing the scale and risk of transmission of coronavirus disease 2019 (COVID‐19).

**Methods:**

This review followed guidance about overviews of reviews in the *Cochrane handbook for systematic reviews of interventions* and used the Epistemonikos database's COVID‐19 Living Overview of Evidence repository as a primary search source. Methodological quality was evaluated using the Measurement Tool to Assess Systematic Reviews (AMSTAR 2) checklist.

**Results:**

A total of 94 reviews were included, of which eight (9%) had “moderate” to “high” confidence ratings on the AMSTAR 2. Of 16 reviews (17%) reporting applying the GRADE framework, none found high certainty evidence for any of our outcomes of interest. Across the 94 reviews, the most frequently examined PHSM were personal protection (*n* = 18, 19%). Within multicomponent interventions, so‐called “lockdown” was the most frequently examined component (*n* = 39, 41%). The most frequently reported outcome category was non‐COVID‐19‐related health outcomes (*n* = 58, 62%). Only five (5%) reviews reported on socioeconomic outcomes. Findings from the eight reviews with moderate or high confidence ratings on AMSTAR 2 are narratively summarized. There is low‐certainty evidence that multicomponent interventions may reduce the transmission of COVID‐19 in different settings. For active surveillance and response measures, low‐certainty evidence suggests that routine testing of residents and staff in long‐term care facilities may reduce the number of infections, hospitalizations, and deaths among residents. We found very low‐certainty evidence about the effectiveness of personal protection measures, travel‐related control measures, and environmental measures. Unintended consequences were rarely examined by those eight reviews.

**Conclusion:**

We found predominantly low‐ to very low‐certainty evidence regarding the effectiveness and unintended consequences of PHSM in controlling the risk and scale of COVID‐19 transmission. There is a need to improve the conduct and reporting of systematic reviews.

## 1 INTRODUCTION

Coronavirus disease 2019 (COVID‐19) spread globally and was declared a public health emergency of international concern by the World Health Organization (WHO) on January 30, 2020 [1]. The number of confirmed COVID‐19 cases and deaths escalated rapidly, causing massive economic strain and increasing health care and public health expenditures [2, 3].

Responses to the pandemic included a wide range of public health and social measures (PHSM) [4]. PHSM range from personal protection measures, such as handwashing and mask‐wearing, to social measures, such as physical distancing and modifying school and business operations, as well as implementing international travel and trade restrictions [4, 5]. In 2021, WHO launched a multiyear initiative to strengthen the global evidence base about PHSM and promote equitable and contextualized PHSM implementation [6].

To inform decisions made by the public, the health workforce and policy‐makers, there is a need to synthesize the large volume of published research on PHSM for COVID‐19 [7]. Given the substantial number of systematic reviews on this topic, an overview of these reviews is a useful approach for bringing together this evidence [8, 9, 10].

Existing overviews of systematic reviews on interventions for COVID‐19 vary in their scope. Some are limited in the outcomes they consider, for example focusing only on symptoms and signs of COVID‐19 in children and adolescents [11] or on transmission‐related outcomes [12, 13]. Others have a broad focus, for example considering systematic reviews on any question related to COVID‐19 from the onset of the pandemic up to a certain date, regardless of scope or focus [10, 14]. To our knowledge, none of the overviews of systematic reviews published to date comprehensively address the unintended health and socioeconomic consequences of PHSM [15].

The objectives of this overview of systematic reviews are to assess:

- the effectiveness of single and combined PHSM in reducing the risk and scale of transmission of COVID‐19;
- the unintended health and socioeconomic consequences of these single and combined PHSM.

## 2 METHODS

This overview was based on guidance from the *Cochrane handbook for systematic reviews of interventions*, specifically the chapter about overviews of reviews [9]. A detailed protocol is published on medRxiv (https://doi.org/10.1101/2023.11.21.23298387). The PRIOR checklist (preferred reporting items for overviews of reviews) was followed in reporting the review [16].

### 2.1 Eligibility criteria

We included systematic reviews focusing on the effectiveness and/or unintended health and socioeconomic consequences of PHSM implemented during the COVID‐19 pandemic.

Given the lack of a consensus definition of a systematic review, Cochrane's guidance for overviews of reviews recommends using pre‐established criteria to make decisions around inclusion. Thus, for this review, we defined a systematic review as a publication that meets the following criteria [17]: its main purpose is to synthesize primary studies; it defines at least one explicit eligibility criterion for studies included in the review; and it reports searching at least one electronic database.

A draft conceptual framework developed by the University of Munich and WHO [18] was used to define and group PHSM. PHSM refer to a broad array of nonpharmaceutical interventions implemented by individuals, communities, and governments to reduce the risk and scale of transmission of epidemic‐ and pandemic‐prone infectious diseases. Pharmaceutical interventions, such as the administration of therapeutics and vaccines, are not included in this review, in line with the WHO PHSM conceptual framework. PHSMs were grouped into seven categories:

- active surveillance measures (e.g., screening, testing, or contact tracing irrespective of setting);
- response measures (e.g., quarantine and isolation);
- service measures (e.g., at schools or businesses, including closures, staggered arrival, break, and departure times, or the use of immunity or vaccination certificates);
- social interactions (e.g., physical distancing, restrictions on gatherings);
- physical environment measures (e.g., the use of physical barriers, air purifiers or ventilation, or through the cleaning of objects and surfaces);
- personal protection measures (e.g., handwashing, respiratory etiquette, face masks, face shields, or gloves); and
- movement restrictions (e.g., border closure or restrictions on domestic mobility).

We included implementation of both single and multicomponent interventions.

Reviews were excluded that did not assess the risk of bias in their included studies; also excluded were publications or preprints on servers such as medRxiv, bioRxiv, Litcovid, and the Social Science Research Network (known as SSRN). These exclusions were intended to help restrict the pool to higher‐quality reviews.

Detailed eligibility criteria are outlined in Table 1.

**Table 1:** Overview of eligibility criteria.

Variable	Inclusion criteria	Exclusion criteria
Study design	Systematic reviews of primary studies of any design (experimental, observational, or modeling studies) providing information about the effectiveness and/or unintended health and socioeconomic consequences of PHSM implemented in response to the COVID‐19 pandemic. For duplicate publications, the most recent or complete version was selected. Given the lack of a consensus definition of a systematic review, Cochrane's guidance for overviews of reviews recommends using pre‐established criteria to make decisions around inclusion. Thus, for this review, we defined a systematic review as a publication that meets the following criteria [17]: – its main purpose is to synthesize primary studies; – it defines at least one explicit eligibility criterion for studies included in the review; – it reports searching at least one electronic database. Rapid reviews were included if they met the predefined inclusion criteria noted above.	Conference abstracts, meeting proceedings, editorials, commentaries, and primary studies were excluded as it was considered unlikely that these would provide useful data related to the overview objectives. Reviews that did not assess a risk of bias in their included studies in some way were excluded to restrict the pool to reviews that were more likely to be well conducted and contribute data that could be interpreted appropriately.
Population	There were no restrictions on the population as long as the review was carried out in the context of the COVID‐19 pandemic.	Reviews from which data relevant to COVID‐19 could not be isolated were excluded.
Interventions	We used a draft conceptual framework developed by the University of Munich and WHO—derived from a review and mapping of existing PHSM taxonomies and literature [18]—to define and group PHSM into seven categories: active surveillance measures—these involve measures used to identify potential carriers and reduce interactions between infected and susceptible individuals. (e.g., screening, testing, or contact tracing); response measures—these involve measures taken to physically separate individuals with confirmed or suspected infections or those at a higher risk of infection from those who are vulnerable (e.g., quarantine and isolation); service measures—these pertain to adapting, canceling, or adjusting the scheduling of various services or activities to hinder the transmission of infections (e.g., changes to school or business schedules, including closures, or the use of immunity or testing or vaccination certificates); social interactions—these involve measures that aim to reshape how people interact socially (e.g., through physical distancing or restrictions on gathering); physical environment measures—these involve measures that have a direct impact on reducing individuals' exposure to infections (e.g., through the use of physical barriers, air purifiers or ventilation, or by cleaning objects and surfaces); personal protection measures—these involve measures that cover both personal protective equipment and the encouragement of specific behaviors designed to lower the risk of transmitting or contracting infections (e.g., handwashing, respiratory etiquette, face masks, face shields or gloves); movement restrictions—these involve measures altering how individuals or groups move within specific settings and between areas, including international borders (e.g., border closures or restrictions on domestic mobility).	Reviews assessing medical countermeasures, such as vaccines and therapeutics, are not considered PHSM and were therefore not eligible for inclusion. Reviews of studies that only ranked the severity or stringency of PHSM—for example, using the Oxford COVID‐19 Government Response Tracker or the COVID‐19 Severity Index—were as the information is not helpful in understanding the effects of specific PHSM or specific combinations of PHSM. Reviews of studies in which the PHSM were not prespecified in the methods section. Reviews of studies that involved nonhuman subjects
Outcomes of interest	**COVID‐19 epidemiological outcomes** Risk and incidence Transmission‐related outcomes (e.g., transmission rates, case growth, and reproduction numbers, or time to outbreak) Hospitalizations (e.g., the number of people requiring hospitalization for COVID‐19 or number of cases requiring treatment in the intensive care unit) Mortality **Unintended health and socioeconomic consequences** Non‐COVID‐19‐related health outcomes (e.g., changes in incidence of and mortality from diseases other than COVID‐19, and effects on mental health, domestic violence, nutritional status, diet, body mass index, substance use, physical activity, sleep disturbances, as well as physiological changes, and changes in road accidents or the demand for routine care) Socioeconomic outcomes (e.g., social cohesion, educational attainment, absenteeism, childhood development issues, food insecurity, homelessness or access to housing, unemployment rate, economic productivity or growth, poverty, household and individual income or financial distress, or financial support from the government) Other outcomes (e.g., environmental or ecological outcomes, or human rights–related consequences	Reviews that described PHSM interventions without reporting on any of the outcomes of interest were excluded because these could not contribute to answering the research questions related to effectiveness and unintended consequences. Reviews of studies that did not present data in a manner that would allow us to isolate the findings specific to COVID‐19 were also excluded. Reviews focusing on the overall effectiveness of the response to or unintended consequences of the COVID‐19 pandemic on specific outcomes (e.g., physical inactivity, mental health) but without specifying any particular association with PHSM were excluded.
Setting	Reviews of studies conducted in any area, setting (e.g., school, business, points of entry, gatherings) or among any population group (e.g., adults, adolescents, elderly)	Reviews that included studies conducted only in in vitro or laboratory settings (i.e., studies that did not include human participants or were not based on modeling of actual human settings) were excluded.
Publication	Published and gray literature were included. We did not restrict to any languages	Publications or preprints on servers such as medRxiv, bioRxiv, Litcovid, and the Social Science Research Network (known as SSRN) were excluded in an effort to restrict the pool to higher‐quality reviews.

*Note*: The PHSM conceptual framework has since undergone extensive stakeholder consultation and review and is expected to be published by the end of 2023 on the WHO PHSM website at https://www.who.int/activities/measuring-the-effectiveness-and-impact-of-public-health-and-social-measures.

Abbreviations: COVID‐19, coronavirus disease 2019; PHSM, public health and social measures.

### 2.2 Search methods

The primary search source was the Epistemonikos COVID‐19 Living Overview of Evidence (LOVE) living repository. This has been validated for use as a reliable single source of COVID‐19 evidence, with three validation studies indicating that the searches to populate the repository identify 100%, 93% (being the most exhaustive source of all those that were compared), and 99.67% of relevant articles. Both the comprehensiveness and the currency were 100% for randomized trials [19, 20, 21]. The database is maintained through automated or semiautomated searches from 41 databases that are updated on a daily to weekly basis. The search included reviews published between December 2019 and September 2022, and had no language restrictions.

For the purpose of this overview of reviews, the search strategy included specific keywords to represent the concept of public health and social measures. A PHSM expert reviewed the final list of terms, and a research search methods expert created the final boolean search strategy. The search strategies are available in Appendix S1. Additionally, a validated automatic classifier algorithm (a machine learning classifier for the records with an abstract and a heuristic classifier for the records without an abstract) was applied to the total records available in the COVID‐19 LOVE repository to detect relevant systematic reviews [17].

Moreover, we screened the reference lists of included reviews.

### 2.3 Selection of reviews

**Figure 1:**
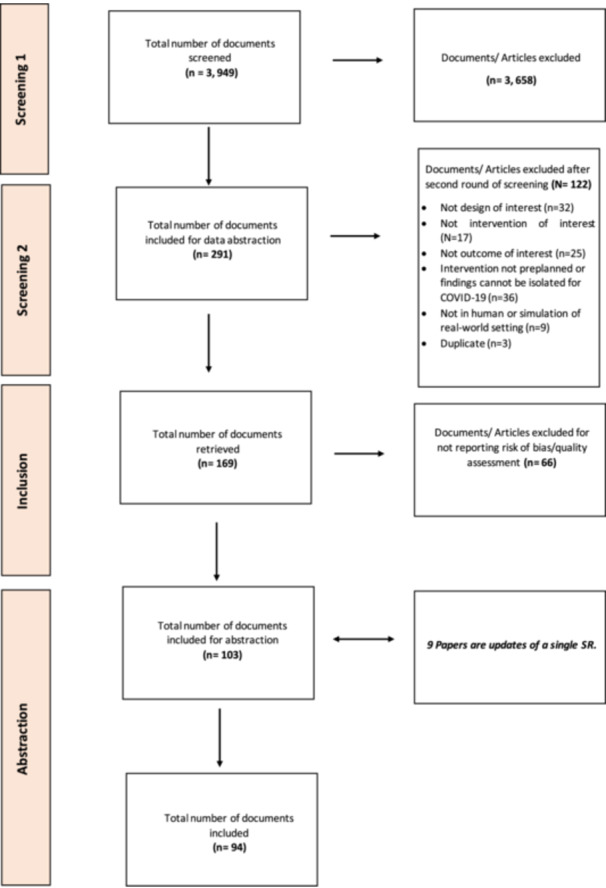
PRISMA flowchart.

The reviews found in the COVID‐19 LOVE database were uploaded to Collaboratron, the Epistemonikos screening tool, then de‐duplicated [5]. Two reviewers independently evaluated the titles, abstracts, and full text of potentially eligible studies. In cases of disagreement, they referred the study to a third reviewer. Reasons for exclusion at the full‐text stage and the study selection process are described in the PRISMA flow diagram (Figure 1).

The screening process was completed in two rounds. In round 1, we used Collaboratron to screen studies against the following two criteria:

- Does the review include data on COVID‐19?
- Does the review assess PHSM?

In round 2, we used EndNote (Clarivate) to identify the reviews that:

- met our definition of a systematic review;
- covered outcomes of interest (as identified by the WHO PHSM conceptual framework, see Table 1);
- focused on a human setting or the simulation of a human setting (i.e. did not focus on in vitro studies);
- examined preplanned interventions (i.e., interventions of interest were specified a priori in the methods), particularly for systematic reviews of association studies);
- reported data for our outcomes of interest;
- reported a risk of bias assessment of included primary studies.

Before the selection process, all reviewers participated in a calibration exercise using a randomly selected sample of 90 citations for the first round and 20 citations for the second round.

### 2.4 Methodological assessment of included reviews

Two researchers assessed independently and in duplicate the methodological limitations of the included reviews using A Measurement Tool to Assess Systematic Reviews (AMSTAR 2) [22]. AMSTAR 2 is a domain‐based rating system that has seven critical domains and nine noncritical domains.

Adherence to each domain was rated as follows: yes, partial yes, no, or not applicable. The overall confidence in the results of the review was rated as critically low, low, moderate, or high. Disagreements were resolved through discussion or with the help of a third reviewer. A decision was made to rate a review with only one partial yes as a ‘yes’, and one with two partial yeses as a ‘no’. Reviews that scored low or critically low were considered to have major methodological limitations.

### 2.5 Data abstraction

Using standardized and pilot‐tested data abstraction forms, one reviewer abstracted data, and another checked for accuracy. Disagreements were resolved through consensus or with the help of a third reviewer. A calibration exercise was conducted to ensure adequate agreement before data abstraction.

We extracted the following characteristics from each review:

- type of synthesis (e.g., standard vs. rapid review);
- basic information about each systematic review (e.g., title, authors, year of publication, date last assessed as being up to date, number of included studies, objectives, specific research question [or questions], if explicitly provided by the authors, eligibility criteria and study design);
- information about the primary studies (e.g., the types of primary study designs considered and also identified in the review);
- context (e.g., countries or area, setting [e.g., long‐term care facilities, etc.)], phase of outbreak (i.e., if reported a priori in the methods section), and population of interest at the review level;
- interventions and comparators at the review level: these were subsequently categorized using the WHO PHSM conceptual framework described earlier;
- reported outcomes in relation to our outcome categories (see Table 1), as specified in the methods section of the systematic review;
- key findings, including narratively reported study‐level data and/or data from meta‐analyses; where applicable, we also reported on potential factors that may influence the effectiveness of the PHSM;
- results of risk of bias assessment of included studies, if reported;
- GRADE (Grading of Recommendations Assessment, Development, and Evaluation) certainty of evidence assessment, if reported (this is a widely recognized and robust framework used to assess the quality of evidence and the strength of recommendations in research) [23];
- whether the risk of bias and GRADE certainty assessments were considered by the review authors in interpreting the findings of the review;
- additional information (e.g., limitations of the systematic review, conclusions, reported disclosures of interest, funding source).

### 2.6 Data synthesis

The results are reported in both tabular and narrative formats. Where applicable, the methodological limitations of the reviews are noted. Where a GRADE assessment was available for an outcome, we reported this alongside the findings.

Findings are stratified by PHSM category (as per the initial WHO conceptual framework on PHSM) and by outcome within each category. When reporting the results, precedence was given to those with higher AMSTAR 2 ratings and to those outcomes with higher GRADE ratings as these reviews and outcomes are more likely to be reliable.

## 3 RESULTS

The search retrieved 3949 citations. The selection process identified 94 unique reviews, encompassing 2147 unique primary studies (Figure 1).

### 3.1 Characteristics of included reviews

Table 2 shows the general characteristics of the 94 included reviews, of which 77 (82%) were systematic reviews and 17 (18%) were rapid reviews (see Appendix S2 for the characteristics of each included review). Of these, 24 (26%) reviews included meta‐analyses. The interventions covered by the reviews were implemented either on their own or as part of a multicomponent intervention, or both. The most frequently reported PHSM category for a single intervention was personal protection (*n* = 18, 19%) followed by active surveillance (*n* = 14; 15%). “Lockdown”, meaning the implementation of several PHSM at the same time, was the most frequently reported intervention among the reviews assessing multicomponent interventions (*n* = 39, 41%). While the term “lockdown” was often associated with severe social disruption, in 27 of these 39 reviews (69%), the authors did not provide a particular definition for lockdown or specify the components of the intervention.

**Table 2:** Characteristics of included reviews (*N* = 94).

Characteristic	Number
**Type of synthesis**	
Systematic review	77
Rapid review	17
**Design of primary studies included in reviews** a	
Randomized controlled trial	6
Quasi‐randomized controlled trial	6
Controlled before and after study	3
Uncontrolled before and after study	8
Time series	12
Case–control	21
Cohort	42
Longitudinal	15
Cross‐sectional	58
Modeling	24
Observational	10
Case series or study	6
Qualitative or phenomenological	8
Ecological	1
Other	
Descriptive	4
Quantitative	3
Prospective secondary data analysis	1
Mixed methods	1
Mechanistic	1
Natural experiment	1
Empirical	1
Parallel comparative	1
Prospective exploratory experimental	1
Repeated measures design	1
Synthetic control method	1
Event study	1
Not specified	13
**WHO geographical region**	
European	4
Western Pacific	3
Americas	1
More than one region	85
Not specified	1
**Target population** a	
General population	42
Children or adolescents	21
Adults	11
Health care workers	5
Older people	1
Others	
Individuals who had contact with people confirmed or suspected to have COVID‐19 or who traveled from a country with a declared outbreak or who live in areas with high disease transmission	4
Quarantined individuals	3
Perinatal women	2
People with pre‐existing conditions	6
Sportspersons (i.e., athletes and chess players)	1
Not specified	7
**Setting** a	
Health care facility	20
Community	16
Educational institution	14
Occupational/workplace setting	10
Point of entry (including airport, ports, land borders)	6
Household	5
Entertainment facilities (e.g., mall, stadium, gym)	5
Long‐term care facilities	2
Not specified	49
**Phase of COVID‐19 pandemic specified**	
Yes	28
No	66
**GRADE framework applied**	
Yes	16
No	78
**Meta‐analysis included**	
Yes	24
No	70
**AMSTAR 2 rating**	
High	3
Moderate	4
Low	13
Critically low	74
**Type of intervention reported** a	
***Single intervention***	30
*Active surveillance measures* b	14
Screening	4
Testing	7
Contact tracing	6
*Response measures*	15
Quarantine	14
Isolation	5
*Service measures*	11
School	8
Business	4
Measures to reduce opportunities for contact	4
*Social interactions*	9
Physical distancing	8
Stay‐at‐home orders	3
Restrictions on mass gatherings	2
*Movement restrictions* c	5
Travel‐related measures (e.g., travel bans, cancellation of flights, border closure)	5
*Physical environment measures*	3
Cleaning objects and surfaces	3
Ventilation	1
*Personal protection measures*	18
Use of face masks	13
Hand washing	3
Use of PPE	2
Eye protection	2
Respirators	1
***Combination of interventions***	44
Multicomponent intervention	34
Lockdown (i.e. combination of interventions, mostly not specified)	39
***Both single and multicomponent***	19
**Type of outcomes reported** a	
*COVID‐19 epidemiological outcomes*	40
Risk and incidence	25
Transmission‐related outcomes (e.g. transmission rates, case growth rate, reproduction number, and probability of outbreak)	30
Mortality	15
Hospitalization	4
*Non‐COVID‐19‐related health outcomes*	58
Mental health	22
Nutritional status, diet, body mass index	19
Physical activity or increase in sedentary behavior	15
Change in incidence of and mortality for diseases other than COVID‐19	6
Substance use	2
Violence	6
Sleep disturbance or other sleep‐related issues	3
Physiological changes	4
Health service utilization patterns	6
Voice parameters (e.g., perceptual, acoustic, aerodynamic, physiological)	1
Early childhood development	1
Mobility	1
*Socioeconomic outcomes*	5
Educational attainment	2
Number of days spent in school	1
Absenteeism and disability	2
Social cohesion	1
*Other outcomes (e.g., environmental or ecological outcomes and human rights–related consequences)*	1

Abbreviations: AMSTAR 2, A Measurement Tool to Assess Systematic Reviews 2; COVID‐19, coronavirus disease 2019; GRADE, Grading of Recommendations Assessment, Development and Evaluation.

^a^ Totals do not necessarily add up to 94% or 100% because some of the included reviews reported on more than one type of intervention.

^b^ Surveillance activities at the point of entry or in the workplace are included under the active surveillance category, not the movement or services categories.

^c^ Responses at the point of entry are included under the response category and not the travel‐related measures in the movement category.

The most commonly reported outcome category was non‐COVID‐19‐related health outcomes (*n* = 58, 62%), followed by COVID‐19 epidemiological outcomes (*n* = 40, 43%); the least commonly reported category was socioeconomic outcome (*n* = 5, 5%). Twenty‐three (24%) of the included reviews reported on more than one type of outcome category. Across all outcome categories, transmission‐related outcomes (*n* = 30, 32%) were the most frequently reported followed by risk and incidence (*n* = 25, 27%) and then mental health (*n* = 22, 23%).

**Figure 2:**
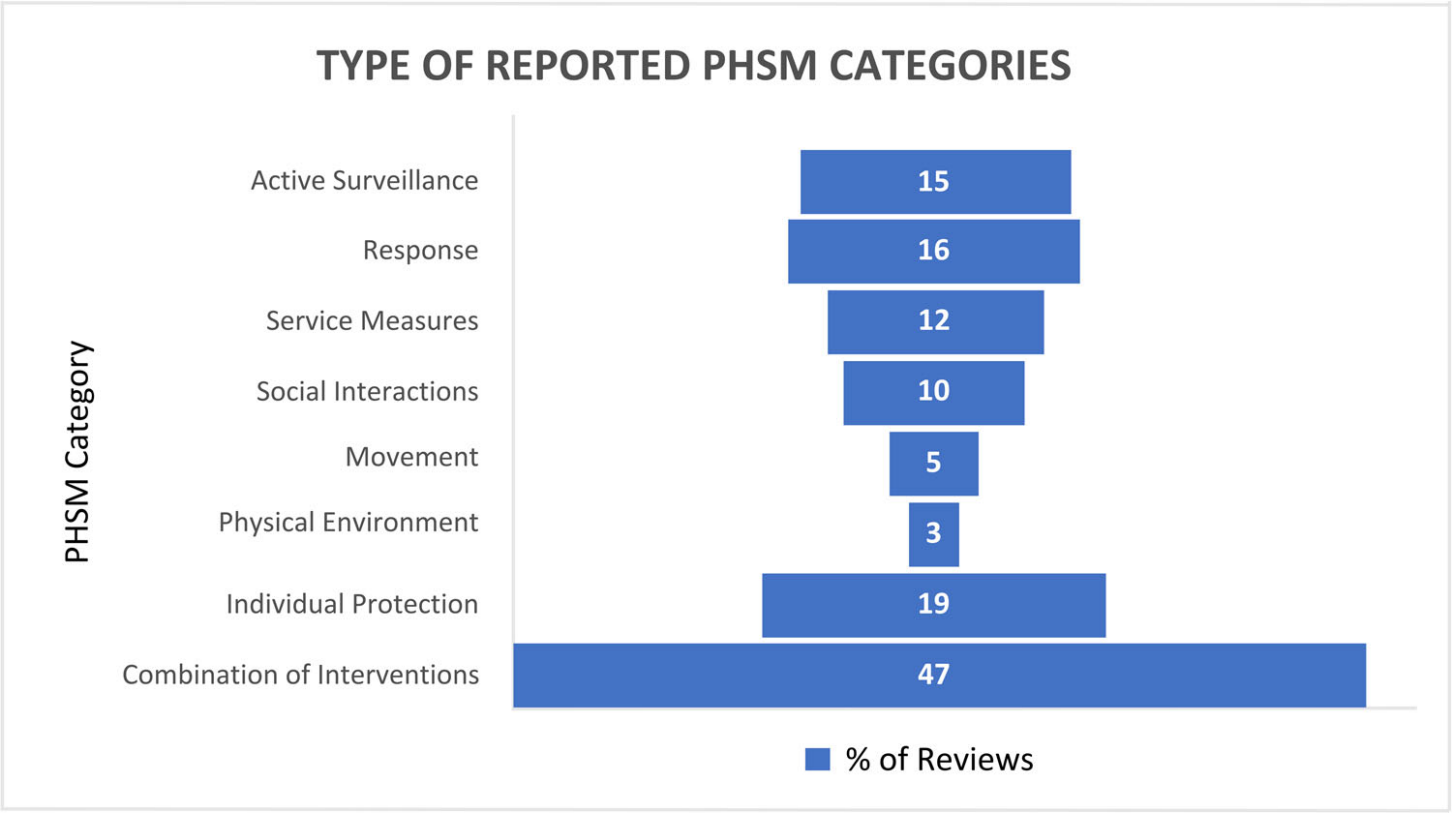
Percentage of reviews, by intervention category.

**Figure 3:**
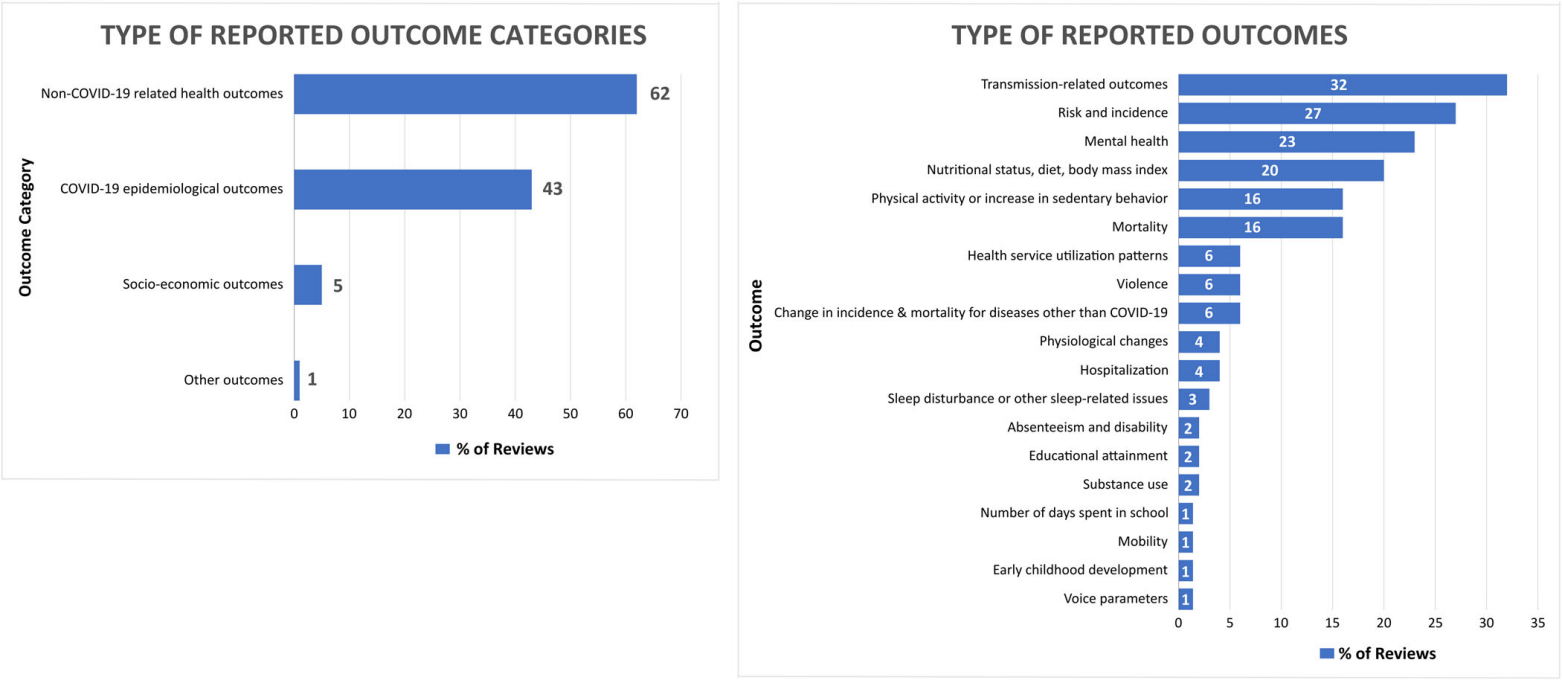
Percentage of reviews, by outcome category.

Figure 2 presents the percentage of reviews, by intervention category, and Figure 3 presents the percentage of reviews according to the category of outcomes reported.

### 3.2 Methodological assessment of included reviews

**Figure 4:**
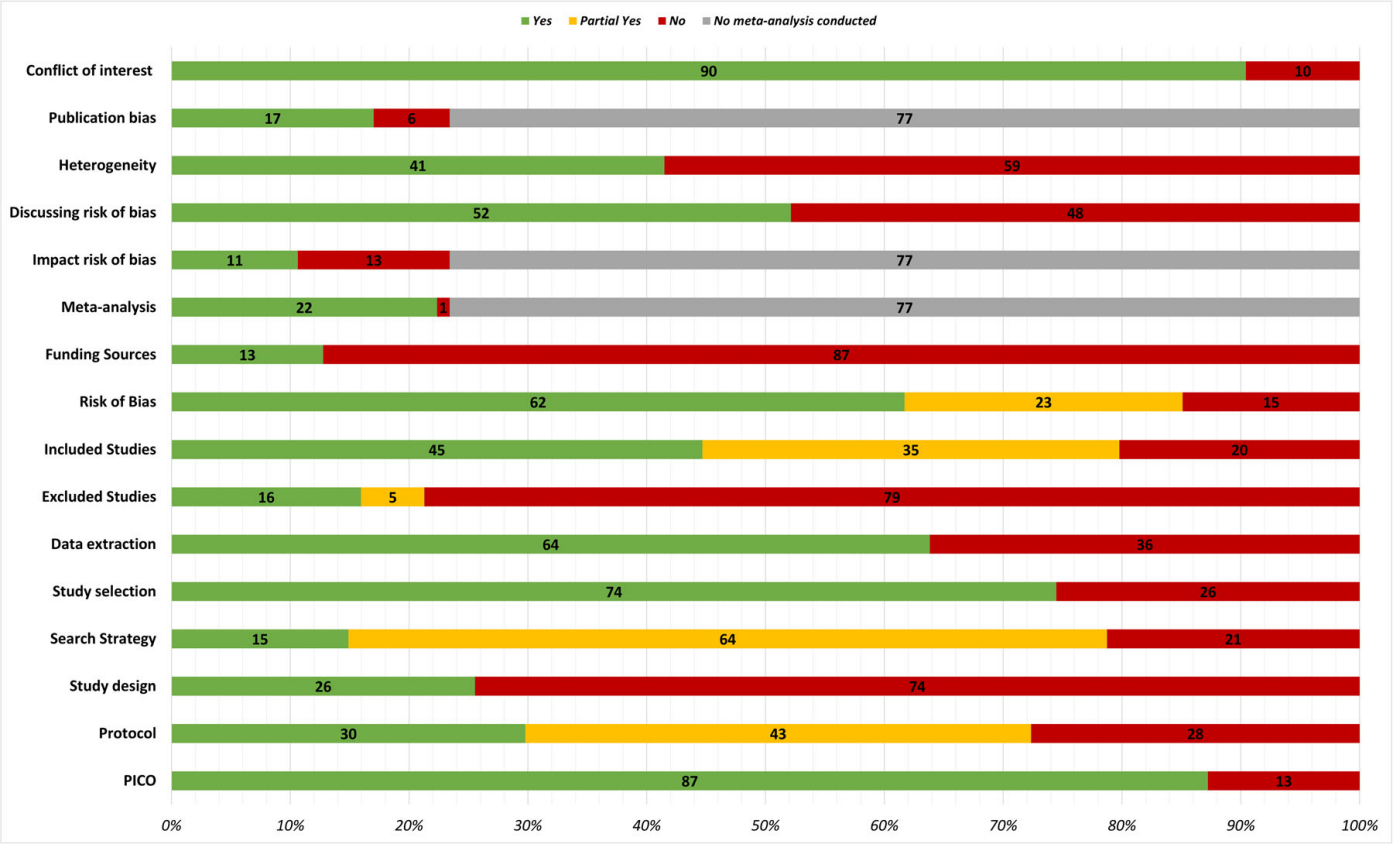
Assessment of the methodological limitations of included reviews using AMSTAR 2.

Of the 94 reviews assessed using AMSTAR 2, eight (9%; seven rapid and one systematic review) were rated as having high or moderate confidence ratings on AMSTAR 2 [24, 25, 26, 27, 28, 29, 30, 31]. The majority were rated as critically low (*n* = 73, 78%) or low (*n* = 13, 14%) (Appendix S3). All of the reviews with the high confidence rating were published by Cochrane. The critical domains that most commonly compromised the methodological quality of the included reviews were failing to refer to an a priori protocol, having a search strategy that was not comprehensive and failing to include a list of excluded studies with the justifications for exclusion (Figure 4). Indeed, 79% of included reviews were rated as “no” on the last domain. As that domain is considered critical, the reviews are automatically downgraded to low‐confidence evidence as a starting point. A sensitivity analysis using less stringent criteria for the search strategy domain did not change the results substantially: while none of the review scores changed to high confidence, 10 of the low confidence reviews changed to moderate.

Sixteen (17%) reviews reported applying the GRADE framework to assess the certainty of the evidence, and none of these reviews reported an outcome that was assessed as having a high certainty of evidence In reporting these results, we applied the GRADE guidance about informative statements [32].

### 3.3 Findings of the included reviews

A narrative summary of the findings from the eight reviews rated as having high or moderate confidence on the AMSTAR 2 instrument is provided below. Table 3 reports the direction of the results and the certainty of the evidence for the main outcomes for those high or moderate‐confidence reviews that applied the GRADE framework. In addition, Table 4 summarizes the findings from all reviews, including those rated as having low or very low confidence on the AMSTAR 2 instrument), by intervention category. In Appendix S4, we report detailed findings in both narrative and tabular formats, by intervention category. Of the eight reviews with a high or moderate confidence rating on AMSTAR 2 instrument, three focused on more than one setting, while five focused on the following specific settings: entertainment facilities, specifically for swimming‐related activities (*n* = 1); point of entry, specifically airports (*n* = 1), workplace, specifically schools (*n* = 2) and long‐term care facilities (*n* = 1).

**Table 3:** Intervention‐outcome matrix for the reviews with moderate and high confidence ratings on AMSTAR 2 that used the GRADE framework.

Public health and social measures	Direction of effects and certainty of the evidence^a^							
	New infections	Transmission rate	Imported/exported cases	Shift in pandemic	Health care utilization	Mortality	Unintended health outcomes	Socioeconomic outcomes
**Active surveillance measures**								
Routine testing of residents and staff independent of symptoms in long‐term care settings	✔⊕⊕◯◯	NF	NF	?⊕◯◯◯	✔⊕⊕◯◯	?⊕⊕◯◯	NF	NR
Symptom‐based surveillance testing in long‐term care settings	?⊕ ◯ ◯◯	NF	NF	?⊕◯◯◯	NF	NF	NF	NR
Symptom‐ or exposure‐based screening at borders	?⊕◯◯◯	NF	✔⊕⊕⊕◯^b^	?⊕◯◯◯	NF	NF	NF	NF
Test‐based screening at borders	✔⊕◯◯◯	NF	?⊕◯◯◯	NF	NF	NF	NF	NF
Test‐based attendance after contact with someone positive for COVID‐19 (vs standard 10‐day self‐isolation after exposure)	?⊕◯◯◯	NR	NR	NR	NF	NF	NF	✔⊕⊕◯◯^c^
Screening for symptoms among air travelers	NF	NF	NF	✔⊕⊕◯◯	NF	NF	NR	NR
Laboratory screening for health care workers	NF	?⊕◯◯◯	NR		NF	NF	NR	NR
Symptom‐based screening and isolation in schools	?⊕◯◯◯	NF	NF	?⊕◯◯◯	NF	NF	NF	NF
Mass testing and isolation	?⊕◯◯◯	NF	NF	?⊕◯◯◯	?⊕◯◯◯	?⊕◯◯◯	NF	?⊕◯◯◯^d^
Testing of people newly admitted to long‐term care facilities	?⊕◯◯◯	NF	NF	NF	NF	NF	NF	NR
**Response measures**								
Separation of infected and noninfected residents and staff caring for infected and noninfected residents in long‐term care facilities	?⊕◯◯◯	NF	NF	✔⊕⊕◯◯	NF	?⊕ ◯ ◯◯	NF	NR
Isolating cases in long‐term care facilities	?⊕◯◯◯	NF	NF	?⊕◯◯◯	NF	NF	NF	NR
Quarantining people newly admitted to long‐term care facilities	? ⊕◯◯◯^b^	NF	NF	NR	NF	NF	NF	NR
Quarantining travelers	?⊕◯◯◯^e^	NF	?⊕◯◯◯	✔ ⊕⊕◯◯	NF	NF	NF	NF
Quarantining and screening at borders vs implementing a single measure	NF	NF	NF	?⊕◯◯◯	NF	NF	NF	NF
**Service measures**								
Changing the length of the school day	NF	NF	NF	?⊕◯◯◯	NF	NF	NF	NF
Reducing the number of students in a school	?⊕◯◯◯	NF	NF	?⊕◯◯◯	?⊕◯◯◯	?⊕◯◯◯	NF	?⊕◯◯◯^d^
Reducing the number of contacts between students in a school	?⊕◯◯◯	NF	NF	?⊕◯◯◯	?⊕◯◯◯	NF	NF	?⊕◯◯◯^d^
Cohorting residents and staff in long‐term care facilities	?⊕◯◯◯	NF	NF	?⊕◯◯◯	NF	NF	NF	NR
Reducing contacts in long‐term care facilities	✔✕ ⊕◯◯◯	NF	NF	?⊕◯◯◯	NF	NF	NF	NR
Intensifying testing of residents and staff readmitted after holidays to long‐term care facilities	?⊕◯◯◯	NF	NF	NF	NF	NF	NF	NR
Restricting the admission of new residents to long‐term care facilities	?⊕◯◯◯	NF	NF	NF	NF	NF	NF	NR
Restricting admission for visitors to long‐term care facilities	?⊕◯◯◯	NF	NF	NF	NF	?⊕◯◯◯	✔✕ ⊕◯◯◯^f^	NR
**Movement restrictions**								
Travel restrictions reducing or stopping cross‐border travel	?⊕◯◯	NF	?⊕◯◯◯	?⊕◯◯◯	NF	?⊕◯◯◯	NF	NF
**Physical environment measures**								
Implementing cleaning and environmental hygiene measures in long‐term care facilities	?⊕◯◯◯	NF	NF	NF	NF	NF	NF	NR
Implementing more frequent cleaning in long‐term care facilities	?⊕◯◯◯	NF	NF	?⊕◯◯◯	NF	NF	NF	NR
Implementing an enhanced cleaning policy in schools	NF	NF	NF	?⊕◯◯◯	NF	NF	NF	NF
Improving ventilation in schools	NF	?⊕◯◯◯^g^	NF	NF	NF	NF	NF	NF
**Personal protection measures**								
Implementing personal hygiene measures in long‐term care facilities	NF	NF	NF	?⊕◯◯◯	NF	NF	NF	NR
Using masks and personal protective equipment in long‐term care facilities	?⊕◯◯ ◯	NF	NF	?⊕◯◯◯	NF	?⊕◯◯◯	NF	NR
Using barrier nursing in long‐term care facilities	?⊕◯◯◯	NF	NF	?⊕◯◯◯	NF	NF	NF	NR
Using face masks in schools	?⊕◯◯◯	NF	NF	?⊕◯◯◯	?⊕◯◯◯	?⊕◯◯◯	NF	NF
Implementing handwashing in schools	NF	NF	NF	?⊕◯◯◯	NF	NF	✔ ⊕⊕◯◯^f^	NF
Using combined infection prevention and control measures in schools	?⊕◯◯◯	NF	NF	?⊕◯◯◯	?⊕◯◯◯	?⊕◯◯◯	NF	?⊕◯◯◯^d^
**Multicomponent interventions**								
Implementing multicomponent interventions in long‐term care facilities	✔ ⊕⊕◯◯	NF	NF	NF	NF	?⊕◯◯◯	NF	NR
Implementing multicomponent interventions to make contacts safer in schools	✔✕ ⊕⊕◯◯^i^	NF	NR	NF	NF	NF	NF	NF
Implementing multicomponent interventions in swimming facilities	NF	∅⊕◯◯◯	NR	NR	NR	NR	NR	NR

^a^ The symbols indicate the following:

Direction of effects: ✓: desirable effect; ∅: little or no effect;?: an uncertain effect; ✕: an undesirable effect; NF: no studies found by this review that reported this outcome; NR: outcome not reported by this review.

Certainty of evidence:

⊕⊕⊕⊕: High certainty evidence; this research provides a very good indication of the likely effect. The likelihood that the effect will be substantially different is low.

⊕⊕⊕⊖: Moderate certainty evidence; this research provides a good indication of the likely effect. The likelihood that the effect will be substantially different is moderate.

⊕⊕⊖⊖: low certainty evidence; this research provides some indication of the likely effect. However, the likelihood that it will be substantially different is high.

⊕⊖⊖⊖: Very low certainty evidence; this research does not provide a reliable indication of the likely effect. The likelihood that the effect will be substantially different is very high.

Additional clarifications:

^b^ This was based on one modeling study from China.

^c^ Unintended socioeconomic outcome related to absenteeism.

^d^ Unintended socioeconomic outcomes relaed to number of days spent in school.

^e^ Certainty of evidence from the modeling study was rated as “low” while the certainty from an observational study was rated as “very low.” We considered the latter.

^f^ Unintended health outcome related to mental health.

^g^ Outcome related to inhaled dose of particles containing RNA virus.

^h^ Unintended health outcome related to hand eczema.

^i^ Certainty of evidence from observational/modeling studies was rated as “low,” while the certainty from the modeling study was rated as “very low.”

The following intervention categories were examined by the eight reviews: active surveillance (i.e., screening and testing) (*n* = 6); response strategies (i.e., quarantine and isolation protocols) (*n* = 3); service measures (e.g., staggered arrival, break and departure times; cohorting measures in schools and long‐term care facilities, and restrictions on visits) (*n* = 2); movement restrictions (e.g., international travel restrictions, travel bans, and cross‐border travel restrictions) (*n* = 1); physical environment (e.g., enhanced ventilation, meticulous cleaning of objects and surfaces, and more frequent cleaning routines) (*n* = 2); and personal protection (e.g., personal hygiene practices, use of face masks and personal protective equipment) (*n* = 1). Four of the reviews examined multicomponent interventions.

The outcome categories reported included COVID‐19 epidemiological outcomes (*n* = 8), non‐COVID‐19‐related health outcomes (*n* = 2), and socioeconomic outcomes (*n* = 1). The most commonly reported COVID‐19 epidemiological outcomes were transmission‐related outcomes. The non‐COVID‐19‐related health outcomes focused on mental health and physiological outcomes (e.g., the risk of developing hand eczema from handwashing), while the socioeconomic outcomes focused on absenteeism among school staff and students. Four of the reviews reported on more than one outcome.

Of the eight reviews with a high or moderate confidence rating, none conducted a meta‐analysis and six applied the GRADE framework (refer to Table 3). The number of randomized controlled trials (RCTs) included per review ranged from none (*n* = 5) to one (*n* = 3).

The evidence synthesized by these eight reviews is briefly summarized below by intervention category. These reviews largely rated the certainty of evidence for the assessed outcomes as low to very low, with none rated as high certainty. This means that we are uncertain about the true effect.

The reviews looking at active surveillance measures [24, 25, 27, 33, 108] found that:

- *routine testing* of residents and staff independent of symptoms may reduce the number of infections and hospitalizations in long‐term care facilities. Routine testing may also reduce the number of deaths among residents, but it is uncertain whether it reduces deaths among staff or whether it increases or decreases the probability of outbreaks. Additionally, it is uncertain whether *symptom‐based surveillance testing* in long‐term care facilities reduces infections and lowers the likelihood of outbreaks, as well as the number of deaths among residents;
- it is uncertain whether screening health care workers in emergency departments using laboratory tests reduces transmission to patients and other health care workers;
- screening at borders based on symptoms or exposure probably reduces the number of cases exported and may also slightly slow but not stop the importation of cases, but it is uncertain whether the measure delays outbreaks. It is also uncertain whether polymerase chain reaction (PCR) testing at borders as a screening measure reduces the number of infected people admitted to or leaving from an area as well as the number of secondary cases;
- there is uncertainty regarding the effectiveness of rapid antigen testing for screening asymptomatic individuals (i.e., population‐level screening, pre‐event screening, and serial testing) to mitigate the transmission of COVID‐19;
- within schools, it is uncertain whether a test‐based attendance policy affects the incidence of PCR‐positive COVID‐19 infection (i.e., any infection, symptomatic infection) compared to standard 10‐day self‐isolation. Furthermore, test‐based attendance may result in little to no difference in the number of days of absence.

The reviews looking at response measures [24, 26] found that:

- in long‐term care facilities, quarantining newly admitted patients as well as confinement of staff with residents may reduce the number of infections. The latter measure may also reduce the number of deaths;
- quarantining people at borders may delay the time to outbreak, but it is uncertain whether it increases or decreases the number and proportion of cases and proportion of cases imported;
- in long‐term care facilities, it is uncertain whether isolating cases reduces the number of infections or probability of outbreak;
  The reviews looking at combined active surveillance and response measures [24, 31] found that:
- combining screening and quarantine at borders may increase the proportion of cases detected and reduce the number of days a person is at risk of transmitting the infection to the community;
- within school settings, it is uncertain whether mass testing and isolation or symptomatic screening and isolation reduce transmission‐related outcomes, hospitalization, and the number of days spent in school.
  The reviews looking at service measures [26, 31] found that:
- restricting visis to long‐term care facilities may reduce the number of infections and the number of deaths, but it is uncertain whether the measure increases the probability of facility contamination. It is also uncertain how such restrictions adversely affect the mental health of residents;
- in long‐term care facilities, separating infected and noninfected residents and staff caring for them may reduce the number of infections and the probability of outbreaks, but it is uncertain whether it reduces number of deaths. It is also uncertain whether cohorting residents and staff decreases the number of infections and the probability of outbreaks;
- it is uncertain whether reducing class sizes or implementing alternate days for attendance in schools reduces transmission‐related outcomes and health care utilization (i.e., hospitalizations and admissions to intensive care units).
  The reviews looking at personal protection measures [26, 31] found that:
- in long‐term care facilities, it is uncertain whether the use of masks and personal protective equipment (PPE) by staff reduces the number of infections, the probability of outbreaks, or the number of deaths. It is also uncertain whether personal hygiene measures reduce the probability of outbreaks;
- it is uncertain whether barrier nursing in long‐term care facilities increases the number of infections or the probability of outbreaks. Barrier nursing refers to a set of stringent infection control techniques (ranging from PPE use by staff to strict isolation of infected individuals using airlocks) intended to protect nursing staff against infection;
- it is uncertain whether the use of face masks in schools reduces transmission‐related outcomes (i.e., the number or proportion of cases, the reproduction number, or the number or proportion of deaths) and health care utilization (i.e., the number or proportion of hospitalizations);
- it is also uncertain whether handwashing in schools affects the reproduction number, but it may increase hand eczema among children.

The reviews looking at the physical environmental measures [26, 31] found that:

- it is uncertain whether cleaning or environmental hygiene measures in long‐term care facilities or schools reduces the number of infections or whether more frequent cleaning increases or decreases the number of infections;
- it is uncertain whether improving ventilation by using air purifiers equipped with high‐efficiency particulate air filters in a high school classroom reduces the concentration of aerosol particles containing RNA viruses in the room and the inhaled dose of RNA virus for a susceptible person.

The reviews looking at movement restriction measures [31] found that:

- the certainty of the evidence for most travel‐related measures and outcomes is very low, and the true effects are likely to be substantially different from those reported in the review;
- findings in many of the included studies suggest that the effects depend on factors such as the level of community transmission, travel volumes, and duration, other public health measures in place and the exact specification and timing of the measure.
  The reviews looking at multicomponent PHSM [26, 29, 30, 31] found that:
- there is low‐certainty evidence supporting the implementation of multicomponent PHSM in different settings. Most of the reviews reported better outcomes when several measures were combined compared with implementation of a single measure alone:
  − within long‐term care facilities, a combination of entry regulation, transmission and contact control, surveillance, and outbreak control measures may reduce the number of infections, but it is unclear whether they reduce the number of deaths;
  − multicomponent PHSM at mass gatherings may reduce the risk of COVID‐19 transmission; however, it is unlikely that this risk can be eliminated entirely. All studies adopted a layered mitigation approach involving multiple measures and were rated as being of only fair to poor quality. The most commonly implemented measures were providing hand sanitizer, wearing face masks, and ensuring adequate ventilation, health screening and contact tracing;
  − evidence about multicomponent PHSM in schools was mixed; while most of the included studies suggested PHSM may decrease the number or proportion of cases, some showed mixed or no effects. Measures included a combination of physical distancing, modification of school activities, testing, exemptions for high‐risk students, hand hygiene and mask‐wearing;
  − within swimming pool facilities, it is uncertain whether there is an association between compliance with a range of precautionary restrictions and COVID‐19 transmission during swimming‐related activities.

**Table 4:** Summary of findings from all reviews, by intervention category, with GRADE assessment noted, if applicable (*N* = 94).

Public health and social measures (number of reviews)	Epideomiological outcomes	Unintended health and socioeconomic consequences
Active surveillance (*n* = 13)	**Screening at borders** Symptom‐ or exposure‐based screening measures at borders probably reduce the number of cases exported, but it is uncertain whether they delay outbreaks (one high‐confidence review, GRADE applied) [24]. Symptom‐based screening at travel hubs may slightly slow but not stop the importation of infection (one moderate confidence review, GRADE applied) [27]. It is uncertain whether PCR testing at borders as a screening measure reduces imported or exported cases as well as secondary cases (one high‐confidence review, GRADE applied) [24]. **Screening in health care settings** It is uncertain whether laboratory screening of health care workers reduces COVID‐19 transmission to patients and other health care workers in an emergency department (one moderate‐confidence review, GRADE applied) [27]. **Population‐level and pre‐event screening** There is uncertainty surrounding the effectiveness of rapid antigen testing for screening asymptomatic individuals to mitigate the transmission of COVID‐19 (one moderate confidence review) [33]. Thermal screening may be ineffective in restraining the spread of COVID‐19 due to the presence of asymptomatic or presymptomatic cases (two low‐confidence reviews) [34, 35]. **Testing in health care settings** Routine testing of residents and staff in long‐term care facilities independent of symptoms may reduce the number of infections. Evidence from one observational study suggests that such testing may reduce the number of infections, while evidence from one modeling study suggests that it probably reduces hospitalizations. The measure may also reduce the number of deaths among residents, but it is uncertain whether it reduces deaths among staff or whether it increases or decreases the probability of outbreaks (one high‐confidence review, GRADE applied) [26]. It is uncertain whether symptom‐based surveillance testing in long‐term care settings reduces infections and lowers the likelihood of outbreaks as well as the number of deaths among residents (one high‐confidence review, GRADE applied) [26]. PCR testing of health workers may reduce the risk and incidence of COVID‐19 in health care settings (one critically low‐confidence review)[36]. **Testing at mass gatherings** Mass testing may reduce the number of infections and mortality from COVID‐19; however, there is insufficient evidence to determine the effectiveness of mass testing without other measures, such as lockdowns (one critically low‐confidence review) [37]. **Testing in schools** It is uncertain whether a test‐based attendance policy affects rates of symptomatic infection or any PCR‐positive SARS‐CoV‐2 infection compared with standard 10‐day self‐isolation among school and college staff (one high‐confidence review, GRADE applied) [25]. **Contact tracing** Contact tracing may reduce the risk, incidence, COVID‐19 case growth rate, reproductive number, and mortality related to COVID‐19 (one low‐ and two critically low‐confidence reviews) [38, 39, 40]. **Testing versus combined IPC measures** Combined IPC measures may reduce COVID‐19 positivity rates among employees compared with PCR testing for asymptomatic individuals (one critically low‐confidence review) [41].	Test‐based attendance policy in schools may result in little to no difference in absence rates compared with standard 10‐day self‐isolation among school and college staff (one high confidence review) [25].
Response (*n* = 15)	**Quarantine in long‐term care settings** Quarantining new residents in long‐term care facilities may reduce the number of infections (one high‐confidence review, GRADE applied) [26]. Confinement of staff with residents in long‐term care facilities may reduce the number of infections and number of deaths (one high‐confidence review, GRADE applied) [26]. **Quarantine at borders** Quarantining people at borders may delay the time to outbreak, but it is uncertain whether it increases or decreases the number or proportion of cases and the proportion of imported cases (one high‐confidence review, GRADE applied) [24]. **Quarantine at population level** Quarantine implemented as self‐quarantine and group quarantine may reduce the incidence of infection, shorten the duration of the epidemic, and avert deaths among populations at risk for and affected by COVID‐19. Implementation of early quarantine measures may make the strategy more cost‐effective (one low‐confidence review) [42]. **Isolation in long‐term care settings** It is uncertain whether isolating cases reduces the number of infections or probability of outbreaks in long‐term care facilities (one high‐confidence review, GRADE applied) [26]. **Combined surveillance and response measures** Combining quarantine and screening at borders may increase the proportion of cases detected and reduce the number of days individuals are at risk of transmitting the infection to the community, but it is uncertain whether it delays the time to outbreak (one high‐confidence review, GRADE applied) [24]. In schools it is uncertain whether mass testing and isolation or symptomatic screening and isolation reduce transmission‐related outcomes (i.e., the number or proportion of cases, the reproduction number, the number or proportion of deaths, or shift pandemic development), hospitalizations and the number of days spent in school (one high‐confidence review, GRADE applied) [31]. Quarantine and isolation may reduce the incidence, transmission rate, case growth rate, and mortality related to COVID‐19 (four critically low‐confidence reviews) [35, 43, 44, 45].	**Quarantine** Quarantine may be associated with psychological issues including stress, anxiety, posttraumatic stress disorder, and disruptions in sleep patterns that affect both children and adults, as well as an increase in alcohol use in certain segments of the population (one low‐ and seven critically low‐confidence reviews) [42, 46, 47, 48, 49, 50, 51, 52]. Quarantine may be associated with long absences from work among health care workers (one critically low‐confidence review) [51].
Service measures (*n* = 11)	**School and business closures** School closures may reduce transmission rates, the case growth rate, reproduction number, and mortality related to COVID‐19, while the evidence is mixed for the incidence of COVID‐19 (one low‐confidence review and one very‐low‐confidence review) [29, 35]. Combined school and business closures and bans on public events may reduce the risk, incidence, transmission rate, case growth rate, reproduction number, and mortality related to COVID‐19 (three critically low‐confidence reviews) [35, 53, 54]. **Measures reducing the opportunities for contact** Restricting visitors to long‐term care settings may reduce the numbers of infections and deaths, but it is uncertain whether it increases the probability of facility contamination (one high‐confidence review, GRADE applied) [26]. In long‐term care settings, separating infected and noninfected residents and the staff caring for them may reduce the number of infections and the probability of outbreaks, but it is uncertain whether it reduces number of deaths (one high‐confidence review, GRADE applied) [26]. It is uncertain whether restricting the admission of new residents to long‐term care settings reduces the number of infections, but it may reduce the probability of facility contamination (one high‐confidence review, GRADE applied) [26]. It is uncertain whether cohorting residents and staff in long‐term care settings increases or decreases the number of infections or how measures to reduce contact affect the probability of outbreaks (one high‐confidence review, GRADE applied) [26]. Implementing visitor restrictions in health care facilities may reduce the transmission of COVID‐19 when family members follow the restrictions (one critically low‐confidence review) [55]. It is uncertain whether reducing class sizes or alternating attendance in schools reduces transmission‐related outcomes and health care utilization (e.g., hospitalizations and admissions to intensive care units) (one high‐confidence review, GRADE applied) [31].	**School and business closures** School closures may be negatively associated with mental health, physical activity, and nutrition among children and adolescents (three critically low‐confidence reviews) [56, 57, 58]. School closures may be associated with a significant decline in the number of hospital admissions and visits to pediatric emergency departments (one critically low‐confidence review) [57]. School closures may be negatively associated with students' academic achievement (in mathematics, reading, and science), as well as a loss of access to school‐based health care services, special services for children with disabilities, and nutrition programs (two critically low‐confidence reviews) [57, 59]. Closures of common facilities may be negatively associated with physical activity (one critically low‐confidence review) [58]. **Measures reducing the opportunities for contact** it is uncertain whether alternating attendance and reducing class sizes affect the number of days spent in school (one high‐confidence review, GRADE applied)[60]. It is uncertain how restricting admissions for visitors to long‐term care settings adversely affects the mental health of residents (one high‐confidence review) [26]. Implementing visitor restrictions in health care facilities may be negatively associated with physiological outcomes (increased physical pain and symptoms), nutrition intake, physical activity, mental health disorders, family relations, and provision of care by providers (e.g., burdens of ethical dilemmas, learning new technical means to enable social interaction, increased demand for communication with families) (one critically low‐confidence review) [55].
Social interactions (*n* = 9)	**Physical distancing** Physical distancing may reduce the risk and incidence of COVID‐19, as well as transmission‐related outcomes (three critically low‐confidence reviews) [35, 45, 61]. **Stay‐at‐home orders** Stay‐at‐home orders may decrease the daily death growth rate and daily case growth rate in communities (one critically low‐confidence review) [62]. **Combined social interaction measures** Combining social interaction measures may improve transmission‐related outcomes (three critically low‐confidence reviews) [53, 54, 63].	**Physical distancing** Physical distancing may be associated with an increase in mental health disorders among children and adolescents (one critically low‐confidence review) [56]. **Stay‐at‐home orders** Stay‐at‐home orders may reduce mobility by increasing the time spent at home and reducing visits to shops and workplaces and the use of public transport, thus fostering behaviors that prevent transmission (one critically low‐confidence review) [62]. **Combined social interaction measures** Combined social interaction measures may be associated with an increase in depression, stress, and anxiety (one critically low‐confidence review) [64].
Movement (*N* = 5)	**Travel restrictions** It is uncertain whether reducing or stopping cross‐border travel decreases COVID‐19 cases in the community, the number of cases exported or imported, and deaths. It is also uncertain whether controlling cross‐border travel slows the spread of COVID‐19 (one high‐confidence review, GRADE applied) [24]. There is mixed evidence for the effect of travel bans on rates of COVID‐19 incidence and transmission; travel bans may be particularly useful in the early stage of an outbreak before there is widespread distribution of the disease (four critically low‐confidence reviews) [35, 53, 63, 65]. **International vs national travel restrictions** Policies pertaining to international travel restrictions may be more effective than those pertaining to domestic or national travel restrictions (one critically low‐confidence review) [53].	**None reported**
Physical environment (*n* = 3)	**Cleaning and environmental hygiene measures in long‐term care settings** It is uncertain whether implementing cleaning or environmental hygiene measures in long‐term care facilities reduces the number of infections or whether more frequent cleaning increases or decreases the number of infections (one high‐confidence review, GRADE applied) [26]. Implementing multicomponent cleaning and environmental hygiene measures in long‐term care facilities may reduce the number of infections in these settings, but it is uncertain whether it decreases the number of deaths (one high‐confidence review, GRADE applied) [26]. **Cleaning in schools** It is uncertain whether cleaning in schools reduces the reproduction number (one high‐confidence review, GRADE applied) [60]. **Household cleaning** The use of disinfectants may be associated with a reduced risk of secondary transmission of COVID‐19 within households (one critically low‐confidence review)[35]. **Ventilation** It is uncertain whether ventilating classrooms reduces the concentration of aerosol particles containing RNA virus in the room and the inhaled dose of RNA virus for a susceptible person (one high‐confidence review, GRADE applied) [60].	**None reported**
Personal protection(*n* = 18)	**Face masks** It is uncertain whether the use of face masks in schools reduces transmission‐related outcomes (i.e., the number or proportion of cases, the reproduction number, or the number or proportion of deaths) and health care utilization (i.e., the number or proportion of people hospitalized) (one high‐confidence review, GRADE applied) [60]. Masking alone may not provide sufficient protection from COVID‐19 outbreaks in the workplace (two critically low‐confidence reviews) [41, 66]. The use of face masks in general or in community settings (e.g., mandatory masking policies) may reduce the risk, incidence, transmission‐related outcomes, and mortality related to COVID‐19 (seven critically low‐confidence review) [35, 45, 53, 54, 67, 68, 69]. **Face mask and PPE** It is uncertain whether the use of face masks and PPE by staff reduces the number of infections, the probability of outbreaks, or the number of deaths in long‐term care facilities. It is also uncertain whether personal hygiene measures reduce the probability of outbreaks (one high‐confidence review, GRADE applied) [26]. It is uncertain whether barrier nursing in long‐term care facilities increases the number of infections and the probability of outbreaks (one high‐confidence review, GRADE applied) [26]. **Handwashing** It is uncertain whether handwashing in schools affects the reproduction number (one high‐confidence review, GRADE applied) [60]. Handwashing may reduce risk, incidence, and transmission rates related to COVID‐19 (three critically low‐confidence reviews) [35, 70, 71]. **Universal masking vs combined IPC measures** Implementing combined IPC measures (incorporating swift contact tracing and case isolation, PPE, and facility zoning) may result in lower employee COVID‐19 positivity rates than single measures, such as universal masking (one critically low‐confidence review) [41]. **Eye protection** The use of eye protection (including face shields, goggles, or modified snorkel masks) may reduce the risk and incidence of COVID‐19 (one low‐confidence review) [72]. **Hand sanitizer** Hand sanitizer may reduce transmission rates related to COVID‐19 (one critically low‐confidence review) [71].	**Face masks** The use of face masks may not be associated with mobility or voice production (e.g., perceptual, acoustic, aerodynamic, physiological) (one critically low‐confidence review) [62]. The use of face masks may be associated with physiological outcomes: face mask use during exercise may be associated with increased heart rate, perceived exertion and dyspnea, and reduced pulmonary function parameters (one critically low‐confidence review) [66]. **PPE** The use of PPE may be associated with adverse events among health care workers (i.e., headache, dry skin, dyspnea, pressure injuries, itching, hyperhidrosis, dermatitis) (one critically low‐confidence review) [73]. **Handwashing** Handwashing may increase hand eczema (one high‐confidence review, GRADE applied) [60]. **Hand sanitizer** There may be a risk of toxicity from hand sanitizer due to absorption of the disinfectant through dermal contact and accidental ingestion (one critically low‐confidence review) [71].
Multicomponent(*n* = 36)	Implementing multicomponent PHSM in long‐term care facilities may reduce the number of infections, but it is unclear whether they reduce the number of deaths (one high‐confidence review, GRADE applied) [24]. Implementing multicomponent PHSM at mass gatherings may reduce the risk of COVID‐19 transmission; however, it is unlikely that this risk can be eliminated entirely. All studies adopted a layered mitigation approach involving multiple measures (one moderate‐confidence review) [29]. Evidence for multicomponent PHSM in schools was mixed; while most studies suggested PHSM may decrease the number or proportion of cases, some showed mixed or no effects (one high‐confidence review, GRADE applied) [60]. Multicomponent PHSM may reduce the risk, incidence, and transmission rates, case growth rate, reproduction number, and mortality related to COVID‐19 (13 critically low‐confidence reviews) [15, 35, 37, 41, 43, 44, 45, 54, 63, 70, 74, 75, 76]. Multicomponent PHSM may also be associated with a reduction in admissions to intensive care units for people with COVID‐19 (one critically low‐confidence review) [54].	Multicomponent interventions may be associated with changes in food intake and eating behaviors, and an increase in the consumption of unhealthy food (three critically low‐confidence reviews) [77, 78, 79]. Multicomponent interventions may be associated with decreases in physical activity and mobility and increases in sedentary behavior and screen time during the COVID‐19 pandemic, especially among children and youth (four critically low‐confidence reviews) [62, 78, 79, 80]. Multicomponent interventions may be associated with an increased prevalence of anxiety, depression, stress, suicide, and suicidal ideation, as well as other emotional and psychological concerns (eight critically low‐confidence reviews) [11, 56, 57, 78, 79, 81, 82, 83]. Multicomponent interventions may be associated with an increase in sleep problems among children and adolescents (two critically low‐confidence reviews) [78, 79]. Multicomponent interventions may be associated with an increase in substance use and, in particular, alcohol use (two critically low‐confidence reviews) [52, 84]. Multicomponent interventions may be associated with a decrease in health service utilization, including a decrease in emergency department visits, hospital admissions, vaccination, and access to psychiatric services (four critically low‐confidence reviews) [54, 57, 78, 85]. Multicomponent interventions may be associated with increased violence as well as a decrease in child abuse referrals and notifications during the COVID‐19 pandemic (five critically low‐confidence reviews) [78, 79, 83, 86, 87]. Multicomponent interventions may be associated with a reduction in influenza mortality and morbidity (one critically low‐confidence review) [88] and a reduction in preterm birth during the COVID‐19 pandemic (one critically low‐confidence review) [85].
Lockdowns (*n* = 39)	Lockdowns may reduce the risk, incidence, and transmission rates, and mortality related to COVID‐19 (six critically low‐confidence reviews) [35, 37, 43, 53, 54, 89].	Lockdowns may be associated with a general decrease in physical activity and increase in sedentary behavior across several populations, including children and patients with a variety of medical conditions (nine critically low‐confidence reviews) [83, 90, 91, 92, 93, 94, 95, 96]. Lockdowns may be negatively associated with mental health in adult population, including with an increased prevalence of anxiety, depression, stress, and suicidal ideation (eight critically low‐confidence reviews) [28, 46, 56, 82, 83, 97, 98, 99]. They may also be associated with children experiencing negative emotional symptoms (irritability, inattention, anxiety, depression, fear, boredom) as well as poor sleep (five critically low‐confidence reviews) [56, 97, 100, 101, 102]. Lockdowns may be associated with increased prevalence of domestic violence cases (one critically low confidence review) [87]. Lockdowns may be negatively associated with health care service utilization, including interruptions in cancer screening, a decrease in emergency department visits, decreases in referrals for medical examinations for child protection and immunizations (two critically low‐confidence reviews) [63, 83]. They may also be associated with delays in diagnoses and increases in avoidable deaths from cancer (one critically low‐confidence review) [103]. Lockdown may not be associated with a deterioration in glucose control or may be associated with improvement in many glucose control parameters for patients with type 1 diabetes [104, 105]. Findings were mixed for nutrition‐related outcomes, suggesting increased snacking and consumption of unhealthy food, no association with food consumption, or a positive association with increased adherence to the Mediterranean diet, no association or positive associations while negative association (four critically low‐confidence reviews) [84, 90, 106, 107]. Mixed association with alcohol use, while negative association with heavy episodic drinking and the proportion of people with problematic alcohol use [58, 84, 108]

Abbreviations: GRADE, Grading of Recommendations Assessment, Development, and Evaluation; IPC, infection prevention and control; PCR, polymerase chain reaction; PPE, personal protective equipment; SARS‐CoV‐2, severe acute respiratory syndrome coronavirus 2.

Across the different intervention categories, we identified a range of factors that may contribute to variation in the direction or magnitude, or both, of PHSM. These include the level of integration with other PHSM, the timing of implementation of the measure, the level of community transmission, the susceptibility of target populations, and the level of adherence to and enforcement of the measure. Table 5 provides a list of tentative factors that may influence the effectiveness of PHSM, based on the reviews included. These factors were derived from the reviews, highlighting those consistently mentioned across multiple studies as potentially influential.

**Table 5:** Overview of tentative factors that may influence the effectiveness of PHSM.

Intervention	Tentative factors that may influence the effectiveness of interventions
Active surveillance measures	Symptom/exposure screening alone seems to detect some COVID‐19 cases but may not prevent new cases within the protected region. Community prevalence can offer insights into the usefulness of general screening for symptoms. PCR testing, when used as a screening measure, may detect more cases than symptom/exposure screening. However, if only performed upon arrival, it may miss a significant proportion of cases. Effectiveness of testing as a control measure may be determined to a large extent by its sensitivity and turn‐around time. Early initiation, larger coverage, and integration with other public health and social measures (PHSM) may enhance effectiveness of screening and contact tracing
Response measures	Effectiveness of quarantine seems to depend on level of adherence to, duration and enforcement of measure Effectiveness of quarantine may increase when implemented along with other PHSM such as isolation, contact tracing, and travel ban. Unless the level of contact tracing and screening is high, prevention through isolation only may be very limited Early testing, symptom screening, and isolation of symptomatic cases may increase effectiveness of testing and isolation strategies
Service measures	Effectiveness of service measures in school settings may be influenced by level of community transmission, susceptibility of target populations, type of schooling (i.e., primary vs. secondary), other PHSM, timing of execution, and contextual factors (e.g., children's age) Implementing compensatory measures (e.g., summer learning groups) may counterbalance learning losses during school closure periods Workplace closures may be more effective during the initial stages of the pandemic, especially when the cumulative incidence is low Effectiveness of service measures may be contingent on adherence levels and the timing of implementation Incentive policies (e.g., economic assistance to people of society) may enhance adherence to measures
Social interactions	Home confinement effectiveness may be influenced by its initiation time, the strictness of measures, and duration Prompt deployment of social distancing strategies may be crucial, especially during the outbreak's early stages Population demographics, mobility rates, testing availability, climate, and population density may play roles in the effectiveness of distancing measures A robust health data infrastructure may be crucial for evaluating the efficacy of distancing measures
Personal protection measures	Appropriate and safe use of suitable masks as well as proper storage and cleaning or disposal of masks may enhance effectiveness of masking Supplementing use of masks by a comprehensive set of PHSM may enhance its overall effectiveness
Physical environment measures	Combining environmental measures may enhance their overall effectiveness Availability and types of resources (e.g., disinfectants, PPE, air purifiers, or ventilation systems) may play roles in the effectiveness of these measures Effectiveness of environment measures may increase when integrated with other PHSM
Movement measures	Effects of travel measures may depend on factors such as: design and stringency (e.g., single polymerase chain reaction (PCR) testing vs. repeated PCR testing; 7‐day quarantine vs. 14‐day quarantine); levels of community transmission; travel volumes and duration; other public health measures in place; the exact specification and timing of the measure; degree of adherence; and enforcement of measures (e.g., recommendation to quarantine, various forms of control, fine and magnitude of fine) It may be critical to start early, have a wide coverage, and integrate these with other PHSM
Lockdown	Duration of lockdown may be determined by considering the effective reproduction number, daily new confirmed cases, daily death cases, and the public adherence of other preventive measures. Relaxing or lifting lockdown too early may lead to a second pandemic wave and delay the epidemic peak. Executing lockdown as early as possible may be essential to contain the transmission and avoid nationwide outbreaks. Compared to only lockdown, the addition of mass testing to lockdown may enhance effectiveness.

## 4 DISCUSSION

### 4.1 Summary and interpretation of main results

Our overview of systematic reviews identified 94 eligible reviews examining the effectiveness and unintended health and socioeconomic consequences of PHSM during the COVID‐19 pandemic. Of these, 24 (26%) reviews included meta‐analyses and 16 (17%) applied the GRADE framework.

The included reviews identified very few RCTs (*n* = 8), the most robust design for causal inferences. Only six (6%) of the reviews found at least one RCT, and only one reported results separately for RCTs.

The most frequently examined PHSM category was personal protection (*n* = 18, 19%), and the least examined PHSM category was physical environment (*n* = 3, 3%). Within multicomponent interventions, lockdown was the most frequently examined category despite lacking clarity of what the term entails (*n* = 39, 41%). The most frequently reported outcome category was non‐COVID‐19‐related health outcomes (*n* = 58, 62%), with mental health outcomes being the most frequently reported among those. COVID‐19 epidemiological outcomes were reported in 40 (43%) of the reviews. Only five (5%) of the reviews reported on socioeconomic outcomes, specifically educational attainment, absenteeism, and disability. This points to the difficulty of conducting interdisciplinary primary studies measuring outcomes across sectors.

Findings suggest that PHSM, in particular multicomponent interventions, may be effective incontrolling the risk and scale of COVID‐19 transmission (mainly low‐ to very low‐certainty evidence). Findings also suggest unintended health and socioeconomic consequences associated with PHSM (mainly very low‐certainty evidence). While no unintended consequences were reported for active surveillance and measures restricting movement, the remaining measures may be associated with a range of non‐COVID‐19 related health outcomes, including increased substance use, physical inactivity, mental health disorders, poor nutrition, and prolonged screen time. Personal protection may also be associated with negative physiological effects (e.g., increased heart rate, perceived exertion, dyspnea, hyperhidrosis, headache, eczema, and dermatitis). Furthermore, multicomponent PHSM may be associated with changes in the pattern of health care service utilization, particularly with decreases in visits to the emergency department, referrals for medical examinations associated with child protection, routine immunizations, and cancer screening. Additional unintended health effects may include delayed diagnoses and increased avoidable deaths from cancer. Service measures may be associated with negative socioeconomic consequences for students' achievement, the number of days of school missed and children's loss of access to school‐based health care and social services. Such measures may also be associated with absence from work among health care workers.

Confidence in most of the reviews was rated as low to very low, based on AMSTAR 2, with only four reviews rated as high and four as moderate. The available evidence on unintended effects is also predominantly from reviews that had low to very low AMSTAR 2 ratings.

### 4.2 Meta‐findings across the reviews

Four patterns are worth noting for this overview of systematic reviews. First, although a substantial number of reviews were identified, the majority were rated as low or very low confidence on the AMSTAR 2 instrument. An earlier overview of systematic reviews that analysed evidence from the first 18 systematic reviews published after the emergence of COVID‐19 concluded that the confidence in the results of all reviews was “critically low” [10]. Our findings indicate that systematic reviews published later during the pandemic remained of poor methodological quality. Second, while almost all of the included reviews suggested positive associations between PHSM and COVID‐19 epidemiological outcomes across settings and areas, several of the PHSM were also associated with unintended health, social, and economic consequences. Third, while the objective was to address the effectiveness of PHSM, most of the reviews included primary studies other than RCTs, allowing us to make only inferences about associations between the interventions and outcomes of interest. Fourth, although we intentionally sought a broad range of PHSM and outcome measures, a substantial number of reviews identified had a fairly narrow scope, specifically examining the association between the not clearly specified multicomponent intervention “lockdown” and non‐COVID‐19‐related health outcomes, particularly mental health outcomes. A similar pattern was reported by Chiesa et al. [109]. This may indicate a need to reduce duplication across reviews and invest in funding reviews across disciplines. Additionally, socioeconomic consequences may take much longer to become apparent and thus may have not been captured within the timeframe of our review.

### 4.3 Comparisons with other overviews

Two related overviews have been published since the inception of our overview [12, 13]. One focused on the effects of nonpharmacological interventions (NPIs) on the transmission of severe acute respiratory syndrome coronavirus 2 (SARS‐CoV‐2) infection, but the review did not explore indirect, social, or economic impacts [12]. It was not clear how this review synthesized data or how it considered methodological limitations when interpreting findings. The review identified the following NPIs as having the clearest evidence of positive impact: social distancing, hygiene measures, masking, and testing policies. The reviewers also concluded that combined interventions are effective in reducing the transmissibility of COVID‐19.

Another recent report summarized the findings from six commissioned evidence reviews examining six individual NPIs [110]. The report acknowledged the overall weakness of the evidence base on the effectiveness of PHSM and COVID‐19. Nonetheless, the authors conclude there are clear signals that many of the NPIs were effective, especially when implemented in combination.

Our overview examined both effectiveness and unintended health and socioeconomic effects of PHSM. Similar to the other reviews, our findings suggest that combining measures may be more effective than implementing individual measures in improving COVID‐19 epidemiological outcomes.

### 4.4 Strengths and limitations

To our knowledge, this is the first overview of systematic reviews to comprehensively identify, critically appraise, and synthesize published reviews assessing the effectiveness and unintended effects of PHSM in the context of the COVID‐19 pandemic. Our overview is methodologically robust, particularly that we appraised the quality of all included reviews and reported GRADE assessments where available. In addition, the overview used a comprehensive literature search without language restrictions. The included reviews comprise both experimental and observational studies. To further enhance the rigor of the overview, only reviews with a systematic search strategy and a clear description of the risk of bias assessment were included. Additionally, the overview was also built on the PHSM conceptual framework developed by Rehfuess et al. in collaboration with WHO [18, 110].

Reviews assessing responses to other health emergencies (e.g., H1N1, influenza) were excluded, as were reviews that included COVID‐19 alongside other outbreaks. While this was intended to avoid unnecessary heterogeneity, it meant that indirect but potentially informative evidence was excluded. Additionally, given that it may take some time for primary studies to be published and be included in a review then, this overview may consequently cover “short term effects” only.

### 4.5 Implications for policy and practice

Low certainty evidence suggests that multicomponent interventions may be the most effective in controlling the risk and scale of COVID‐19 transmission. Most of the multicomponent interventions included a combination of active surveillance and response measures, social measures, and personal protective measures (specifically face masking and hand hygiene). There is, however, uncertainty around the effectiveness of those measures in school settings. In terms of single measures, the findings suggest that active surveillance and social interaction measures may be effective on their own while personal protection measures alone may not provide sufficient protection from COVID‐19 outbreaks. For travel‐related measures and outcomes, the certainty of the evidence is very low and the true effects are likely to be substantially different from those reported in the reviews.

To optimize the effects of PHSM interventions, policymakers should consider what might be optimal implementation timing for PHSM (e.g., when community transmission or transmission intensity is still low). Table 5 provides an overview of tentative factors that may influence the effectiveness of interventions, including the level of integration with other PHSM, the susceptibility of target populations, and the level of adherence to and enforcement of the measure.

While PHSM may be effective in improving COVID‐19‐related epidemiological outcomes, they may also have unintended health (e.g., mental health) and socioeconomic consequences (e.g., disrupted education). Thus, decision‐makers need to find an adequate balance between effectiveness and unintended consequences. The implementation and scaling up of mitigation measures, such as social protection policies and programs and community‐led initiatives, can reduce the socioeconomic burden of PHSM. In addition, implementation strategies should consider contextual factors, including values, resource use, equity implications, acceptability, and feasibility [111].

Stakeholders, including community members, should be fully consulted and involved in PHSM decision‐making to elicit their inputs on contextual considerations. Such engagement is important to ensure measures are context‐specific and acceptable and to promote uptake and adherence [112]. Engagement can also help address political sensitivities related to PHSM decision‐making and should be sustained beyond the emergency period [113].

Investment in monitoring and evaluating PHSM is important considering the low certainty evidence available, and should be an integral part of efforts to strengthen health emergency preparedness and response activities at the national and subnational levels [114]. This also requires building capacity for collecting local data about PHSM policies.

### 4.6 Implications for research

The COVID‐19 pandemic created an unprecedented window of opportunity to strengthen the evidence ecosystem and prioritize evidence in decision‐making. At the same time, the pandemic revealed systemic weaknesses and failures in both domestic and global evidence infrastructures [115, 116] although it is important to also recognize that research during the COVID‐19 pandemic was conducted under high pressure in the context of rather new circumstance. There was a mismatch in expectations between evidence users and evidence producers: From the policymakers' side, there was a strong need for actionable research findings, while the researchers were rarely able to provide more than highly uncertain evidence [117]. This was further compounded by challenges pertaining to acquiring appropriate funding, establishing research networks to design feasible policy‐relevant studies, and catering to the logistics of running studies [117]. We discuss below implications for research at different levels.

At the level of primary studies, our findings highlight the need for more methodologically rigorous research that encompasses a range of study designs, including observational, experimental, and qualitative. While only a few RCTs have been conducted to date, they demonstrate that RCTs are feasible for PHSM [118, 119]. Where randomization is not feasible, the natural experiments created by some policies can be exploited, such as quasi‐arbitrary cut‐offs (e.g., re‐opening stores below number of square meters) [120]. Also, studies should be designed to capture the long‐term consequences of interventions, particularly given that socioeconomic consequences may take much longer to become apparent. At the level of evidence synthesis, future systematic reviews should follow best practices in terms of conduct and reporting. Where applicable, the GRADE and GRADE‐CERQual frameworks should be applied to assess the certainty of evidence for both quantitative and qualitative review findings [23, 121]. Another issue to address is the duplication of systematic reviews, leading to research waste [122, 123].

**Figure 5:**
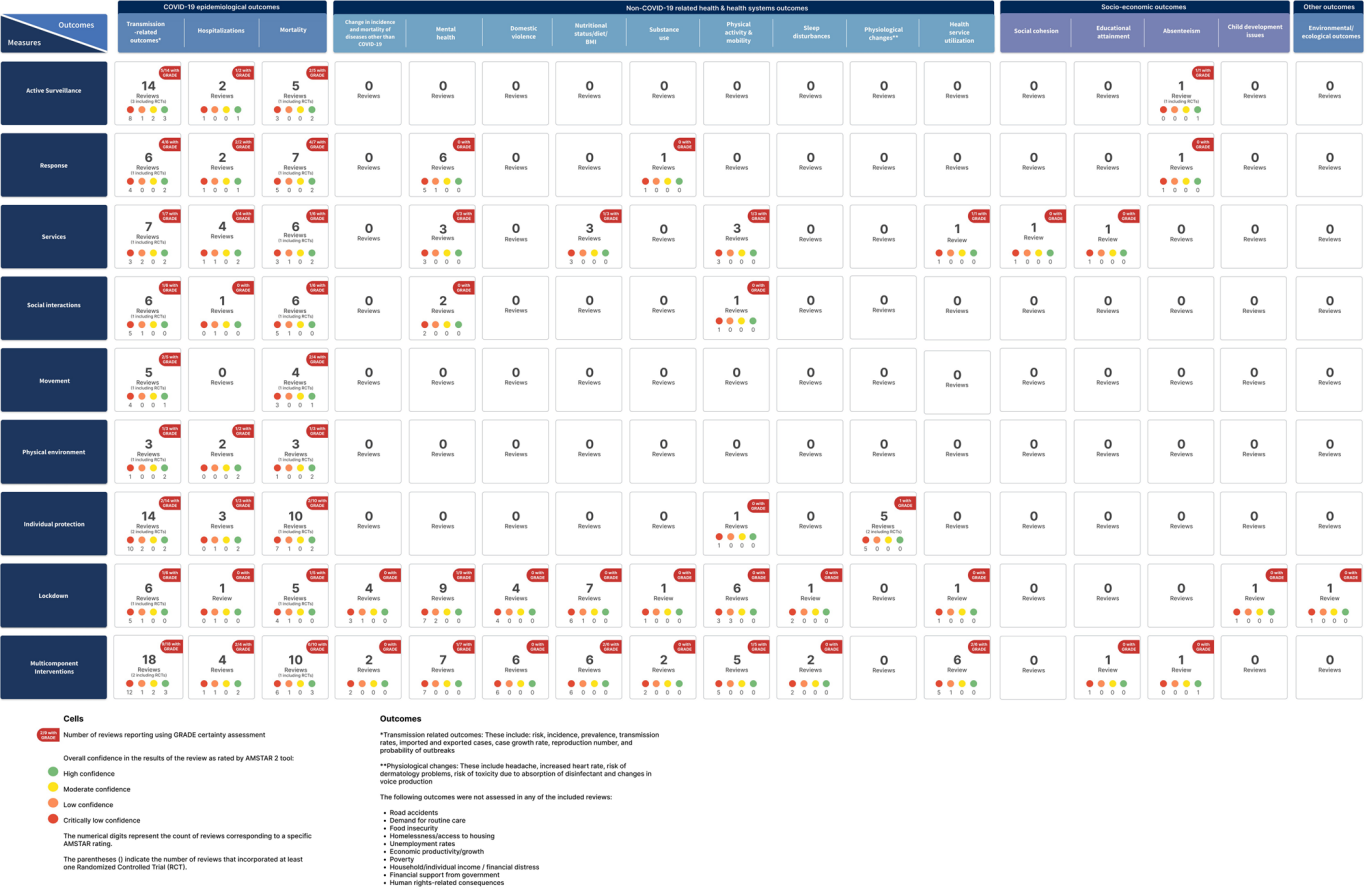
Map of evidence for and gaps in reviews of public health and social measures.

Importantly, there is a need to develop better interdisciplinary collaboration, methods and tools to address the highly relevant but less frequently researched systematic review questions revealed as gaps by our overview (Figure 5). For instance, when it comes to outcomes, the consequences of PHSM on the economy and on education and other sectors remain critically under‐researched.

Future overviews of reviews could complement our findings by exploring areas such as the contextual and effect modifiers of PHSM across settings; implementation considerations for different PHSM, including their feasibility, acceptability, cost implications, barriers and facilitators, and equity considerations; and interventions to alleviate the unintended health, social and economic consequences of PHSM.

Further efforts are required to standardize the definitions and operationalization of PHSM (e.g., isolation, quarantine, lockdown). The revised conceptual framework for the categorization of PHSM developed by WHO can serve as a step forward in that direction [18].

Moving forward, we need to leverage lessons learned from the COVID‐19 pandemic to mobilize evidence for better decision‐making against future crises. In particular, we need sustainable knowledge translation structures and processes to support national policy making [124]. It has become clear that capacities, institutional structures and infrastructure, and productive relations and partnerships between researchers, policymakers, and other stakeholders need ideally to be in place before a crisis hits [125]. Researchers, governments, and funders need to invest now in national research infrastructure and systems that promote the timely production of high‐quality evidence during a health emergency. This includes developing standard protocols, processes, and tools for facilitating evidence use in decision‐making; setting up and funding multidisciplinary research networks to enable multidisciplinary responses; establishing mechanisms to bridge the different entities nationally and internationally producing evidence for more effective, efficient, and timely responses; and ensuring appropriate communication to the public of evidence‐informed decisions [113]. The timeliness of decision‐making during a health emergency has also generated interest in rapid response services and living systematic reviews [7, 126]. Institutionalizing these processes and grounding them in existing and emerging methodologies can facilitate rapid responses during an emergency [127].

## 5 CONCLUSIONS

This overview of systematic reviews found predominantly low‐certainty evidence suggesting that PHSM, in particular multicomponent interventions, may be effective in controlling the risk and scale of COVID‐19 transmission. Additionally, very low‐certainty evidence suggested that the implementation of PHSM may have unintended health and social consequences. To develop a more reliable and comprehensive evidence base to enable the generation of recommendations for policy and practice, systematic reviews should follow reporting guidelines, and primary studies need to utilize more robust research designs. Importantly, better coordination and coproduction processses are needed to fill existing research gaps, avoid research waste, and align research and evidence syntheses with the priorities of policy‐makers and stakeholders.

## AUTHOR CONTRIBUTIONS

**Racha Fadlallah**: Conceptualization; data curation; formal analysis; methodology; project administration; supervision; validation; writing—original draft; writing—review and editing. **Fadi El‐Jardali**: Conceptualization; formal analysis; funding acquisition; investigation; methodology; resources; supervision; writing—review and editing. **Lama B. Karroum**: Data curation; formal analysis; investigation; methodology; software; writing—original draft. **Nour Kalach**: Data curation; Formal analysis; Investigation; Methodology; Writing—original draft. **Reem Hoteit**: Formal analysis; methodology; writing—original draft. **Andrew Aoun**: Data curation; formal analysis; writing—original draft. **Lara Al‐Hakim**: Data curation; formal analysis; writing—original draft. **Francisca Verdugo‐Paiva**: Data curation; methodology; software; visualization; writing—review and editing. **Gabriel Rada**: Methodology; resources; software; supervision; visualization; writing—review and editing. **Atle Fretheim**: Conceptualization; funding acquisition; methodology; resources; supervision; writing—review and editing. **Simon Lewin**: Conceptualization; funding acquisition; methodology; resources; supervision; writing—review and editing. **Ramona Ludolph**: Conceptualization; methodology; supervision; validation; writing—review and editing. **Elie A Akl**: Conceptualization; formal analysis; funding acquisition; investigation; methodology; resources; supervision; validation; writing—original draft; writing—review and editing.

## CONFLICT OF INTEREST STATEMENT

Three of the overview's authors (E. A. A., L. B. K., and G. R.) are coauthors of three of the included reviews. In line with Cochrane guidance, and to minimize the risk of bias, these authors were not involved in any data extraction or quality assessments of reviews.

## PEER REVIEW

The peer review history for this article is available at https://www.webofscience.com/api/gateway/wos/peer-review/10.1002/cesm.12055.

## ETHICS STATEMENT

The authors have nothing to report.

## Supporting information


**Appendix 1**: Detailed search strategy.

**Appendix 2**: Characteristics of each included review.

**Appendix 3**: Methodological assessment of included reviews.

**Appendix 4**: Detailed findings from all reviews stratified by intervention and outcome categories.

## Data Availability

The data that supports the findings of this study are available in the supplementary material of this article.
