# Supplementary material for: The effects of public health and social measures (PHSM) implemented during the COVID‐19 pandemic: An overview of systematic reviews
Source: Cochrane Evid Synth Methods. 2024 Apr 29;2(5):e12055. doi: 10.1002/cesm.12055 (PMC11795948; doi:10.1002/cesm.12055)
Supplement: Supplementary file 1 — Appendix 1: Detailed search strategy. [file CESM-2-e12055-s003.pdf]

Appendix 1: Detailed search strategy

The COVID-19 L·OVE repository was built, and is maintained, by systematic searches in multiple databases, trial registries and preprint servers. Searches are not restricted by study design, language or publication status:

The following sources are regularly searched:

- Pubmed/medline (updated several times a day)
- EMBASE (updated weekly)
- CINAHL (updated weekly)
- PsycINFO (updated weekly)
- LILACS (Latin American & Caribbean Health Sciences Literature) (updated weekly)
- Wanfang Database (updated every 2 weeks)
- CBM - Chinese Biomedical Literature Database (updated every 2 weeks)
- CNKI - Chinese National Knowledge Infrastructure (updated every 2 weeks)
- VIP - Chinese Scientific Journal Database (updated every 2 weeks)
- IRIS (WHO Institutional Repository for Information Sharing) (updated weekly)
- IRIS PAHO (PAHO Institutional Repository for Information Sharing)) (updated weekly)
- IBECS - Índice Bibliográfico Español en Ciencias de la Salud (Spanish Bibliographic Index on Health Sciences) (updated weekly)
- Microsoft Academic (last searched: 23 August 2021)
- ICTRP Search Portal (updated daily)
- Clinicaltrials.gov (updated daily)
- ISRCTN registry (updated daily)
- Chinese Clinical Trial Registry (updated daily)
- IRCT - Iranian Registry of Clinical Trials (updated daily)
- EU Clinical Trials Register: Clinical trials for covid-19 (updated daily)
- NIPH Clinical Trials Search (Japan) - Japan Primary Registries Network (JPRN) (JapicCTI, JMACCT CTR, jRCT, UMIN CTR) (updated daily, via ICTRP search portal)
- UMIN-CTR - UMIN Clinical Trials Registry (updated daily, via ICTRP search portal)
- JRCT - Japan Registry of Clinical Trials (updated daily, via ICTRP search portal)
- JAPIC Clinical Trials Information (updated daily, via ICTRP search portal)
- Clinical Research Information Service (CRiS), Republic of Korea (updated daily, via ICTRP search portal)
- ANZCTR - Australian New Zealand Clinical Trials Registry (updated daily, via ICTRP search portal)
- ReBec - Brazilian Clinical Trials Registry (updated daily, via ICTRP search portal)
- CTRI - Clinical Trials Registry - India (updated daily, via ICTRP search portal)
- RPCEC - Cuban Public Registry of Clinical Trials (updated daily, via ICTRP search portal)
- DRKS - German Clinical Trials Register (updated daily, via ICTRP search portal)
- LBCTR - Lebanese Clinical Trials Registry (updated daily, via ICTRP search portal)
- TCTR - Thai Clinical Trials Registry (updated daily, via ICTRP search portal)
- NTR - The Netherlands National Trial Register (updated daily, via ICTRP search portal)
- PACTR - Pan African Clinical Trial Registry (updated daily, via ICTRP search portal)
- REPEC - Peruvian Clinical Trial Registry (updated daily, via ICTRP search portal)
- SLCTR - Sri Lanka Clinical Trials Registry (updated daily, via ICTRP search portal)

- medRxiv (updated several times a day)
- bioRxiv (updated several times a day)
- SSRN Preprints (updated several times a day)
- ChinaXiv (updated every 2 weeks)
- SciELO Preprints (updated weekly)
- Research Square (updated daily)

Search strategy

	Search term	Search strategy
#1	**Public health social measures**	((suppress* OR contain* OR mitigat* OR "control disease") AND (transmission OR spread OR measure* OR strateg*)) OR ((preventive* OR nonpharmaceutical* OR "non-pharmaceutical" OR "non pharmaceutical" OR nonpharmacological* OR "non-pharmacological" OR "non pharmacological" OR nonpharmacologic* OR "non-pharmacologic" OR "non pharmacologic" OR nondrug* OR "non-drug" OR "non drug" OR "public health" OR restriction* OR containment* OR mitigation* OR government* OR societal*) AND (intervention* OR therap* OR treatment* OR approach* OR measure* OR strateg* OR policy OR policies OR management* OR method* OR modalit* OR option*)) OR ("social measure" OR "social measures" OR “social intervention” OR “social interventions” OR “social distancing” OR “social-distancing” OR “social distance” OR “social proximity” OR “social contact” OR npi OR npis OR phsm OR phsms)
#2	**hand hygiene and interventions to improve hand hygiene compliance**	(hand* AND (hygien* OR wash* OR rub* OR scrub*)) OR handwash* OR "hand-washing" OR handrub* OR "hand-hygiene"
#3	**hand cleaning with soap or alcohol-free instant hand sanitizers**	soap* OR ("alcohol-free" AND sanitizer) OR (hand* AND (sanitis* OR sanitiz*))
#4	**hand cleaning with ash**	ash OR ashes
#5	**outdoor disinfection**	(air OR spray OR environment* OR street* OR cities OR city OR outdoor* OR mass* OR sidewalk* OR park* OR plaza* OR "open-air" OR public) AND (sanitiz* OR sanitis* OR disinfect* OR decontamina* OR spray* OR cleaning*)
#6	**sanitizing tunnels and cabins**	(tunnel* OR gate OR cabin*) AND (sanit* OR disinfect* OR decontamina* OR spray* OR cleaning* OR hygienisat* OR hygienizat* OR cleanser*)
#7	**disinfection of surfaces, objects and facilities**	(objects OR inanimate* OR surface* OR facility OR facilities*) AND (sanitiz* OR sanitis* OR disinfect* OR decontamina* OR spray* OR cleaning* OR sanitary*)
#8	**air cleaning and humidification**	((air OR HEPA) AND (purif* OR filt* OR clean* OR ioni* OR saniti* OR humidifi* OR moisturi*))

#9	**public transport disinfection**	(transport* OR subway*) AND (sanitiz* OR sanitis* OR disinfect* OR decontamina* OR spray* OR cleaning*)
#10	**intubation box**	((intubation* OR enclosure* OR aerosol* OR barrier*) AND (box OR enclosure*)) OR (intubat* AND shield*)
#11	**social distancing measures**	(((spread* OR outbreak*) AND (((acute* OR upper* OR low* OR tract OR viral*) AND (respiratory*) AND (infection* OR disease* OR illness*)) OR (acute* AND bronchi*) OR ((virus* OR viral) AND respiratory*) OR alrti* OR lrti* OR alri* OR urti OR urtis* OR "influenza-like")) OR ((distancing AND measure*) OR (school* AND clos*)))
#12	**educational institution practices to promote social distancing**	(school* OR universit* OR preschool*) AND (outbreak OR pandemic* OR epidemic*)
#13	**educational institutions closures**	(school* OR "high-school" OR "high-schools" OR "k-12" OR "k12" OR k12* OR universit* OR preschool* OR (educational* AND (facilit* OR institution*))) AND (closure* OR closing* OR closed* OR lockdown* OR shut)
#14	**educational institutions reopening**	(reopen* OR "re-opening" OR opening OR "re-opened" OR "re-open" OR "re open" OR end* OR terminate* OR exit* OR lift*) AND (school* OR "high-school" OR "high-schools" OR "k-12" OR "k12" OR k12* OR universit* OR preschool* OR (educational* AND (facilit* OR institution*)))
#15	**teleeducation**	teleeducation* OR “tele-education” OR ((education* OR training* OR teach* OR studying OR learn* OR classroom*) AND (remote* OR home* OR online OR virtual OR distance OR distant OR internet OR videoconf*))
#16	**contact tracing**	(contact* AND (trace* OR tracing* OR track*)) OR "contact-tracing"
#17	**automated or semi-automated contact tracing**	(automat* OR digital* OR tech* OR electronic* OR app OR apps OR "app-based" OR application OR smartphone* OR "smart-phone" OR "smart-phones" OR mobile* OR phone* OR tele* OR online* OR internet* OR mhealth* OR "m-health" OR ehealth* OR "e-health" OR web* OR (text* AND messag*)) AND ((contact* AND (trace* OR tracing*)) OR "contact-tracing")
#18	**workplace practices to promote social distancing**	(workplace* OR business OR industry OR enterprise OR company) AND (outbreak OR pandemic* OR epidemic*)
#19	**workplace closures**	(workplace* OR business OR industry OR enterprise OR company) AND (closure* OR closing* OR closed* OR lockdown* OR shut)
#20	**telework**	telework* OR “tele-work” OR ((work* OR offic*) AND (remote* OR home* OR online OR virtual OR distance OR distant OR internet OR videoconf*))
#21	**travel-related measures to promote social distancing**	(travel* OR airport* OR port* OR harbor* OR landborder* OR border OR “ground crossings” OR flight OR visa) AND (outbreak OR pandemic* OR epidemic* OR "social distancing" OR “social measures” OR ban OR restriction OR control)
#22	**border closures**	border* AND (closure* OR closing* OR closed* OR lockdown* OR shut)

#23	**internal travel restrictions**	(internal* OR domestic* OR local OR communit*) AND (travel* OR movement* OR mobility*) AND (restriction*OR ban* OR control)
#24	**entry and exit screening**	(entry* OR exit* OR airport* OR port* OR harbor* OR landborder* OR border OR "ground crossings") AND (screening* OR supervision OR control)
#25	**travel advice to promote social distancing**	travel* AND advice*
#26	**airport screening**	airport* OR travel*
#27	**public transport closure**	transport* AND (closure* OR closing* OR cancel* OR suspension* OR suspending OR ban OR banning)
#28	**quarantine**	isolation* OR confinement* OR "community containment" OR "containment area" OR quarantine
#29	**reopening strategies and reopening-related interventions**	reopen* OR "re-opening" OR opening OR "re-opened" OR "re-open" OR "re open" OR ((end* OR terminate* OR exit* OR lift* OR leaving* OR withdrawal* OR ease* OR easing*) AND (lockdown* OR shelter* OR bans* OR restrictions* OR "stay-at-home")) OR "exit strategy" OR "exit strategies" OR deconfin*
#30	**personal measures to avoid physical contact (e.g. keeping distance of separation, avoiding direct physical contact)**	gesture* OR "hand-shake" OR handshak* OR ((keep* OR maintain*) AND (meter OR metre* OR meters OR foot OR feet OR "two-meter" OR "two-metre" OR "2-meter" OR "2-metre" OR "six-foot" OR "6-foot" OR "6 foot")) OR "physical distancing" OR "physical contact"
#31	**restricting mass gatherings and avoiding or closing crowded public spaces**	crowd* OR gathering* OR restaurant* OR movie* OR cinema* OR theat* OR shopping* OR mall OR club* OR temple* OR church* OR mosque* OR synagogue* OR (sport* AND (center* OR centre* OR events OR event)) OR ((restrict* OR ban OR cancel*) AND (mass* OR public* OR religio*) AND (event OR events)) OR "mass events" OR "mass event" OR "public event"
#32	**immunity passports**	(immunity* OR recover* OR release* OR vaccin*) AND (passport* OR certificat*)
#33	**government-mandated social distancing (so-called 'lockdown')**	(shield* AND vulnerable) OR shielding* OR cocooning* OR confinement* OR lockdown* OR "lock-down" OR shelter* OR "stay at home" OR "stay-at-home"
#34	**impact of curfews**	curfew*
#35	**tier system**	tier
#36	**interventions aimed at hospitality venues**	hospitality
#37	**Measures in response to a case, outbreak or rising number of cases in a**	(response* AND measure*)

	**setting or locality**	
#38	**respiratory etiquette**	((cough* OR respiratory*) AND etiquette*) OR ((cough* OR sneeze*) AND cover*)
#39	**personal protective equipment**	(protective* AND equipment*) OR PPE
#40	**gloves**	glov* OR "double-gloving" OR "double-glove" OR "double-gloves"
#41	**shoe covers**	shoe* OR overshoe* OR boot* OR galosh* OR dickersons* OR gumshoes* OR rubbers*
#42	**masks**	mask OR masks
#43	**facemasks**	facemask* OR "face-mask" OR "face-masks" OR N95* OR "n-95" OR "n 95" OR ((facial* OR face* OR respirator* OR wearing* OR "use of" OR protective*) AND mask*) OR (mask* AND surgical*)
#44	**cloth facemasks**	(facemask* OR ((facial* OR face* OR respirator*) AND (mask* OR surgical*))) AND (handcraft* OR "hand-craft" OR "hand craft" OR "hand-crafted" OR "hand crafted" OR handmade* OR "hand-made" OR "hand made" OR cloth* OR cotton* OR "non medical" OR "non-medical")
#45	**reuse of facemasks and respirators**	(facemask* OR "face-mask" OR "face-masks" OR N95* OR "n-95" OR "n 95" OR ((facial* OR face* OR respirator* OR wearing* OR "use of" OR protective*) AND mask*) OR (mask* AND surgical*)) AND ("re-use" OR reuse* OR reutili*)
#46	**N95 masks**	N95* OR "N-95" OR "N 95"
#47	**clear facemasks**	((clear OR transparen*) AND (facemask* OR mask*)) OR ClearMask*
#48	**face shields**	face* AND shield*
#49	**eye protection**	goggle* OR glasses* OR ((eye* OR conjunctiva*) AND protect*) OR sunglass*
#50	**head coverings**	(head* AND cover*) OR gown*
#51	**public space disinfection**	(((air OR spray OR environment* OR street* OR cities OR city OR outdoor* OR mass* OR sidewalk* OR park* OR plaza* OR "open-air" OR public) AND (sanitiz* OR sanitis* OR disinfect* OR decontamina* OR spray* OR cleaning*)) OR ((tunnel* OR gate OR cabin*) AND (sanit* OR disinfect* OR decontamina* OR spray* OR cleaning* OR hygienisat* OR hygienizat* OR cleanser*)) OR ((transport* OR subway*) AND (sanitiz* OR sanitis* OR disinfect* OR decontamina* OR spray* OR cleaning*)))
#52	**national pandemic strategies**	Zero Covid OR "Zero-Covid" OR ZeroCovid* OR (curve* AND flatt*) OR “flatten-the-curve”
#53	**interventions to handle the bodies of deceased persons**	cadaver* OR corpse* OR carcass* OR mortem* OR mortu* OR postmort* OR "post-mortem" OR cremat* OR tomb OR tombs OR buri* OR bury* OR entomb* OR sepultur* OR forens* OR autops* OR ((body* OR bodies* OR dead) AND (dispos* OR handl* OR deceased* OR (pass* AND away))) OR (human AND remains)

**#54**	**non-contact thermometers**	(temperature* OR fever* OR thermom* OR thermal*) AND (screen* OR airport* OR port* OR harbor* OR landborder* OR "ground crossings" OR flight OR sensor* OR infrared* OR non-contact OR gun OR laser* OR smart* OR scanner* OR imager*)
**#55**	**surveillance measures test or screen individuals and/or groups of people**	(surveillance AND measure*) OR ((surveillance OR routine OR intensive OR asymptomatic* OR community OR outbreak OR mass) AND (screening OR testing)) OR "mass-testing"
**#56**	**interventions to improve ventilation and air cleaner/purifier**	(indoor* OR home* OR classroom* OR residential* OR room* OR portable*) AND air AND (quality* OR purif* OR clean* OR filter* OR filtration*)
**#57**		#1 OR #2 OR #3 OR #4 OR #5 OR #6 OR #7 OR #8 OR #9 OR #10 OR #11 OR #12 OR #13 OR #14 OR #15 OR #16 OR #17 OR #18 OR #19 OR #20 OR #21 OR #22 OR #23 OR #24 OR #25 OR #26 OR #27 OR #28 OR #29 OR #30 OR #31 OR #32 OR #33 OR #34 OR #35 OR #36 OR #37 OR #38 OR #39 OR #40 OR #41 OR #42 OR #43 OR #44 OR #45 OR #46 OR #47 OR #48 OR #49 OR #50 OR #51 OR #52 OR #53 OR #54 OR #55 OR #56
