# Supplementary material for: The effects of public health and social measures (PHSM) implemented during the COVID‐19 pandemic: An overview of systematic reviews
Source: Cochrane Evid Synth Methods. 2024 Apr 29;2(5):e12055. doi: 10.1002/cesm.12055 (PMC11795948; doi:10.1002/cesm.12055)
Supplement: Supplementary file 2 — Appendix 2: Characteristics of each included review. [file CESM-2-e12055-s004.pdf]

Appendix 2: Characteristics of Included Reviews

Review	Total Number of studies included	Study Design	Geographical Region (WHO Classification)	Setting	Target population	Intervention	Outcome
(Asín-Izquierdo, Ruiz-Ranz, & Arévalo-Baeza, 2022)	8	Randomized Controlled Trial, Cross-sectional, Repeated measures design	NA	Healthcare settings, occupational setting	Adults	• Individual protection (Use of Face mask)	• Unintended health, social and economic outcomes (Physiological outcomes)
(Della Valle et al., 2021)	12	Longitudinal study, Cross-sectional study	European Region, Eastern Mediterranean Region	Not specified	General population	• Lockdown	• Unintended health, social and economic outcomes (Nutrition)
(Alex et al., 2021)	18	Not reported	Region of the Americas, European Region, Western Pacific Region, South-East Asian Region	Not specified	General population	• Multicomponent interventions (Social interactions and lockdown)	• Unintended health, social and economic outcomes (Violence)
(Alkatout et al., 2021)	10	Cohort Study, Longitudinal study, Cross-sectional study, Modeling Studies	Region of the Americas, European Region, Western Pacific Region	Not specified	General population	• Lockdown	• Unintended health, social and economic outcomes (Change in incidence and mortality of diseases other than COVID-19)
(Antonio, Chiara, Ilaria, Matteo, & Edoardo, 2021)	36	Not reported	Eastern Mediterranean Region (EMR), South-East Asian Region, Region of the Americas, Western Pacific Region, European Region	Not specified	People with pre-existing conditions	• Lockdown	• Unintended health, social and economic outcomes (Change in incidence and mortality of diseases other than COVID-19)
(Kunstler et al., 2022)	21	Randomized Controlled Trial, Time Series, Case Control, Cohort Study, Longitudinal study, Cross-sectional study	Region of the Americas, South-East Asian Region, European Region	Healthcare facility	Healthcare workers	• Individual protection (P2/N95 (or equivalent) respirators and surgical masks)	• COVID-19 epidemiological outcomes (Risk and incidence)<br>• Unintended health, social and economic outcomes (Physiological outcomes)

(Bakaloudi, Jeyakumar, Jayawardena, & Chourdakis, 2021)	32	Cross-sectional study	Eastern Mediterranean Region (EMR), South-East Asian Region, Region of the Americas, Western Pacific Region, European Region	Not specified	Adolescent, adults	• Lockdown	• Unintended health, social and economic outcomes (Nutrition)
(Baumhardt et al., 2021)	27	Not reported	European Region, Region of the Americas	Healthcare facility	People with pre-existing conditions	• Lockdown	• Unintended health, social and economic outcomes (Change in incidence and mortality of diseases other than COVID-19)
(Bou-Karroum et al., 2021)	69	Modeling study, Observational study	Eastern Mediterranean Region (EMR), Region of the Americas, European Region, Western Pacific Region, South-East Asian Region, African Region	Not specified	General population	• Multicomponent interventions (Movement and social interactions; services; Response; Surveillance)	• COVID-19 epidemiological outcomes (Risk and incidence, Transmission related outcomes, Mortality)
(Brakspear, Boules, Nicholls, & Burmester, 2022)	16	Cross-sectional study, Cohort Study	European Region, Western Pacific Region, South-East Asian Region, Eastern Mediterranean Region	Not specified	Children/Adolescent	• Lockdown	• Unintended health, social and economic outcomes (Nutrition)
(Mignogna et al., 2021)	95	Cohort Study, Longitudinal study, Cross-sectional study	African Region, Region of the Americas, South-East Asian Region, European Region, Eastern Mediterranean Region, Western Pacific Region	Not specified	Adults and children	• Lockdown	• Unintended health, social and economic outcomes (Nutrition)

(Camacho-Montaño et al., 2022)	8	Longitudinal study, Cross-sectional study, Observational study	Eastern Mediterranean Region (EMR), South-East Asian Region, Region of the Americas, Western Pacific Region, European Region	Household setting	Children	• Lockdown	• Unintended health, social and economic outcomes (Sleep)
(Cardwell et al., 2021)	3	Modeling Studies, Case series	Region of the Americas, Western Pacific Region, European Region	Point of entry (incl airport, ports, land borders)	General population	• Active Surveillance (Non-contact thermal screening)	• COVID-19 epidemiological outcomes (Transmission-related outcomes)
(Caristia et al., 2020)	19	Quasi-randomized Controlled Trial, Time Series, Modeling Studies, Case series	Western Pacific Region, European Region, Region of the Americas, Eastern Mediterranean Region	Not specified	General population	• Lockdown	• COVID-19 epidemiological outcomes (Risk and incidence, Transmission-related outcomes)
(Cavicchioli et al., 2021)	21	Cross-sectional study	European Region, Western Pacific Region, Eastern Mediterranean Region	Not specified	General population	• Response (Quarantine)	• Unintended health, social and economic outcomes (Mental Health)
(Chaabane, Doraiswamy, Chaabna, Mamtani, & Cheema, 2021)	10	Time Series, Cross-sectional study, Modeling Studies, Qualitative study	European Region, South-East Asian Region, Region of the Americas, Western Pacific Region	Educational institutions	Children/Adolescent	• Services (School Closure)<br>• Multicomponent interventions (Services (school closure) combined with Lockdown and Response)	• Unintended health, social and economic outcomes (Mental Health, Physical Activity and/or Nutrition, Health service utilization patterns, educational attainment, Healthcare Utilization)
(Chai et al., 2021)	12	Cross-sectional study	Western Pacific Region	Not specified	Children/Adolescent	• Response (Quarantine)	• Unintended health, social and economic outcomes (Mental Health)
(Bakaloudi, Barazzoni, et al., 2021)	36	Cohort Study, Cross-sectional study	African Region, Region of the Americas, South-East Asian Region, European Region, Eastern Mediterranean Region	Not specified	Adults and adolescents	• Lockdown	• Unintended health, social and economic outcomes (Nutrition)

(Daniela et al., 2020)	7	Case Control, Cohort Study, Cross-sectional study	Region of the Americas, Western Pacific Region	Household setting; community settings	General population	• Individual protection (Use of Face mask)	• COVID-19 epidemiological outcomes (Transmission-related outcomes)
(Desye, 2021)	28	Not reported	More than one Region	Not specified	Not specified	• Individual protection (Hand washing with soaps; Hand sanitizer with alcohol)	• COVID-19 epidemiological outcomes (Transmission-related outcomes)<br>• Unintended health, social and economic outcomes (Physiological outcomes)
(Elisabeth, Karlen, & Magkos, 2021)	27	Not reported	Western Pacific Region, European Region, Region of the Americas, Eastern Mediterranean Region, African Region	Not specified	General adult population or elderly.	• Lockdown	• Unintended health, social and economic outcomes (Nutrition, Physical activity)
(Farooq, Tunmore, Ali, & Ayub, 2021)	38	Case Control, Cohort Study, Longitudinal study, Cross-sectional study, Qualitative, case reports, Mixed Methods	European Region, region of the Americas, East Asia regions, Eastern Mediterranean Region, Western Pacific Region, South-East Asian Region	Not specified	General population	• Multicomponent interventions (Response, Social interactions)<br>• Lockdown	• Unintended health, social and economic outcomes (Mental Health)
(Freiberg, Schubert, Romero Starke, Hegewald, & Seidler, 2021)	33	Cohort Study, Cross-sectional study, Prospective secondary data analysis	Region of the Americas, European Region, South-East Asian Region, Western Pacific Region	Educational institutions, Healthcare facility, Occupational setting, Entertainment facilities	General population	• Services (School closures)<br>• Lockdown	• Unintended health, social and economic outcomes (Nutrition, Physical activity)
(Garofolo et al., 2021)	17	Observational study	European Region	Not specified	People with pre-existing conditions	• Lockdown	• Unintended health, social and economic outcomes (Change in incidence and mortality of diseases other than COVID-19)
(Girum, Lentiro, Geremew, Migora, & Shewamare, 2020)	22	Case Control, Cross-sectional study, Modeling Studies, Case study	Region of the Americas, Western Pacific Region, European Region	Healthcare settings, Community Settings, and national level	Individuals who had contact with confirmed or suspected cases of COVID-19, who travelled from countries with a declared outbreak, or who live in regions with high disease transmission	• Response (Quarantine)<br>• Multicomponent interventions (Response and surveillance)<br>• Lockdown	• COVID-19 epidemiological outcomes (Risk and incidence, mortality, Transmission-related outcomes, hospitalization)

(Grekousis & Liu, 2021)	19	Not reported	Western Pacific Region, European Region, Region of the Americas	Not specified	Not specified	• Active surveillance (Digital contact tracing)	• COVID-19 epidemiological outcomes (Transmission-related outcomes)
(Grépin et al., 2021)	29	Modeling Studies, Observational study	Eastern Mediterranean Region (EMR), South-East Asian Region, Region of the Americas, Western Pacific Region, European Region, African Region	Point of entry (incl airport, ports, land borders)	General population	• Movement (Travel-related measures (applied either at an international or domestic border))	• COVID-19 epidemiological outcomes (Transmission-related outcomes)
(Hammerstein, König, Dreisörner, & Frey, 2021)	11	Not reported	Region of the Americas, Western Pacific Region, European Region	Educational institutions	Children/Adolescent	• Services (School closures)	• Unintended health, social and economic outcomes (Educational attainment)
(Hatami et al., 2022)	18	Cohort Study, Cross-sectional study, Modeling Studies, Case series	Region of the Americas, European Region, Western Pacific Region, South-East Asian Region	Not specified	General population	• Multicomponent interventions (Surveillance, Response, Lockdown)	• COVID-19 epidemiological outcomes (Transmission-related outcomes)
(Hossain et al., 2022)	47	Quasi-randomized Controlled Trial, controlled before and after study, Case Control, Cohort Study, Cross-sectional study	Western Pacific Region, European Region, Region of the Americas, South-East Asian Region	Rural, urban, national, national, neighborhoods, districts, communities, households, provinces, catchment areas of PHCs	Not specified	• Active Surveillance (Wide range of tracing strategies)	• COVID-19 epidemiological outcomes (Risk and incidence, Transmission-related outcomes)
(Hugelius, Harada, & Marutani, 2021)	17	Not reported	Eastern Mediterranean Region (EMR), Region of the Americas, Western Pacific Region, European Region, South-East Asian Region	Nursing homes, Healthcare facilities	Not specified	• Services (Measures reducing the opportunity for contacts)	• COVID-19 epidemiological outcomes (Transmission-related outcomes)<br>• Unintended health, social and economic outcomes (Mental Health, Physical Activity and/or Nutrition, Social cohesion)

(Iezadi et al., 2021)	35	Quasi-randomized Controlled Trial, Time Series, Case Control, Cohort Study, Cross-sectional study, Ecological study; Observational study	Eastern Mediterranean Region (EMR), South-East Asian Region, Region of the Americas, Western Pacific Region, European Region	Community setting	General population	• Services (Large events and school closures)<br>• Social interactions (Social Distancing measures), Individual protection (Mask wearing)<br>• Multicomponent interventions (Social distancing measures; Restriction on domestic mobility; response; Combination of NPHIs)<br>• Lockdown	• COVID-19 epidemiological outcomes (Transmission-related outcomes, Mortality, Hospitalization, Risk and incidence)
(Ayouni et al., 2021)	18	Quasi-randomized Controlled Trial, Time Series, Cohort Study, Longitudinal study	Eastern Mediterranean Region (EMR), South-East Asian Region, Region of the Americas, Western Pacific Region, European Region, African Region	Not specified	Not specified	• Response (Isolation, quarantine of close contacts and home quarantine)<br>• Social interactions (Physical Distancing)<br>• Individual protection (Mask wearing)<br>• Multicomponent interventions (Movement and Response; Lockdown, social interactions, and movement; Lockdown; Surveillance and Response; Services and social interactions; Individual protection and movement)	• COVID-19 epidemiological outcomes (Transmission-related outcomes)
(Ingram et al., 2021)	33	Case Control, Cohort Study, Cross-sectional study, Modeling Studies, Observational study	Western Pacific Region, European Region, Region of the Americas	Healthcare settings, occupational setting	Not specified	• Active Surveillance (asymptomatic PCR testing)<br>• Individual protection (Universal masking policies)<br>• Individual protection (IPC measures (contact tracing and case isolation, PPE, and facility zoning))<br>• Multicomponent Interventions (Individual protection and surveillance)	• COVID-19 epidemiological outcomes (Risk and incidence, Transmission-related outcome)

(Burns et al., 2021)	62	Case Control, Cohort Study, Cross-sectional study, Modeling Studies, Observational study	African Region, Region of the Americas, South-East Asian Region, European Region, Eastern Mediterranean Region, Western Pacific Region	Point of entry (incl airport, ports, land borders)	General population	• Active Surveillance (symptom/exposure-based screening or test-based screening or both)<br>• Response (Quarantine)<br>• Movement (Travel restrictions reducing or stopping cross-border travel)<br>• Multicomponent Interventions (Response (Quarantine) and Surveillance)	• COVID-19 epidemiological outcomes (Risk and incidence, Transmission-related outcome, Mortality)
(Stratil et al., 2021)	22	Time Series, Case Control, Cohort Study, Modeling Studies	Region of the Americas, European Region	Nursing homes	Adults	• Active Surveillance (Routine testing; PCR Testing and antigen-based approaches)<br>• Response (Quarantine)<br>• Services (Measures reducing the opportunities for contacts (i.e., reducing contact between residents and among staff; self-confinement of staff with residents))<br>• Physical Environment (Cleaning and environmental hygiene measures)<br>• Individual protection (Face Mask; PPE usage)<br>• Multicomponent interventions (Surveillance and individual protection)	• COVID-19 epidemiological outcomes (Risk and incidence, Transmission-related outcomes, Mortality, hospitalization)
(Jabs et al., 2022)	39	Cohort Study, Cross-sectional study, Case series	African Region, Region of the Americas, South-East Asian Region, European Region, Eastern Mediterranean Region, Western Pacific Region	Healthcare facility	Healthcare workers	• Active Surveillance (PCR testing)	• COVID-19 epidemiological outcomes (Risk and incidence)

(Jenniskens et al., 2021)	17	Modeling Studies, Empirical	Western Pacific Region, European Region, Region of the Americas	Not specified	General population	• Active Surveillance (Contact tracing apps (CTAs))	• COVID-19 epidemiological outcomes (Risk and incidence, Transmission-related outcomes, Mortality)
(Castaldelli-Maia, Marziali, Lu, & Martins, 2021)	60	Not reported	Eastern Mediterranean Region (EMR), South-East Asian Region, Region of the Americas, Western Pacific Region, European Region, African Region	Not specified	General population	• Multicomponent interventions (Physical distancing measures)	• Unintended health, social and economic outcomes (Mental Health)
(Kharel et al., 2022)	71	Cohort Study, Cross-sectional study, Qualitative study	Eastern Mediterranean Region (EMR), Region of the Americas, European Region, Western Pacific Region, South-East Asian Region	Not specified	Children/Adolescent	• Lockdown	• Unintended health, social and economic outcomes (Physical Activity)
(Khatib et al., 2022)	3	Cohort Study	Western Pacific Region	Healthcare facility	Healthcare workers	• Individual protection (Handwashing)<br>• Multicomponent interventions (Lockdown, Response, Individual protection, social interactions)	• COVID-19 epidemiological outcomes (Risk and incidence)
(Khosravizadeh, Ahadinezhad, Maleki, Najafpour, & Golmohammadi, 2021)	13	Time Series, Observational study	Region of the Americas, South-East Asian Region, European Region, Western Pacific Region	Not specified	Adults	• Social interactions (Physical Distancing strategies)	• COVID-19 epidemiological outcomes (Risk and incidence)
(Kourti, Stavridou, Panagouli, Psaltopoulou, Spiliopoulou, et al., 2021)	32	Cohort Study, Longitudinal study, Cross-sectional study	Western Pacific Region, European Region, Region of the Americas, Eastern Mediterranean Region, African Region	Not specified	General population	• Lockdown	• Unintended health, social and economic outcomes (Violence)

(Kourti, Stavridou, Panagouli, Psaltopoulou, Tsolia, et al., 2021)	17	Cohort Study, Cross-sectional study	Region of the Americas, European Region	Not specified	Children/Adolescent	• Lockdown	• Unintended health, social and economic outcomes (Early child development)
(Fricke, Glöckner, Dreier, & Lange, 2020)	23	Not reported	Region of the Americas, South-East Asian Region, European Region	Not specified	General population	• Multicomponent interventions (Surveillance, response, services, social interactions, individual protection)	• Unintended health, social and economic outcomes (Change in incidence and mortality of diseases other than COVID-19)
(Lausi et al., 2021)	19	Cohort Study, Cross-sectional study	Region of the Americas, European Region, Western Pacific Region, South-East Asian Region, African Region	Not specified	General population	• Multicomponent interventions (Stay at home (SAH) policies)<br>• Lockdown	• Unintended health, social and economic outcomes (Violence)
(Bonati, Campi, & Segre, 2022)	105	Cohort Study, Modeling Studies	European Region	Community setting	General population	• Response (Quarantine)<br>• Lockdown	• Unintended health, social and economic outcomes (Mental Health)
(Mbwogge, 2021)	35	Cohort Study, Cross-sectional study, Modeling Studies	Region of the Americas, South-East Asian Region, European Region, Western Pacific Region	Community setting	General population	• Multicomponent interventions (Surveillance)	• COVID-19 epidemiological outcomes (Transmission related outcomes)
(Mendez-Brito, Bcheraoui, & Pozo-Martin, 2021)	34	Not reported	Western Pacific Region, Region of the Americas, European Region, African Region, South-East Asian Region, Eastern Mediterranean Region	Healthcare facility, educational setting, occupational setting, Point of entry, public, community setting	General population	• Services (School, workplace, business, and venue closing and public event bans)<br>• Social Interactions (Social Interaction measures)<br>• Movement (travel restrictions)<br>• Individual protection (Mask wearing)<br>• Lockdown	• COVID-19 epidemiological outcomes (Risk and incidence, Transmission-related outcome, Mortality)
(Minozzi, Saulle, Amato, & Davoli, 2021)	27	Uncontrolled before and after study, Cohort Study, Cross-sectional study	Region of the Americas, European Region, Western Pacific Region, South-East Asian Region	Not specified	Children/Adolescent	• Multicomponent (Social distancing measures (school closures and lockdowns))	• Unintended health, social and economic outcomes (Mental Health, violence, healthcare utilization)

(Ford et al., 2021)	21	Time Series, Case Control, Cross-sectional study, Synthetic control method, event study	African Region, Region of the Americas, South-East Asian Region, European Region, Western Pacific Region	community settings	General population	• Individual protection (Mask wearing)	• COVID-19 epidemiological outcomes (Risk and incidence, Mortality)
(Johanna, Citrawijaya, & Wangge, 2020)	18	Time Series, Cohort Study, Modeling Studies	Region of the Americas, South-East Asian Region, European Region, Western Pacific Region	Not specified	General population	• Active Surveillance (Mass testing RT-PCR testing)<br>• Multicomponent Interventions (Mass testing and lockdown)<br>• Lockdown	• COVID-19 epidemiological outcomes (Risk and incidence, Mortality, Transmission related outcomes)
(Neira et al., 2021)	7	Cross-sectional study	African Region, Region of the Americas, South-East Asian Region, European Region, Eastern Mediterranean Region, Western Pacific Region	Not specified	Adults	• Multicomponent Interventions (Response (quarantine) and social interactions)	• Unintended health, social and economic outcomes (Nutrition)
(Nussbaumer-Streit et al., 2020)	32	Modeling Studies	European Region, South-East Asian Region, Region of the Americas	Educational institutions, Healthcare facility, Occupational setting, household, point of entry, public institution	Individuals who had contact with confirmed or suspected cases of COVID-19, who travelled from countries with a declared outbreak, or who live in regions with high disease transmission.	• Response (Quarantine, Isolation)<br>• Multicomponent Interventions (Lockdown, Individual protection, Response, Services, Social interactions)	• COVID-19 epidemiological outcomes (Risk and incidence, Transmission-related outcomes, Mortality)
(Byambasuren, Beller, Clark, Collignon, & Glasziou, 2021)	5	Controlled before and after study, Case Control, Cohort Study	Region of the Americas, South-East Asian Region, European Region	Community setting, healthcare setting	General population	• Individual Protection (Any form of eye protection including face shields and variants, goggles, and glasses)	• COVID-19 epidemiological outcomes (Risk and incidence)
(Oliveira Carvalho, Hülsdünker, & Carson, 2021)	13	Cross-sectional study, Modeling Studies	European Region	Not specified	adolescent	• Lockdown	• Unintended health, social and economic outcomes (Mental Health)
(Galanis, Vraka, Fragkou, Bilali, & Kaitelidou, 2021)	14	Cross-sectional study	African Region, Region of the Americas, South-East Asian Region, European Region, Western Pacific Region	Healthcare facility	Adults	• Individual Protection (PPE usage)	• Unintended health, social and economic outcomes (Physiological outcomes)

(Panchal et al., 2021)	61	Longitudinal study, Cross-sectional study	Eastern Mediterranean Region (EMR), South-East Asian Region, Region of the Americas, Western Pacific Region, European Region	Not specified	Children/Adolescent	• Lockdown	• Unintended health, social and economic outcomes (Mental Health)
(Panda et al., 2020)	15	Cross-sectional study	Region of the Americas, South-East Asian Region, European Region, Western Pacific Region	Healthcare facility, educational institutions	Children/Adolescent	• Lockdown	• Unintended health, social and economic outcomes (Mental Health)
(Pizarro et al., 2022)	1	Randomized Controlled Trial	Region of the Americas, European Region	Occupational setting	Adults, Individuals who had contact with confirmed or suspected cases of COVID-19	• Active surveillance (Test-based attendance and standard 10-day self-isolation)	• COVID-19 epidemiological outcomes (Risk and incidence)<br>• Unintended health, social and economic outcomes (Absenteeism)
(Qathrin, Saryono, & Mekar Dwi, 2021)	7	Cross-sectional study	Eastern Mediterranean Region (EMR), South-East Asian Region, Western Pacific Region	Healthcare facility	Quarantined individuals	• Response (Quarantine)	• Unintended health, social and economic outcomes (Mental Health)
(Knight et al., 2021)	64	Longitudinal study, Cross-sectional study, Phenomenological study	African Region, Region of the Americas, South-East Asian Region, European Region, Western Pacific Region	Not specified	Adults	• Lockdown	• Unintended health, social and economic outcomes (Physical activity)
(Rajkumar et al., 2022)	74	Case Control, Longitudinal study, Cross-sectional study, Qualitative study mixed -method, quantitative study, descriptive study.	European Region, region of the Americas, East Asia regions, Eastern Mediterranean Region, Western Pacific Region, South-East Asian Region	Not specified	Quarantined individuals, Sport persons (athletes and chess players), adult, children and adolescent	• Response (Quarantine)	• Unintended health, social and economic outcomes (Mental Health)

(Rajmil et al., 2021)	22	Uncontrolled before and after study, Cohort Study, Longitudinal study, Cross-sectional study, Quantitative study	Eastern Mediterranean Region (EMR), Region of the Americas, Western Pacific Region, European Region, African Region	Not specified	Children/Adolescent	• Multicomponent Interventions (Services (School closures) and lockdown)<br>• Lockdown	• Unintended health, social and economic outcomes (Mental Health, violence, Nutrition, Physical activity, Healthcare Utilization)
(Regmi & Lwin, 2021)	33	Case Control, Cohort Study, Cross-sectional study, Qualitative study	Eastern Mediterranean Region (EMR), South-East Asian Region, Region of the Americas, Western Pacific Region, European Region, African Region	Community setting	General population	• Multicomponent Interventions (Response, Services, Social interactions, Movement, Individual protection)	• COVID-19 epidemiological outcomes (Transmission related outcomes)
(Rezwanul Hasan, Syed Afroz, Jeff, Jeff, & Jeff, 2021)	35	Quantitative study	Western Pacific Region	Not specified	Not specified	• Lockdown	• Unintended health, social and economic outcomes (Air quality)
(Rivera, Nys, & Fiestas, 2021)	7	Cohort Study, Cross-sectional study,	Region of the Americas, European Region, Western Pacific Region	Not specified	Children/Adolescent	• Lockdown	• Unintended health, social and economic outcomes (Physical activity)
(Rodriguez-Fernandez et al., 2021)	26	Longitudinal study, Cross-sectional study	European Region, region of the Americas, East Asia regions, Eastern Mediterranean Region, Western Pacific Region, South-East Asian Region,	Not specified	General population	• Social Interactions (Social Distancing)	• Unintended health, social and economic outcomes (Mental Health)
(Runacres et al., 2021)	40	Cross-sectional study, Observational study	Western Pacific Region, Region of the Americas, European Region, Eastern Mediterranean Region	Educational institutions, occupational settings, community setting	General population	• Multicomponent Interventions (Social distancing, quarantine, and lockdown/homestay requirements)	• Unintended health, social and economic outcomes (Physical activity)

(Hawco et al., 2022)	38	Time Series, Cohort Study	Region of the Americas, European Region, Western Pacific Region, South-East Asian Region	Healthcare facility	Perinatal women	• Multicomponent Interventions (Individual protection, social interactions, Services, Movement)	• Unintended health, social and economic outcomes (Healthcare Utilization; Change in incidence and mortality of diseases other than COVID-19)
(Krishnaratne et al., 2022)	38	Quasi-randomized Controlled Trial, Uncontrolled before and after study, Longitudinal study, Modeling Studies	Region of the Americas, South-East Asian Region, European Region, Western Pacific Region	Educational institutions	General population	• Services (Measures reducing the opportunity for contacts (i.e., alternating attendance, reduced class size))<br>• Social Interactions (General physical distancing policies)<br>• Individual Protection (Mask wearing, Handwashing)<br>• Multicomponent interventions	• COVID-19 epidemiological outcomes (Risk and incidence, Transmission-related outcomes, Mortality, Number of days spent in school, hospitalization)
(Samji et al., 2021)	116	Case Control, Cohort Study, Cross-sectional study, Qualitative study	African Region, Region of the Americas, South-East Asian Region, European Region, Eastern Mediterranean Region, Western Pacific Region	Healthcare facility, educational institutions, household setting	Children/Adolescent, Adults	• Services (School closures)<br>• Social Interactions (Physical Distancing)<br>• Multicomponent Interventions (Response, social interactions)<br>• Lockdown	• Unintended health, social and economic outcomes (Mental Health)
(Saulle, Minozzi, Amato, & Davoli, 2021)	42	Uncontrolled before and after study, Cohort Study, Cross-sectional study, Modeling Studies	Region of the Americas, European Region, Eastern Mediterranean Region, Western Pacific Region	Not specified	Children/Adolescent	• Multicomponent interventions (Social distancing measures)	• Unintended health, social and economic outcomes (Nutrition, Physical activity, Sleep, violence, Healthcare Utilization)
(Schmidt et al., 2021)	53	Cross-sectional study	Western Pacific Region, Region of the Americas, European Region	Educational institutions, occupational settings, community setting, entertainment facilities	General population	• Response (Quarantine)<br>• Multicomponent Interventions (Movement, services; social interactions)	• Unintended health, social and economic outcomes (Nutrition, Substance use)
(Shekaraiah & Suresh, 2021)	10	Cross-sectional study, Prospective exploratory experimental	Region of the Americas, European Region, Western Pacific Region	Not specified	General population	• Individual Protection (Masks (surgical, N95/KN95, and cloth masks))	• Unintended health, social and economic outcomes (Voice production)

(Sideli et al., 2021)	26	Case Control, Longitudinal study, Cross-sectional study	Region of the Americas, European Region; Western Pacific Region,	Not specified	People with pre-existing conditions	• Lockdown	• Unintended health, social and economic outcomes (Nutrition)
(Sohi et al., 2022)	27	Cohort Study, Cross-sectional study	Region of the Americas, European Region, South-East Asian Region, Western Pacific Region	Not specified	General population	• Multicomponent Interventions (Social interactions and services)<br>• Lockdown	• Unintended health, social and economic outcomes (Nutrition, substance use)
(Talic et al., 2021)	72	Randomized Controlled Trial, Quasi-randomized Controlled Trial, Time Series, Case Control, Cohort Study, Longitudinal study, Cross-sectional study, Natural experiment	Eastern Mediterranean Region (EMR), South-East Asian Region, Region of the Americas, Western Pacific Region, European Region, African Region	Not specified	General population	• Active Surveillance (Screening for fever)<br>• Response (Quarantine or isolation)<br>• Services (School closures; Business closures)<br>• Social Interactions (Contact-restriction procedures such as physical distancing)<br>• Movement (Restricted travel and border closures)<br>• Physical environment (Disinfection in household)<br>• Individual Protections (Mask wearing; Handwashing)<br>• Multicomponent Interventions (Lockdown and social interactions; services, response and social interactions; response, social interactions and lockdown; social interactions and individual protection; services and social interactions; surveillance, movement and services)<br>• Lockdown	• COVID-19 epidemiological outcomes (Risk and incidence, Transmission-related outcomes, Mortality)

(Stephanie et al., 2021)	66	Cohort Study, Longitudinal study, Cross-sectional study, Observational study	African Region, Region of the Americas, South-East Asian Region, European Region, Eastern Mediterranean Region, Western Pacific Region	Not specified	General population	• Lockdown	• Unintended health, social and economic outcomes (Physical Activity)
(Suárez-González, Rajagopalan, Livingston, & Alladi, 2021)	15	Uncontrolled before and after study, Cross-sectional study	Region of the Americas, European Region	Not specified	People with pre-existing conditions	• Lockdown	• Unintended health, social and economic outcomes (Mental Health)
(Girum et al., 2021)	25	Case Control, Cohort Study, Cross-sectional study, Modeling Studies	African Region, Region of the Americas, South-East Asian Region, European Region, Western Pacific Region	Community setting	Individuals who had contact with confirmed or suspected cases of COVID-19, who travelled from countries with a declared outbreak, or who live in regions with high disease transmission.	• Social interactions (Social Distancing)<br>• Movement (Travel ban)<br>• Multicomponent Interventions (Movement and lockdown)<br>• Lockdown	• COVID-19 epidemiological outcomes (Transmission-related outcomes, Risk and incidence, Mortality)
(Tabatabaeizadeh, 2021)	4	Case Control, descriptive study	Region of the Americas, Western Pacific Region, South-East Asian Region	Not specified	General population	• Face Mask	• COVID-19 epidemiological outcomes (Risk and incidence)
(Chang et al., 2021)	12	Cohort Study, Cross-sectional study	Eastern Mediterranean Region (EMR), Region of the Americas, Western Pacific Region	Not specified	Children/Adolescent	• Lockdown	• Unintended health, social and economic outcomes (Nutrition)
(Tully et al., 2021)	14	Not reported	African Region, Region of the Americas, South-East Asian Region, European Region, Western Pacific Region	Not specified	General population	• Social interactions (stay-at-home orders)<br>• Individual protection (Mask)<br>• Multicomponent Interventions (Public health restrictions)	• Unintended health, social and economic outcomes (Mobility)
(Viner et al., 2022)	36	Uncontrolled before and after study, Time Series, Cohort Study, Cross-sectional study, Modeling Studies, Parallel comparative study	Region of the Americas, South-East Asian Region, European Region, Western Pacific Region	Educational institutions	Children/Adolescent	• Services	• Unintended health, social and economic outcomes (Sleep, Violence, Mental Health, Nutrition, Physical Activity)

(Viswanathan et al., 2020)	2	Modeling Studies	Region of the Americas	Point of entry (airport) and health facilities	General population	• Active surveillance (Symptom screening and<br>• Laboratory test screening)	• COVID-19 epidemiological outcomes (Risk and incidence)
(Wall & Dempsey, 2022)	16	Cohort Study, Cross-sectional study, Modeling Studies	European region, Region of the Americas, Eastern Mediterranean Region, African region	Not specified	Perinatal women	• Lockdown	• Unintended health, social and economic outcomes (Mental Health)
(K. A. Walsh et al., 2021)	11	Randomized Controlled Trial, Uncontrolled before and after study, Mechanistic study	Eastern Mediterranean Region (EMR), South-East Asian Region, Western Pacific Region, European Region	Community Settings, Occupational setting, entertainment facility	General population	• Multicomponent interventions (Surveillance, individual protection, and physical environment; Surveillance, contact tracing, Response)	• COVID-19 epidemiological outcomes (Transmission related outcomes)
(Walsh et al., 2022)	16	Randomized Controlled Trial, Controlled before and after study, Cross-sectional study, Case series	Region of the Americas, European Region	Community setting Healthcare setting, Entertainment facility; Educational institutions	General population	• Active Surveillance (Rapid antigen testing for the screening)	• COVID-19 epidemiological outcomes (Transmission related outcomes)
(S. Walsh et al., 2021)	40	Uncontrolled before and after study, Cohort Study	Region of the Americas, European Region, Western Pacific Region	Educational institutions	Children/Adolescent	• Services (School closures and reopening)	• COVID-19 epidemiological outcomes (Transmission related outcomes)
(Jin, Sun, Zheng, & An, 2021)	28	Not reported	Region of the Americas, South-East Asian Region, European Region	Healthcare facility, educational institutions	children, adults	• Response (Quarantine)	• Unintended health, social and economic outcomes (Mental Health)
(Yaacoub et al., 2021)	3	Case Control, Modeling Studies	Region of the Americas, South-East Asian Region, European Region, Western Pacific Region	Entertainment facilities (e.g., malls, stadium)	General population	• Multicomponent Interventions (Social interactions, individual protection, physical environment)	• COVID-19 epidemiological outcomes (Transmission related outcomes)

(Yaghoubi, Salimi, & Meskarpour-Amiri, 2021)	82	Case Control, Cohort Study, Cross-sectional study,<br>Case series	European Region, region of the Americas, East Asia regions, Eastern Mediterranean Region, Western Pacific Region, South-East Asian Region	Healthcare facility	healthcare workers	• Response (Quarantine)	• Unintended health, social and economic outcomes (Mental health, Absenteeism)
(Zaccagni, Toselli, & Barbieri, 2021)	23	Cross-sectional study	European Region	Not specified	General population	• Lockdown	• Unintended health, social and economic outcomes (Physical Activity)

Alex, R. P., Alex, R. P., Wesley, G. J., Erin, J., Catherine, K., & Felicia Maria, K. (2021). Evidence from a systematic review and meta-analysis: Domestic Violence during the COVID-19 Pandemic. *Journal of Criminal Justice*. doi:10.1016/J.JCRIMJUS.2021.101806

Alkatout, I., Biebl, M., Momenimovahed, Z., Giovannucci, E., Hadavandsiri, F., Salehiniya, H., & Allahqoli, L. (2021). Has COVID-19 Affected Cancer Screening Programs? A Systematic Review. *Frontiers in oncology, 11*, 675038. doi:10.3389/fonc.2021.675038

Antonio, S. G., Chiara, D. P., Ilaria, D., Matteo, M., & Edoardo, M. (2021). Glucose control in diabetes during home confinement for the first pandemic wave of COVID-19: a meta-analysis of observational studies. *Acta diabetologica*. doi:10.1007/s00592-021-01754-2

Asín-Izquierdo, I., Ruiz-Ranz, E., & Arévalo-Baeza, M. (2022). The Physiological Effects of Face Masks During Exercise Worn Due to COVID-19: A Systematic Review. *Sports health*, 19417381221084661. doi:10.1177/19417381221084661

Ayouni, I., Maatoug, J., Dhouib, W., Zammit, N., Fredj, S. B., Ghammam, R., & Ghannem, H. (2021). Effective public health measures to mitigate the spread of COVID-19: a systematic review. *BMC public health, 21*(1), 1015. doi:10.1186/s12889-021-11111-1

Bakaloudi, D. R., Barazzoni, R., Bischoff, S. C., Breda, J., Wickramasinghe, K., & Chourdakis, M. (2021). Impact of the first COVID-19 lockdown on body weight: A combined systematic review and a meta-analysis. *Clinical nutrition (Edinburgh, Scotland)*. doi:10.1016/j.clnu.2021.04.015

Bakaloudi, D. R., Jeyakumar, D. T., Jayawardena, R., & Chourdakis, M. (2021). The impact of COVID-19 lockdown on snacking habits, fast-food and alcohol consumption: A systematic review of the evidence. *Clinical nutrition (Edinburgh, Scotland)*. doi:10.1016/j.clnu.2021.04.020

Baumhardt, M., Dreyhaupt, J., Winsauer, C., Stuhler, L., Thiessen, K., Stephan, T., . . . Rattka, M. (2021). The Effect of the Lockdown on Patients With Myocardial Infarction During the COVID- 19 Pandemic-a Systematic Review and Meta-Analysis. *Deutsches Arzteblatt international, 118*(Forthcoming), 253. doi:10.3238/arztebl.m2021.0253

Bonati, M., Campi, R., & Segre, G. (2022). Psychological impact of the quarantine during the COVID-19 pandemic on the general European adult population: a systematic review of the evidence. *Epidemiology and psychiatric sciences, 31*, e27. doi:10.1017/S2045796022000051

Bou-Karroum, L., Khabsa, J., Jabbour, M., Hilal, N., Haidar, Z., Abi Khalil, P., . . . Bcheraoui, C. E. (2021). Public Health Effects of Travel-Related Policies on the COVID-19 Pandemic: A Mixed-Methods Systematic Review. *The Journal of infection*. doi:10.1016/j.jinf.2021.07.017

Brakspear, L., Boules, D., Nicholls, D., & Burmester, V. (2022). The Impact of COVID-19-Related Living Restrictions on Eating Behaviours in Children and Adolescents: A Systematic Review. *Nutrients, 14*(17). doi:10.3390/nu14173657

Burns, J., Movsisyan, A., Stratil, J. M., Biallas, R. L., Coenen, M., Emmert-Fees, K. M., . . . Rehfuess, E. (2021). International travel-related control measures to contain the COVID-19 pandemic: a rapid review. *The Cochrane database of systematic reviews, 3*(3), CD013717. doi:10.1002/14651858.CD013717.pub2

Byambasuren, O., Beller, E., Clark, J., Collignon, P., & Glasziou, P. (2021). The effect of eye protection on SARS-CoV-2 transmission: a systematic review. *Antimicrobial resistance and infection control, 10*(1), 156. doi:10.1186/s13756-021-01025-3

Camacho-Montaño, L. R., Iranzo, A., Martínez-Piédrola, R. M., Camacho-Montaño, L. M., Huertas-Hoyas, E., Serrada-Tejeda, S., . . . de Heredia-Torres, M. P. (2022). Effects of COVID-19 home confinement on sleep in children: A systematic review. *Sleep medicine reviews, 62*, 101596. doi:10.1016/j.smrv.2022.101596

Cardwell, K., Jordan, K., Byrne, P., Harrington, P., Ryan, M., O'Neill, M., . . . Ryan, M. (2021). The effectiveness of non-contact thermal screening as a means of identifying cases of Covid-19: a rapid review of the evidence. *Rev. Med. Virol., 31*(4), e2192. doi:10.1002/rmv.2192

Caristia, S., Ferranti, M., Skrami, E., Raffetti, E., Pierannunzio, D., Palladino, R., . . . lockdowns, A. I. E. w. g. o. t. e. o. t. e. o. (2020). Effect of national and local lockdowns on the control of COVID-19 pandemic: a rapid review. *Epidemiologia e prevenzione, 44*(5-6 Suppl 2), 60-68. doi:10.19191/EP20.5-6.S2.104

Castaldelli-Maia, J. M., Marziali, M. E., Lu, Z., & Martins, S. S. (2021). Investigating the effect of national government physical distancing measures on depression and anxiety during the COVID-19 pandemic through meta-analysis and meta-regression. *Psychological medicine, 51*(6), 1-46. doi:10.1017/S0033291721000933

Cavicchioli, M., Ferrucci, R., Guidetti, M., Canevini, M. P., Pravettoni, G., & Galli, F. (2021). What Will Be the Impact of the Covid-19 Quarantine on Psychological Distress? Considerations Based on a Systematic Review of Pandemic Outbreaks. *Healthcare (Basel, Switzerland), 9*(1). doi:10.3390/healthcare9010101

Chaabane, S., Doraiswamy, S., Chaabna, K., Mamtani, R., & Cheema, S. (2021). The Impact of COVID-19 School Closure on Child and Adolescent Health: A Rapid Systematic Review. *Children (Basel, Switzerland), 8*(5). doi:10.3390/children8050415

Chai, J., Xu, H., An, N., Zhang, P., Liu, F., He, S., . . . Li, Y. (2021). The Prevalence of Mental Problems for Chinese Children and Adolescents During COVID-19 in China: A Systematic Review and Meta-Analysis. *Frontiers in pediatrics, 9*, 661796. doi:10.3389/fped.2021.661796

Chang, T. H., Chen, Y. C., Chen, W. Y., Chen, C. Y., Hsu, W. Y., Chou, Y., & Chang, Y. H. (2021). Weight Gain Associated with COVID-19 Lockdown in Children and Adolescents: A Systematic Review and Meta-Analysis. *Nutrients, 13*(10). doi:10.3390/nu13103668

Daniela, R., Susan, A., Barbara, C., Laura, C., Susan, S., Kieran, A. W., . . . Michelle, O. N. (2020). Effectiveness of face masks worn in community settings at reducing the transmission of SARS-CoV-2: A rapid review. *3*. doi:10.12688/HRBOPENRES.13161.1

Della Valle, P. G., Mosconi, G., Nucci, D., Vigezzi, G. P., Gentile, L., Gianfredi, V., . . . Odone, A. (2021). Adherence to the Mediterranean Diet during the COVID-19 national lockdowns: a systematic review of observational studies. *Acta bio-medica : Atenei Parmensis, 92*(S6), e2021440. doi:10.23750/abm.v92iS6.12233

Desye, B. (2021). COVID-19 Pandemic and Water, Sanitation, and Hygiene: Impacts, Challenges, and Mitigation Strategies. *Environmental health insights, 15*, 11786302211029447. doi:10.1177/11786302211029447

Elisabeth, A. L., Karlen, S. B., & Magkos, F. (2021). The Effect of COVID-19-related Lockdowns on Diet and Physical Activity in Older Adults: A Systematic Review. *Aging and disease, 12*(8), 1935-1947. doi:10.14336/AD.2021.0606

Farooq, S., Tunmore, J., Ali, W., & Ayub, M. (2021). Suicide, self-harm and suicidal ideation during COVID-19: A systematic review. *Psychiatry research, 306*, 114228. doi:10.1016/j.psychres.2021.114228

Ford, N., Holmer, H. K., Chou, R., Villeneuve, P. J., Baller, A., Van Kerkhove, M., & Allegranzi, B. (2021). Mask use in community settings in the context of COVID-19: A systematic review of ecological data. *EClinicalMedicine, 38*, 101024. doi:10.1016/j.eclinm.2021.101024

Freiberg, A., Schubert, M., Romero Starke, K., Hegewald, J., & Seidler, A. (2021). A Rapid Review on the Influence of COVID-19 Lockdown and Quarantine Measures on Modifiable Cardiovascular Risk Factors in the General Population. *International journal of environmental research and public health, 18*(16). doi:10.3390/ijerph18168567

Fricke, L. M., Glöckner, S., Dreier, M., & Lange, B. (2020). Impact of non-pharmaceutical interventions targeted at COVID-19 pandemic on influenza burden - a systematic review. *The Journal of infection, 82*(1), 1-35. doi:10.1016/j.jinf.2020.11.039

Galanis, P., Vraka, I., Fragkou, D., Bilali, A., & Kaitelidou, D. (2021). Impact of personal protective equipment use on health care workers' physical health during the COVID-19 pandemic: a systematic review and meta-analysis. *American journal of infection control*. doi:10.1016/j.ajic.2021.04.084

Garofolo, M., Aragona, M., Rodia, C., Falcetta, P., Bertolotto, A., Campi, F., . . . Penno, G. (2021). Glycaemic control during the lockdown for COVID-19 in adults with type 1 diabetes: A meta-analysis of observational studies. *Diabetes research and clinical practice*, 109066. doi:10.1016/j.diabres.2021.109066

Girum, T., Lentiro, K., Geremew, M., Migora, B., & Shewamare, S. (2020). Global strategies and effectiveness for COVID-19 prevention through contact tracing, screening, quarantine, and isolation: a systematic review. *Trop. Med. Health, 48*(1), 91. doi:10.1186/S41182-020-00285-W

Girum, T., Lentiro, K., Geremew, M., Migora, B., Shewamare, S., & Shimbre, M. S. (2021). Optimal strategies for COVID-19 prevention from global evidence achieved through social distancing, stay at home, travel restriction and lockdown: a systematic review. *Archives of public health = Archives belges de sante publique, 79*(1), 150. doi:10.1186/s13690-021-00663-8

Grekousis, G., & Liu, Y. (2021). Digital contact tracing, community uptake, and proximity awareness technology to fight COVID-19: a systematic review. *Sustainable cities and society, 71*, 102995. doi:10.1016/j.scs.2021.102995

Grépin, K. A., Ho, T. L., Liu, Z., Marion, S., Piper, J., Worsnop, C. Z., & Lee, K. (2021). Evidence of the effectiveness of travel-related measures during the early phase of the COVID-19 pandemic: a rapid systematic review. *BMJ global health, 6*(3). doi:10.1136/bmjgh-2020-004537

Hammerstein, S., König, C., Dreisörner, T., & Frey, A. (2021). Effects of COVID-19-Related School Closures on Student Achievement-A Systematic Review. *Frontiers in psychology, 12*, 746289. doi:10.3389/fpsyg.2021.746289

Hatami, H., Qaderi, S., Shah, J., Rezaeian, A. R., Farsi, Y., Alinasab, F., . . . Shah, A. (2022). COVID-19: National Pandemic Management Strategies and their Efficacies and Impacts on the Number of Secondary Cases and Prognosis: A Systematic Review. *International journal of preventive medicine, 13*, 100. doi:10.4103/ijpvm.IJPVM_464_20

Hawco, S., Rolnik, D. L., Woolner, A., Cameron, N. J., Wyness, V., Mol, B. W., & Black, M. (2022). The impact of mitigation measures on perinatal outcomes during the first nine months of the COVID-19 pandemic: A systematic review with meta-analysis. *European journal of obstetrics, gynecology, and reproductive biology, 274*, 117-127. doi:10.1016/j.ejogrb.2022.05.007

Hossain, A. D., Jarolimova, J., Elnaiem, A., Huang, C. X., Richterman, A., & Ivers, L. C. (2022). Effectiveness of contact tracing in the control of infectious diseases: a systematic review. *The Lancet. Public health*. doi:10.1016/S2468-2667(22)00001-9

Hugelius, K., Harada, N., & Marutani, M. (2021). Consequences of visiting restrictions during the COVID-19 pandemic: An integrative review. *International journal of nursing studies, 121*, 104000. doi:10.1016/j.ijnurstu.2021.104000

Iezadi, S., Gholipour, K., Azami-Aghdash, S., Ghiasi, A., Rezapour, A., Pourasghari, H., & Pashazadeh, F. (2021). Effectiveness of non-pharmaceutical public health interventions against COVID-19: A systematic review and meta-analysis. *PloS one, 16*(11), e0260371. doi:10.1371/journal.pone.0260371

Ingram, C., Downey, V., Roe, M., Chen, Y., Archibald, M., Kallas, K. A., . . . Perrotta, C. (2021). COVID-19 Prevention and Control Measures in Workplace Settings: A Rapid Review and Meta-Analysis. *International journal of environmental research and public health, 18*(15). doi:10.3390/ijerph18157847

Jabs, J. M., Schwabe, A., Wollkopf, A. D., Gebel, B., Stadelmaier, J., Erdmann, S., . . . Mutters, N. T. (2022). The role of routine SARS-CoV-2 screening of healthcare-workers in acute care hospitals in 2020: a systematic review and meta-analysis. *BMC infectious diseases, 22*(1), 587. doi:10.1186/s12879-022-07554-5

Jenniskens, K., Bootsma, M. C. J., Damen, J., Oerbekke, M. S., Vernooij, R. W. M., Spijker, R., . . . Hooft, L. (2021). Effectiveness of contact tracing apps for SARS-CoV-2: a rapid systematic review. *BMJ open, 11*(7), e050519. doi:10.1136/bmjopen-2021-050519

Jin, Y., Sun, T., Zheng, P., & An, J. (2021). Mass quarantine and mental health during COVID-19: A meta-analysis. *Journal of affective disorders, 295*, 1335-1346. doi:10.1016/j.jad.2021.08.067

Johanna, N., Citrawijaya, H., & Wangge, G. (2020). Mass screening vs lockdown vs combination of both to control COVID-19: A systematic review. *Journal of public health research, 9*(4), 2011. doi:10.4081/jphr.2020.2011

Kharel, M., Sakamoto, J. L., Carandang, R. R., Ulambayar, S., Shibanuma, A., Yarotskaya, E., . . . Jimba, M. (2022). Impact of COVID-19 pandemic lockdown on movement behaviours of children and adolescents: a systematic review. *BMJ global health, 7*(1). doi:10.1136/bmjgh-2021-007190

Khatib, M. N., Sinha, A., Mishra, G., Quazi, S. Z., Gaidhane, S., Saxena, D., . . . Zahiruddin, Q. S. (2022). WASH to control COVID-19: A rapid review. *Frontiers in public health, 10*, 976423. doi:10.3389/fpubh.2022.976423

Khosravizadeh, O., Ahadinezhad, B., Maleki, A., Najafpour, Z., & Golmohammadi, R. (2021). Social distance capacity to control the COVID-19 pandemic: A systematic review on time series analysis. *The International journal of risk & safety in medicine*. doi:10.3233/JRS-210037

Knight, R. L., McNarry, M. A., Sheeran, L., Runacres, A. W., Thatcher, R., Shelley, J., & Mackintosh, K. A. (2021). Moving Forward: Understanding Correlates of Physical Activity and Sedentary Behaviour during COVID-19-An Integrative Review and Socioecological Approach. *International journal of environmental research and public health, 18*(20). doi:10.3390/ijerph182010910

Kourti, A., Stavridou, A., Panagouli, E., Psaltopoulou, T., Spiliopoulou, C., Tsolia, M., . . . Tsitsika, A. (2021). Domestic Violence During the COVID-19 Pandemic: A Systematic Review. *Trauma, violence & abuse*, 15248380211038690. doi:10.1177/15248380211038690

Kourti, A., Stavridou, A., Panagouli, E., Psaltopoulou, T., Tsolia, M., Sergentanis, T. N., & Tsitsika, A. (2021). Play Behaviors in Children during the COVID-19 Pandemic: A Review of the Literature. *Children (Basel, Switzerland), 8*(8). doi:10.3390/children8080706

Krishnaratne, S., Littlecott, H., Sell, K., Burns, J., Rabe, J. E., Stratil, J. M., . . . Pfadenhauer, L. M. (2022). Measures implemented in the school setting to contain the COVID-19 pandemic. *Cochrane Database Syst Rev, 1*(1), Cd015029. doi:10.1002/14651858.Cd015029

Kunstler, B., Newton, S., Hill, H., Ferguson, J., Hore, P., Mitchell, B. G., . . . Turner, T. (2022). P2/N95 respirators & surgical masks to prevent SARS-CoV-2 infection: Effectiveness & adverse effects. *Infection, disease & health*. doi:10.1016/j.idh.2022.01.001

Lausi, G., Pizzo, A., Cricenti, C., Baldi, M., Desiderio, R., Giannini, A. M., & Mari, E. (2021). Intimate Partner Violence during the COVID-19 Pandemic: A Review of the Phenomenon from Victims' and Help Professionals' Perspectives. *International journal of environmental research and public health, 18*(12). doi:10.3390/ijerph18126204

Mbwogge, M. (2021). Mass Testing With Contact Tracing Compared to Test and Trace for the Effective Suppression of COVID-19 in the United Kingdom: Systematic Review. *JMIRx med, 2*(2), e27254. doi:10.2196/27254

Mendez-Brito, A., Bcheraoui, C. E., & Pozo-Martin, F. (2021). Systematic review of empirical studies comparing the effectiveness of non-pharmaceutical interventions against COVID-19. *The Journal of infection*. doi:10.1016/j.jinf.2021.06.018

Mignogna, C., Costanzo, S., Ghulam, A., Cerletti, C., Donati, M. B., de Gaetano, G., . . . Bonaccio, M. (2021). Impact of Nationwide Lockdowns Resulting from The First Wave of the COVID-19 Pandemic on Food Intake, Eating Behaviours and Diet Quality: A Systematic Review. *Advances in nutrition (Bethesda, Md.)*. doi:10.1093/advances/nmab130

Minozzi, S., Saulle, R., Amato, L., & Davoli, M. (2021). [Impact of social distancing for covid-19 on the psychological well-being of youths: a systematic review of the literature.]. *Recenti progressi in medicina, 112*(5), 360-370. doi:10.1701/3608.35873

Neira, C., Mardones, R., Neira, C., Godinho, R., Rincon, F., Pedroso, J., & Pedroso, J. (2021). Consequences of the covid-19 syndemic for nutritional health: A systematic review. *Nutrients, 13*(4). doi:10.3390/nu13041168

Nussbaumer-Streit, B., Mayr, V., Dobrescu, A. I., Chapman, A., Persad, E., Klerings, I., . . . Gartlehner, G. (2020). Quarantine alone or in combination with other public health measures to control COVID-19: a rapid review. *Cochrane Database of Systematic Reviews, 2020*(9), CD013574. doi:10.1002/14651858.CD013574.pub2

Oliveira Carvalho, P., Hülsdünker, T., & Carson, F. (2021). The Impact of the COVID-19 Lockdown on European Students' Negative Emotional Symptoms: A Systematic Review and Meta-Analysis. *Behavioral sciences (Basel, Switzerland), 12*(1). doi:10.3390/bs12010003

Panchal, U., Salazar de Pablo, G., Franco, M., Moreno, C., Parellada, M., Arango, C., & Fusar-Poli, P. (2021). The impact of COVID-19 lockdown on child and adolescent mental health: systematic review. *European child & adolescent psychiatry*. doi:10.1007/s00787-021-01856-w

Panda, P. K., Gupta, J., Chowdhury, S. R., Kumar, R., Meena, A. K., Madaan, P., . . . Gulati, S. (2020). Psychological and Behavioral Impact of Lockdown and Quarantine Measures for COVID-19 Pandemic on Children, Adolescents and Caregivers: A Systematic Review and Meta-Analysis. *Journal of tropical pediatrics, 67*(1). doi:10.1093/tropej/fmaa122

Pizarro, A. B., Persad, E., Durao, S., Nussbaumer-Streit, B., Engela-Volker, J. S., McElvenny, D., . . . Bruschettini, M. (2022). Workplace interventions to reduce the risk of SARS-CoV-2 infection outside of healthcare settings. *The Cochrane database of systematic reviews, 5*, CD015112. doi:10.1002/14651858.CD015112.pub2

Qathrin, N., Saryono, S., & Mekar Dwi, A. (2021). The Impact of Centralized Quarantine on Mental Health of People Affected By Covid-19: A Systematic Review. *6*(1). doi:10.30604/JIKA.V6I1.477

Rajkumar, E., Rajan, A. M., Daniel, M., Lakshmi, R., John, R., George, A. J., . . . Varghese, J. (2022). The psychological impact of quarantine due to COVID-19: A systematic review of risk, protective factors and interventions using socio-ecological model framework. *Heliyon, 8*(6), e09765. doi:10.1016/j.heliyon.2022.e09765

Rajmil, L., Hjern, A., Boran, P., Gunnlaugsson, G., Kraus De Camargo, O., Raman, S., & Raman, S. (2021). Impact of lockdown and school closure on children's health and well-being during the first wave of COVID-19: A narrative review. *BMJ Paediatr. Open, 5*(1), e001043. doi:10.1136/bmjpo-2021-001043

Regmi, K., & Lwin, C. M. (2021). Factors associated with the implementation of non-pharmaceutical interventions for reducing coronavirus disease 2019 (COVID-19): A systematic review. *International journal of environmental research and public health, 18*(8), 4274.

Rezwanul Hasan, R., Syed Afroz, K., Jeff, G., Jeff, G., & Jeff, G. (2021). A Systematic Literature Review of the Impact of COVID-19 Lockdowns on Air Quality in China. *Aerosol and Air Quality Research, 21*. doi:10.4209/AAQR.200614

Rivera, P. A., Nys, B. L., & Fiestas, F. (2021). Impact of COVID-19 induced lockdown on physical activity and sedentary behavior among university students: A systematic review. *Medwave, 21*(8), e8456. doi:10.5867/medwave.2021.08.8456

Rodriguez-Fernandez, P., Gonzalez-Santos, J., Santamaria-Pelaez, M., Soto-Camara, R., Gonzalez-Bernal, J. J., & Sanchez-Gonzalez, E. (2021). Psychological effects of home confinement and social distancing derived from covid-19 in the general population—a systematic review. *Int. J. Environ. Res. Public Health, 18*(12). doi:10.3390/ijerph18126528

Runacres, A., Mackintosh, K. A., Knight, R. L., Sheeran, L., Thatcher, R., Shelley, J., & McNarry, M. A. (2021). Impact of the COVID-19 Pandemic on Sedentary Time and Behaviour in Children and Adults: A Systematic Review and Meta-Analysis. *International journal of environmental research and public health, 18*(21). doi:10.3390/ijerph182111286

Samji, H., Wu, J., Ladak, A., Vossen, C., Stewart, E., Dove, N., . . . Snell, G. (2021). Review: Mental health impacts of the COVID-19 pandemic on children and youth - a systematic review. *Child and adolescent mental health*. doi:10.1111/camh.12501

Saulle, R., Minozzi, S., Amato, L., & Davoli, M. (2021). [Impact of social distancing for covid-19 on youths' physical health: a systematic review of the literature.]. *Recenti progressi in medicina, 112*(5), 347-359. doi:10.1701/3608.35872

Schmidt, R. A., Genois, R., Jin, J., Vigo, D., Rehm, J., & Rush, B. (2021). The early impact of COVID-19 on the incidence, prevalence, and severity of alcohol use and other drugs: A systematic review. *Drug and alcohol dependence, 228*, 109065. doi:10.1016/j.drugalcdep.2021.109065

Shekaraiah, S., & Suresh, K. (2021). Effect of Face Mask on Voice Production During COVID-19 Pandemic: A Systematic Review. *Journal of voice : official journal of the Voice Foundation*. doi:10.1016/j.jvoice.2021.09.027

Sideli, L., Lo Coco, G., Bonfanti, R. C., Borsarini, B., Fortunato, L., Sechi, C., & Micali, N. (2021). Effects of COVID-19 lockdown on eating disorders and obesity: A systematic review and meta-analysis. *European eating disorders review : the journal of the Eating Disorders Association*. doi:10.1002/erv.2861

Sohi, I., Chrystoja, B. R., Rehm, J., Wells, S., Monteiro, M., Ali, S., & Shield, K. D. (2022). Changes in alcohol use during the COVID-19 pandemic and previous pandemics: A systematic review. *Alcoholism, clinical and experimental research, 46*(4), 498-513. doi:10.1111/acer.14792

Stephanie, S., Mike, T., Mark, T., Jae, S., Yvonne, B., Laurie, B., . . . Lee, S. (2021). Changes in physical activity and sedentary behaviours from before to during the COVID-19 pandemic lockdown: a systematic review. *BMJ open sport and exercise medicine, 7*(1), e000960. doi:10.1136/bmjsem-2020-000960

Stratil, J. M., Biallas, R. L., Burns, J., Arnold, L., Geffert, K., Kunzler, A. M., . . . Movsisyan, A. (2021). Non-pharmacological measures implemented in the setting of long-term care facilities to prevent SARS-CoV-2 infections and their consequences: a rapid review. *The Cochrane database of systematic reviews, 9*, CD015085. doi:10.1002/14651858.CD015085.pub2

Suárez-González, A., Rajagopalan, J., Livingston, G., & Alladi, S. (2021). The effect of COVID-19 isolation measures on the cognition and mental health of people living with dementia: A rapid systematic review of one year of quantitative evidence. *EClinicalMedicine, 39*, 101047. doi:10.1016/j.eclinm.2021.101047

Tabatabaeizadeh, S. A. (2021). Airborne transmission of COVID-19 and the role of face mask to prevent it: a systematic review and meta-analysis. *European journal of medical research, 26*(1), 1. doi:10.1186/s40001-020-00475-6

Talic, S., Shah, S., Wild, H., Gasevic, D., Maharaj, A., Ademi, Z., . . . Ilic, D. (2021). Effectiveness of public health measures in reducing the incidence of covid-19, SARS-CoV-2 transmission, and covid-19 mortality: systematic review and meta-analysis. *BMJ (Clinical research ed.), 375*, e068302. doi:10.1136/bmj-2021-068302

Tully, M. A., McMaw, L., Adlakha, D., Blair, N., McAneney, J., McAneney, H., . . . Smith, L. (2021). The effect of different COVID-19 public health restrictions on mobility: A systematic review. *PloS one, 16*(12), e0260919. doi:10.1371/journal.pone.0260919

Viner, R., Russell, S., Saulle, R., Croker, H., Stansfield, C., Packer, J., . . . Minozzi, S. (2022). School Closures During Social Lockdown and Mental Health, Health Behaviors, and Well-being Among Children and Adolescents During the First COVID-19 Wave: A Systematic Review. *JAMA pediatrics*. doi:10.1001/jamapediatrics.2021.5840

Viswanathan, M., Kahwati, L., Hill, C., Jahn, B., Giger, K., Dobrescu, A. I., . . . Gartlehner, G. (2020). Universal screening for SARS-CoV-2 infection: a rapid review. *Cochrane Database of Systematic Reviews, 2020*(9), CD013718. doi:10.1002/14651858.CD013718

Wall, S., & Dempsey, M. (2022). The effect of COVID-19 lockdowns on women's perinatal mental health: a systematic review. *Women and birth : journal of the Australian College of Midwives*. doi:10.1016/j.wombi.2022.06.005

Walsh, K. A., Broderick, N., Ahern, S., Fawsitt, C. G., O'Brien, K. M., Carrigan, M., . . . Ryan, M. (2022). Effectiveness of rapid antigen testing for screening of asymptomatic individuals to limit the transmission of SARS-CoV-2: A rapid review. *Reviews in medical virology*, e2350. doi:10.1002/rmv.2350

Walsh, K. A., Tyner, B., Broderick, N., Harrington, P., O'Neill, M., Fawsitt, C. G., . . . Ryan, M. (2021). Effectiveness of public health measures to prevent the transmission of SARS-CoV-2 at mass gatherings: A rapid review. *Reviews in medical virology*, e2285. doi:10.1002/rmv.2285

Walsh, S., Chowdhury, A., Braithwaite, V., Russell, S., Birch, J. M., Ward, J. L., . . . Mytton, O. T. (2021). Do school closures and school reopenings affect community transmission of COVID-19? A systematic review of observational studies. *BMJ open, 11*(8), e053371. doi:10.1136/bmjopen-2021-053371

Yaacoub, S., Khabsa, J., El-Khoury, R., El-Harakeh, A., Lotfi, T., Saad, Z., . . . Akl, E. A. (2021). COVID-19 transmission during swimming-related activities: a rapid systematic review. *BMC infectious diseases, 21*(1), 1112. doi:10.1186/s12879-021-06802-4

Yaghoubi, M., Salimi, M., & Meskarpour-Amiri, M. (2021). Systematic review of productivity loss among healthcare workers due to Covid-19. *The International journal of health planning and management*. doi:10.1002/hpm.3351

Zaccagni, L., Toselli, S., & Barbieri, D. (2021). Physical Activity during COVID-19 Lockdown in Italy: A Systematic Review. *International journal of environmental research and public health, 18*(12). doi:10.3390/ijerph18126416
