# Supplementary material for: The effects of public health and social measures (PHSM) implemented during the COVID‐19 pandemic: An overview of systematic reviews
Source: Cochrane Evid Synth Methods. 2024 Apr 29;2(5):e12055. doi: 10.1002/cesm.12055 (PMC11795948; doi:10.1002/cesm.12055)
Supplement: Supplementary file 3 — Appendix 3: Methodological assessment of included reviews. [file CESM-2-e12055-s001.pdf]

Appendix 3: AMSTAR rating for each included review

**Checklist Questions**	1. Did the research questions and inclusion criteria for the review include the components of PICO?
	2. Did the report of the review contain an explicit statement that the review methods were established prior to the conduct of the review and did the report justify any significant deviations from the protocol?
	3. Did the review authors explain their selection of the study designs for inclusion in the review?
	4. Did the review authors use a comprehensive literature search strategy?
	5.Did the review authors perform study selection in duplicate?
	6. Did the review authors perform data extraction in duplicate?
	7. Did the review authors provide a list of excluded studies and justify the exclusions?
	8. Did the review authors describe the included studies in adequate detail?
	9. Did the review author s use a satisfactory technique for assessing the risk of bias (RoB) in individual studies that were included in the review?
	10.Did the review authors report on the sources of funding for the studies included in the review?
	11. If meta- analysis was performed did the review authors use appropriate methods for statistical combination of results?
	12. If meta- analysis was performed, did the review authors assess the potential impact of RoB in individual studies on the results of the meta-analysis or other evidence synthesis?
	13. Did the review authors account for RoB in individual studies when interpreting/ discussing the results of the review?
	14. Did the review authors provide a satisfactory explanation for, and discussion of, any heterogeneity observed in the results of the review?
	15. If they performed quantitative synthesis did the review authors carry out an adequate investigation of publication bias (small study bias) and discus s its likely impact on the results of the review?
	16.Did the review authors report any potential sources of conflict of interest, including any funding they received for conducting the review?

Appendix 3: AMSTAR rating for each included review

AMSTAR 2 Checklist																	
**Author's Name**	**Q.1** PICO	**Q.2** Protocol	**Q.3** Study Design	**Q.4** Search Strategy	**Q.5** Study Selection	**Q.6** Data Extraction	**Q.7** Excluded studies	**Q.8** Included Studies	**Q.9** Risk of Bias	**Q.10** Funding Sources	**Q.11** Meta-analysis	**Q.12** Impact Risk of Bias	**Q.13** Discussing Risk of Bias	**Q.14** Heterogeneity	**Q.15** Publication Bias	**Q.16** Conflict of Interest	**Final AMSTAR SCORING**
Asín-Izquierdo et al., 2022	Yes	Partial Yes	No	No	Yes	No	No	Partial Yes	Partial Yes	No	No meta-analysis conducted	No meta-analysis conducted	Yes	No	No meta-analysis conducted	No	Critically low confidence
Della Valle P. G. et al. 2021	Yes	Partial Yes	No	Partial Yes	Yes	Yes	No	Yes	Yes	No	No meta-analysis conducted	No meta-analysis conducted	No	No	No meta-analysis conducted	Yes	Critically low confidence
Alex, R.P., 2021	Yes	No	Yes	Yes	No	No	No	Yes	No	No	Yes	No	No	No	Yes	No	Critically low confidence
Alkatout, 2021	Yes	No	No	Partial Yes	Yes	Yes	No	Partial Yes	Yes	No	No meta-analysis conducted	No meta-analysis conducted	No	No	No meta-analysis conducted	Yes	Critically low confidence
Antonio Silverii, 2021	Yes	Yes	No	Partial Yes	Yes	Yes	No	Partial Yes	Yes	No	Yes	No	No	Yes	Yes	Yes	Critically low confidence
B. Kunstler, 2022	Yes	Partial Yes	No	No	Yes	Yes	No	Yes	Yes	No	No	No	Yes	No	No	No	Critically low confidence
Bakaloudi, 2021	No	No	No	Partial Yes	Yes	Yes	No	Partial Yes	Yes	No	No meta-analysis conducted	No meta-analysis conducted	No	No	No meta-analysis conducted	Yes	Critically low confidence

Appendix 3: AMSTAR rating for each included review

Baumhardt, M., et al, 2021	Yes	Yes	No	Partial Yes	Yes	Yes	No	Partial Yes	Yes	No	Yes	Yes	Yes	Yes	Yes	Yes	Low confidence
Bou- Karroum, 2021	Yes	No	Yes	Yes	Yes	Yes	No	Yes	Yes	Yes	No meta-analysis conducted	No meta-analysis conducted	No	No	No meta-analysis conducted	Yes	Critically low confidence
Brakspear, 2022	Yes	Yes	No	Yes	Yes	Yes	No	Yes	Yes	No	No meta-analysis conducted	No meta-analysis conducted	Yes	No	No meta-analysis conducted	Yes	Low confidence
C. Mignogna, 2021	Yes	Partial Yes	No	Partial Yes	Yes	Yes	No	Partial Yes	Partial Yes	No	No meta-analysis conducted	No meta-analysis conducted	No	Yes	No meta-analysis conducted	Yes	Critically low confidence
Camacho-Montaño, 2022	Yes	No	No	Partial Yes	No	No	No	Partial Yes	Yes	No	No meta-analysis conducted	No meta-analysis conducted	Yes	No	No meta-analysis conducted	Yes	Critically low confidence
Cardwell, 2020	Yes	Yes	Yes	Partial Yes	No	No	No	Partial Yes	Partial Yes	No	No meta-analysis conducted	No meta-analysis conducted	No	No	No meta-analysis conducted	Yes	Critically low confidence
Caristia, S., et al., 2020	Yes	Partial Yes	Yes	Partial Yes	Yes	No	No	Partial Yes	No	No	No meta-analysis conducted	No meta-analysis conducted	Yes	Yes	No meta-analysis conducted	Yes	Critically low confidence
Cavicchioli M., et al., 2021	Yes	No	No	Partial Yes	No	Yes	No	Yes	Yes	No	No meta-analysis conducted	No meta-analysis conducted	Yes	No	No meta-analysis conducted	Yes	Critically low confidence

Appendix 3: AMSTAR rating for each included review

Chaabane, 2021	Yes	Yes	No	Partial Yes	No	No	No	Yes	No	No	No meta-analysis conducted	No meta-analysis conducted	No	No	No meta-analysis conducted	Yes	Critically low confidence
Chai J., et al., 2021	Yes	Yes	No	Yes	No	No	No	Yes	Yes	No	Yes	Yes	Yes	Yes	Yes	Yes	Low confidence
D. R. Bakaloudi, 2021	Yes	Partial Yes	No	No	Yes	Yes	No	No	Yes	No	Yes	Yes	Yes	Yes	Yes	Yes	Critically low confidence
Daniela, R, 2020	Yes	Partial Yes	Yes	Partial Yes	No	No	No	Partial Yes	Partial Yes	No	No meta-analysis conducted	No meta-analysis conducted	No	Yes	No meta-analysis conducted	Yes	Critically low confidence
Desye, 2021	No	No	No	Partial Yes	No	Yes	No	No	Partial Yes	No	No meta-analysis conducted	No meta-analysis conducted	No	No	No meta-analysis conducted	Yes	Critically low confidence
Elisabeth A. L., et al., 2021	Yes	Partial Yes	Yes	Partial Yes	No	Yes	No	Yes	Yes	No	No meta-analysis conducted	No meta-analysis conducted	Yes	No	No meta-analysis conducted	No	Critically low confidence
Farooq et al., 2021	No	Partial Yes	No	Partial Yes	Yes	Yes	No	No	No	No	Yes	No	No	Yes	No	Yes	Critically low confidence

Appendix 3: AMSTAR rating for each included review

Freiberg et al., 2021	Yes	Partial Yes	Yes	No	No	No	Yes	Partial Yes	Partial Yes	Yes	No meta-analysis conducted	No meta-analysis conducted	Yes	No	No meta-analysis conducted	Yes	Critically low confidence
Garofolo, 2021	Yes	Yes	No	Partial Yes	Yes	Yes	No	Yes	Yes	No	Yes	No	No	No	Yes	Yes	Critically low confidence
Girum, 2020	Yes	Partial Yes	Yes	Yes	Yes	No	Partial Yes	Partial Yes	Yes	No	No meta-analysis conducted	No meta-analysis conducted	No	No	No meta-analysis conducted	Yes	Critically low confidence
Grekousis G. and Y. Liu, 2021	Yes	No	No	Partial Yes	Yes	No	No	Yes	Yes	No	No meta-analysis conducted	No meta-analysis conducted	No	No	No meta-analysis conducted	Yes	Critically low confidence
Grépin, 2021	Yes	Partial Yes	No	Partial Yes	Yes	Yes	No	Yes	Partial Yes	No	No meta-analysis conducted	No meta-analysis conducted	No	No	No meta-analysis conducted	Yes	Critically low confidence
Hammerstein, 2021	Yes	No	No	Partial Yes	Yes	No	No	No	Yes	No	No meta-analysis conducted	No meta-analysis conducted	Yes	No	No meta-analysis conducted	Yes	Critically low confidence
Hatami, 2022	Yes	Yes	No	Partial Yes	Yes	Yes	No	Partial Yes	Yes	No	No meta-analysis conducted	No meta-analysis conducted	No	No	No meta-analysis conducted	Yes	Critically low confidence

Appendix 3: AMSTAR rating for each included review

Hossain A. D., et al. 2022	Yes	Partial Yes	No	Yes	Yes	Yes	No	Yes	Yes	No	No meta-analysis conducted	No meta-analysis conducted	Yes	Yes	No meta-analysis conducted	Yes	Low confidence
Hugelius, 2021	Yes	No	No	Partial Yes	Yes	Yes	No	Yes	Yes	No	No meta-analysis conducted	No meta-analysis conducted	Yes	No	No meta-analysis conducted	Yes	Critically low confidence
Iezadi, 2021	Yes	Yes	No	Yes	Yes	Yes	No	Yes	Yes	No	Yes	Yes	Yes	Yes	Yes	Yes	Low confidence
Imen Ayouni, 2021	Yes	Partial Yes	No	Partial Yes	Yes	Yes	No	Partial Yes	Yes	No	No meta-analysis conducted	No meta-analysis conducted	No	No	No meta-analysis conducted	Yes	Critically low confidence
Ingram C. et al., 2021	Yes	No	No	Partial Yes	Yes	Yes	No	Yes	Yes	No	Yes	No	Yes	Yes	Yes	Yes	Critically low confidence
J. Burns, 2021	Yes	Yes	Yes	Yes	Yes	No	Yes	Yes	Yes	Yes	No meta-analysis conducted	No meta-analysis conducted	Yes	Yes	No meta-analysis conducted	Yes	High confidence
J. M. Stratil, 2021	Yes	Yes	Yes	Yes	Yes	No	Yes	Yes	Yes	Yes	No meta-analysis conducted	No meta-analysis conducted	Yes	Yes	No meta-analysis conducted	Yes	High confidence

Appendix 3: AMSTAR rating for each included review

Jabs at al., 2022	Yes	Partial Yes	Yes	Partial Yes	Yes	Yes	No	No	Partial Yes	No	Yes	Yes	Yes	Yes	No	Yes	Critically low confidence
Jenniskens K., et al., 2021	Yes	No	No	Partial Yes	Yes	Yes	No	Yes	Yes	No	No meta-analysis conducted	No meta-analysis conducted	Yes	No	No meta-analysis conducted	Yes	Critically low confidence
João M. Castaldelli-Maia, 2021	No	No	No	Partial Yes	Yes	Yes	Yes	Partial Yes	Partial Yes	No	Yes	No	Yes	No	No	Yes	Critically low confidence
Kharel, 2022	Yes	Yes	Yes	Yes	Yes	Yes	Yes	Yes	Yes	No	No meta-analysis conducted	No meta-analysis conducted	No	No	No meta-analysis conducted	Yes	Low confidence
Khatib et al., 2022	Yes	Partial Yes	No	No	No	Yes	Yes	No	No	Yes	No meta-analysis conducted	No meta-analysis conducted	No	Yes	No meta-analysis conducted	Yes	Critically low confidence
Khosravizad eh et al., 2022	No	No	No	No	Yes	Yes	No	No	No	No	No meta-analysis conducted	No meta-analysis conducted	No	No	No meta-analysis conducted	No	Critically low confidence
Kourti A., et al., 2021	Yes	No	No	Partial Yes	Yes	Yes	No	Yes	Yes	No	No meta-analysis conducted	No meta-analysis conducted	No	No	No meta-analysis conducted	Yes	Critically low confidence

Appendix 3: AMSTAR rating for each included review

Kourti et al., 2021	Yes	Partial Yes	No	No	Yes	No	No	Yes	Partial Yes	No	No meta-analysis conducted	No meta-analysis conducted	Yes	No	No meta-analysis conducted	Yes	Critically low confidence
L. M. Fricke, 2020	No	Partial Yes	No	No	No	No	No	No	Partial Yes	No	No meta-analysis conducted	No meta-analysis conducted	No	Yes	No meta-analysis conducted	Yes	Critically low confidence
Lausi, 2021	No	Yes	No	Partial Yes	Yes	Yes	No	Partial Yes	Yes	No	No meta-analysis conducted	No meta-analysis conducted	No	No	No meta-analysis conducted	Yes	Critically low confidence
M. Bonati, 2022	Yes	No	No	Partial Yes	No	No	No	No	No	No	No meta-analysis conducted	No meta-analysis conducted	No	Yes	No meta-analysis conducted	Yes	Critically low confidence
M. Mbwogge, 2021	Yes	No	No	No	No	No	Yes	No	Partial Yes	No	No meta-analysis conducted	No meta-analysis conducted	Yes	Yes	No meta-analysis conducted	No	Critically low confidence
Mendez- Brito A., et al., 2021	Yes	No	No	Partial Yes	No	No	No	Partial Yes	Partial Yes	No	No meta-analysis conducted	No meta-analysis conducted	Yes	Yes	No meta-analysis conducted	Yes	Critically low confidence
Minozzi, 2021	Yes	Partial Yes	No	Partial Yes	Yes	Yes	No	Yes	Yes	No	No meta-analysis conducted	No meta-analysis conducted	Yes	No	No meta-analysis conducted	Yes	Critically low confidence

Appendix 3: AMSTAR rating for each included review

N. Ford, 2021	Yes	Partial Yes	No	Partial Yes	No	No	No	Partial Yes	Yes	No	No meta-analysis conducted	No meta-analysis conducted	No	No	No meta-analysis conducted	Yes	Critically low confidence
N. Johanna, 2020	Yes	Partial Yes	No	Partial Yes	Yes	Yes	No	Partial Yes	Yes	No	No meta-analysis conducted	No meta-analysis conducted	No	Yes	No meta-analysis conducted	Yes	Critically low confidence
Neira et al., 2021	No	Partial Yes	No	No	Yes	Yes	No	No	Partial Yes	No	No meta-analysis conducted	No meta-analysis conducted	No	No	No meta-analysis conducted	Yes	Critically low confidence
Nussbaumer-Streit et al., 2020	Yes	Partial Yes	Yes	Partial Yes	Yes	No	Partial Yes	Partial Yes	Partial Yes	No	No meta-analysis conducted	No meta-analysis conducted	Yes	Yes	No meta-analysis conducted	Yes	Critically low confidence
O. Byambasuren, 2021	Yes	Partial Yes	No	Partial Yes	Yes	Yes	Yes	Partial Yes	Partial Yes	No	No meta-analysis conducted	No meta-analysis conducted	Yes	Yes	No meta-analysis conducted	Yes	Low confidence
Oliveira, 2021	Yes	Partial Yes	No	Partial Yes	No	No	No	Partial Yes	No	No	Yes	Yes	No	Yes	Yes	Yes	Critically low confidence
P. Galanis, 2021	Yes	Partial Yes	No	No	Yes	Yes	No	Partial Yes	Partial Yes	No	Yes	Yes	No	Yes	Yes	Yes	Critically low confidence

Appendix 3: AMSTAR rating for each included review

Panchal, 2021	Yes	Yes	No	Yes	No	Yes	No	Partial Yes	Yes	No	No meta-analysis conducted	No meta-analysis conducted	No	No	No meta-analysis conducted	No	Critically low confidence
Panda et al., 2020	Yes	Partial Yes	No	Partial Yes	Yes	Yes	No	No	Partial Yes	No	Yes	No	Yes	No	Yes	No	Critically low confidence
Pizarro A. B., et al., 2022	Yes	Yes	Yes	Yes	Yes	Yes	Yes	Yes	Yes	Yes	No meta-analysis conducted	No meta-analysis conducted	Yes	Yes	No meta-analysis conducted	Yes	High confidence
Qathrin, 2021	Yes	No	No	Partial Yes	No	No	No	Yes	Yes	No	No meta-analysis conducted	No meta-analysis conducted	No	No	No meta-analysis conducted	Yes	Critically low confidence
R. L. Knight, 2021	Yes	No	No	No	Yes	No	No	Yes	No	No	No meta-analysis conducted	No meta-analysis conducted	No	No	No meta-analysis conducted	Yes	Critically low confidence
Rajkumar et al., 2022	Yes	Partial Yes	No	No	Yes	Yes	No	No	Partial Yes	No	No meta-analysis conducted	No meta-analysis conducted	No	No	No meta-analysis conducted	Yes	Critically low confidence
Rajmil, 2021	Yes	No	No	Partial Yes	Yes	No	No	Partial Yes	Yes	Yes	No meta-analysis conducted	No meta-analysis conducted	Yes	No	No meta-analysis conducted	Yes	Critically low confidence

Appendix 3: AMSTAR rating for each included review

Regmi, 2021	Yes	Yes	Yes	Yes	Yes	Yes	No	Partial Yes	Yes	No	No meta-analysis conducted	No meta-analysis conducted	No	Yes	No meta-analysis conducted	Yes	Critically low confidence
Rezwanul et al., 2021	No	Partial Yes	Yes	Partial Yes	Yes	Yes	No	No	No	Yes	No meta-analysis conducted	No meta-analysis conducted	No	No	No meta-analysis conducted	Yes	Critically low confidence
Rivera et al., 2021	Yes	Partial Yes	No	Partial Yes	Yes	Yes	Yes	Yes	Yes	No	No meta-analysis conducted	No meta-analysis conducted	Yes	No	No meta-analysis conducted	Yes	Low confidence
Rodríguez-Fernández et al., 2021	Yes	Partial Yes	Yes	No	Yes	Yes	No	Partial Yes	Partial Yes	No	No meta-analysis conducted	No meta-analysis conducted	Yes	No	No meta-analysis conducted	Yes	Critically low confidence
Runacres A., et al., 2021	Yes	Yes	No	Partial Yes	Yes	No	No	Yes	Yes	No	Yes	Yes	Yes	No	No	Yes	Low confidence
S. Hawco, 2022	Yes	Yes	No	No	Yes	Yes	Partial Yes	No	Partial Yes	No	Yes	Yes	Yes	Yes	Yes	Yes	Critically low confidence
S. Krishnaratne, 2022	Yes	Yes	Yes	Partial Yes	Yes	Yes	Yes	Yes	Yes	Yes	No meta-analysis conducted	No meta-analysis conducted	Yes	Yes	No meta-analysis conducted	Yes	High confidence

Appendix 3: AMSTAR rating for each included review

Samji, 2022	Yes	Partial Yes	No	No	Yes	Yes	No	Partial Yes	No	No	No meta-analysis conducted	No meta-analysis conducted	No	No	No meta-analysis conducted	Yes	Critically low confidence
Saulle 2021	Yes	Partial Yes	No	Partial Yes	Yes	Yes	No	Yes	Yes	No	No meta-analysis conducted	No meta-analysis conducted	Yes	No	No meta-analysis conducted	Yes	Critically low confidence
Schmidt R. A., et al., 2021	Yes	No	No	Partial Yes	Yes	No	No	Yes	Yes	No	No meta-analysis conducted	No meta-analysis conducted	No	No	No meta-analysis conducted	Yes	Critically low confidence
Shekaraiah 2021	Yes	Partial Yes	No	Partial Yes	Yes	No	No	Yes	Yes	No	No meta-analysis conducted	No meta-analysis conducted	Yes	No	No meta-analysis conducted	Yes	Critically low confidence
Sideli, 2021	Yes	No	No	Partial Yes	No	Yes	No	No	Yes	No	Yes	No	Yes	Yes	Yes	Yes	Critically low confidence
Sohi et al., 2022	No	Partial Yes	No	Partial Yes	No	Yes	No	Yes	Yes	No	No meta-analysis conducted	No meta-analysis conducted	No	Yes	No meta-analysis conducted	Yes	Critically low confidence
Stella Talic, 2021	Yes	Yes	No	Partial Yes	Yes	Yes	No	Partial Yes	Yes	No	Yes	Yes	Yes	Yes	No	Yes	Critically low confidence

Appendix 3: AMSTAR rating for each included review

Stephanie, 2021	Yes	Yes	Yes	Partial Yes	Yes	Yes	Yes	Yes	Yes	No	No meta-analysis conducted	No meta-analysis conducted	No	Yes	No meta-analysis conducted	Yes	Low confidence
Suárez- González 2021	Yes	Yes	Yes	Partial Yes	Yes	Yes	No	Yes	Yes	No	No meta-analysis conducted	No meta-analysis conducted	Yes	Yes	No meta-analysis conducted	Yes	Low confidence
T. Girum, 2021	Yes	Partial Yes	Yes	No	Yes	Yes	No	Yes	Yes	No	No meta-analysis conducted	No meta-analysis conducted	Yes	No	No meta-analysis conducted	Yes	Critically low confidence
Tabatabaeizadeh 2021	Yes	Partial Yes	No	Partial Yes	No	No	No	Partial Yes	Yes	No	Yes	No	Yes	Yes	Yes	Yes	Critically low confidence
Tu-Hsuan Chang, 2021	Yes	No	No	Partial Yes	Yes	Yes	No	Yes	Yes	Yes	Yes	No	No	Yes	Yes	Yes	Critically low confidence
Tully 2021	Yes	Partial Yes	No	Partial Yes	Yes	Yes	No	Partial Yes	Yes	No	No meta-analysis conducted	No meta-analysis conducted	Yes	No	No meta-analysis conducted	Yes	Critically low confidence
Viner et al., 2022	No	Partial Yes	No	No	Yes	Yes	No	No	Partial Yes	No	No meta-analysis conducted	No meta-analysis conducted	No	No	No meta-analysis conducted	Yes	Critically low confidence
Viswanathan 2020	Yes	Yes	Yes	Yes	Yes	Yes	Partial Yes	Yes	Yes	Yes	No meta-analysis conducted	No meta-analysis conducted	Yes	Yes	No meta-analysis conducted	Yes	Moderate confidence

Appendix 3: AMSTAR rating for each included review

Wall, 2022	Yes	Yes	No	Partial Yes	Yes	No	No	Partial Yes	Yes	No	No meta-analysis conducted	No meta-analysis conducted	Yes	Yes	No meta-analysis conducted	No	Low confidence
Walsh, 2021	Yes	Yes	No	Partial Yes	No	No	Yes	Yes	Yes	Yes	No meta-analysis conducted	No meta-analysis conducted	Yes	No	No meta-analysis conducted	Yes	Moderate confidence
Walsh, 2022	Yes	Yes	No	Partial Yes	No	No	Yes	Partial Yes	Yes	No	No meta-analysis conducted	No meta-analysis conducted	Yes	No	No meta-analysis conducted	Yes	Moderate confidence
Walsh, S. 2021	Yes	Yes	Yes	Partial Yes	Yes	Yes	No	Yes	Yes	No	No meta-analysis conducted	No meta-analysis conducted	Yes	Yes	No meta-analysis conducted	Yes	Low confidence
Y. Jin, 2021	Yes	No	Yes	No	Yes	No	No	No	No	No	Yes	No	No	Yes	Yes	Yes	Critically low confidence
Yaacoub, S. 2021	Yes	Yes	No	Partial Yes	Yes	Yes	Yes	Yes	Yes	No	No meta-analysis conducted	No meta-analysis conducted	Yes	No	No meta-analysis conducted	Yes	Moderate confidence
Yaghoubi et al., 2021	No	No	No	Partial Yes	Yes	Yes	No	No	No	No	No meta-analysis conducted	No meta-analysis conducted	No	No	No meta-analysis conducted	Yes	Critically low confidence
Zaccagni et al., 2021	Yes	Partial Yes	No	No	Yes	No	Partial Yes	Yes	Partial Yes	No	No meta-analysis conducted	No meta-analysis conducted	No	No	No meta-analysis conducted	Yes	Critically low confidence
