# Supplementary material for: The effects of public health and social measures (PHSM) implemented during the COVID‐19 pandemic: An overview of systematic reviews
Source: Cochrane Evid Synth Methods. 2024 Apr 29;2(5):e12055. doi: 10.1002/cesm.12055 (PMC11795948; doi:10.1002/cesm.12055)
Supplement: Supplementary file 4 — Appendix 4: Detailed findings from all reviews stratified by intervention and outcome categories. [file CESM-2-e12055-s002.pdf]

Appendix 4: Detailed findings from all reviews- stratified by intervention and outcome categories

Below, we summarize the key findings narratively and in tabular formats, categorized by the following intervention categories:

- Active Surveillance
- Response
- Services
- Social interactions
- Movement
- Physical environment
- Individual protection
- Multicomponent interventions
- Lockdown

Within each intervention category, we start by summarizing the characteristics of the included reviews followed by the reported outcomes for the different comparisons. Given that most of the included reviews were assessed as having important methodological limitations (with generally low certainty of evidence where this was assessed using GRADE), it was challenging to report the results according to the best available review.

In the tables, we reproduce the findings as written by the authors of those reviews. However, in the narrative text, we applied the GRADE guidance on informative statements (Santesso et al., 2020). For reviews that had major methodological limitations and had not applied GRADE, we used GRADE guidance on informative statements as applicable to ‘low’ certainty.

CONFIDENTIAL

a. Active Surveillance (n=14 reviews)

Fourteen reviews (7 systematic reviews and 7 rapid reviews) examined the association between active surveillance and COVID-19 epidemiological outcomes (Burns et al., 2021, Cardwell et al., 2021, Grekousis and Liu, 2021, Hossain et al., 2022, Ingram et al., 2021, Jabs et al., 2022, Jenniskens et al., 2021, Johanna et al., 2020, Pizarro et al., 2022, Stratil et al., 2021, Talic et al., 2021, Viswanathan et al., 2020, Walsh et al., 2022, Mbwogge, 2021).

Below, we present the findings by comparisons.

i. Screening compared with no screening (n=5 reviews)

We included five reviews on the use of screening (compared to no screening); none with a search date after 2021 and with one rated as high, two rated as moderate, and two as critically low confidence on the AMSTAR 2 instrument (see table 1) (Burns et al., 2021, Cardwell et al., 2021, Talic et al., 2021, Viswanathan et al., 2020, Walsh et al., 2022). Two of the reviews applied GRADE.

All reviews examined COVID-19 epidemiological outcomes (see table 2). The reviews with high or moderate confidence on the AMSTAR 2 instrument found the following: Symptom/exposure-based screening at borders probably reduces the number or proportion of exported cases and may also reduce the risk of outbreak and days to outbreak of COVID-19 in unaffected populations (Burns et al., 2021). It is uncertain whether test-based screening at borders increases or decreases the proportion of secondary or imported cases (Burns et al., 2021) (Viswanathan et al., 2020). It is also uncertain whether laboratory screening of healthcare workers reduce COVID-19 transmission to patients and other healthcareworkers at an emergency department (Viswanathan et al., 2020). There is also uncertainty surrounding the effectiveness of rapid antigen testing in screening asymptomatic individuals (population-level screening, pre-event screening and serial testing) to mitigate the transmission of COVID-19 pandemic (Walsh et al., 2022).

Findings from the two reviews with critically low confidence on the AMSTAR 2 instrument suggest that thermal screening or screening for fever may lack sensitivity in detecting people with COVID-19 infection (Talic et al., 2021) and thus may be ineffective in restraining the spread of COVID-19 pandemic (Cardwell et al 2020)

CONFIDENTIAL

Table 1: Characteristics of reviews

**Review *(rapid status in brackets)***	**Type of intervention**	**Population; Setting**	**# of studies included *(#RCT in bracket)***	**AMSTAR rating**	**Date last searched**	**Meta-analysis conducted**
(Viswanathan et al., 2020) (Rapid review)	Symptom screening and Laboratory test screening	General population; Point of entry and healthcare facility	2	Moderate	May, 2020	No
(Burns et al., 2021) (Rapid review)	Symptom/exposure-based screening or test-based screening (commonly specifying polymerase chain reaction (PCR) testing), or both	General population; point of entry	27	High	November, 2020	No
(Walsh et al., 2022) (Rapid review)	Rapid antigen testing for the screening	General population; Healthcare, Entertainment, Educational and community	16 (1 RCT)	Moderate	Not specified; 2021	No

		setting (Prison)				
(Cardwell et al., 2021) (Rapid review)	Non-contact thermal screening	General population; Point of entry	3	Critically low	October 07, 2020	No
(Talic et al., 2021)	Screening for fever	General population; Point of entry	1	Critically low	Not specified; 2021	Yes

Table 2: COVID-19 epidemiological outcomes

**Review**	**Risk and incidence**	**Transmission-related outcomes**
(Viswanathan et al., 2020)<br>GRADE: Yes; 1 low and 1 very low certainty of evidence	It is uncertain whether screening of healthcare workers in emergency departments using laboratory tests reduces transmission to patients and other healthcare workers (assuming a transmission constant of 1.2 new infections per 10,000 people, weekly screening reduced infections by 5.1% within 30 days) (1 modelling study)	Symptom screening at travel hubs such as airports, may slightly slow but not stop the importation of infected cases (assuming 10 or 100 infected travellers per week reduced the delay in a local outbreak to 8 days or 1 day) (1 modelling study)
(Burns et al., 2021)<br>GRADE: Yes; very low to moderate certainty of evidence	Symptom/exposure-based screening at borders, probably reduces the number of cases exported per day from another country by 82% (95% CI: 72%-95%) (one modelling study). Proportion of cases detected may range from 1% to 53% for modelling studies and from 0% to 100% for observational studies , although all but one study observed this proportion to be less than 54%.	It is uncertain whether symptom/exposure-based screening at borders results in delay in epidemic development (four modelling studies)<br><br>It is uncertain whether ; PCR testing at borders as a screening measure reduces imported or exported cases as well as secondary cases (one modelling study). Proportion of cases detected may vary from 58% to 90% (five observational studies)
(Walsh et al., 2022)<br>GRADE: No		There is uncertainty surrounding the effectiveness of rapid antigen testing in screening asymptomatic individuals to mitigate the transmission of COVID-19
(Cardwell et al., 2021)<br>GRADE: No		Thermal screening may be ineffective in restraining the spread of COVID-19 due to the presence of asymptomatic or pre-symptomatic cases.

CONFIDENTIAL

(Talic et al., 2021)<br>GRADE: No	Fever screening may lack sensitivity (ranging from 18% to 24%) for detecting people with COVID-19 infection (based on one included study).	

ii. *Testing compared with no testing (n=4 reviews)*

We included four reviews on the use of testing (compared to no testing) none with a search date after 2021(Stratil et al., 2021, Johanna et al., 2020, Jabs et al., 2022) (table 3). One of the reviews was rated as high confidence on the AMSTAR instrument (Stratil et al., 2021) while the other three were rated as critically low (Johanna et al., 2020, Jabs et al., 2022). Among the three reviews, two applied GRADE (Stratil et al., 2021, Mbwogge, 2021).

All reviews examined COVID-19 epidemiological outcomes (table 4). The reviews with high confidence on the AMSTAR 2 instrument found the following: routine testing of residents and staff independent of symptoms may reduce the number of infections. Evidence from one observational study suggests that the measure may reduce, while the evidence from one modelling study suggests that it probably reduces hospitalisations. The measure may also reduce the number of deaths among residents, but it is uncertain whether it reduces deaths among staff or whether it increases or decreases probability of outbreaks. It is uncertain whether symptom-based surveillance testing in long-term care facilities reduces infections and lowers the likelihood of outbreaks, as well as the number of deaths among residents (Stratil et al., 2021).

Findings from two reviews with critically low confidence on the AMSTAR 2 instrument suggest that PCR testing, whether in healthcare setting or a part of mass testing, may reduce risk and incidence as well as mortality from COVID-19 (Jabs et al., 2022, Johanna et al., 2020); however, one of the reviews reported insufficient evidence to determine the effectiveness of mass testing in isolation without other accompanying measures such as lockdown (Johanna et al 2020). The third critically low review suggested that the efficacy of mass testing might surpass that of the conventional testing strategy in curbing the transmission of COVID-19 within community settings (Mbwogge, 2021).

CONFIDENTIAL

Table 3: Characteristics of reviews

**Review** ***(rapid status in brackets)***	**Type of intervention**	**Population; Setting**	**# of studies included** ***(#RCT in bracket)***	**AMSTAR Rating**	**Date last searched**	**Meta-analysis conducted**

(Stratil et al., 2021) (Rapid review)	Routine testing; PCR Testing and antigen-based approaches	Adult; long-term care setting	8	High	Not specified; 2021	No
(Johanna et al., 2020)	Mass testing RT-PCR testing	General population; Not specified	1	Critically low	June, 2020	No
(Jabs et al., 2022)	PCR testing	Healthcare workers; Healthcare setting	39	Critically low	May 04, 2021	Yes
(Mbwogge, 2021)	Mass testing and contact tracing versus conventional test and trace method	General population; Community setting	12	Critically low	December, 2020	Yes

Table 4: COVID-19 epidemiological outcomes

**Review**	**Risk and incidence**	**Transmission-related outcomes**	**Mortality**	**Hospitalizations**
(Stratil et al., 2021) GRADE: Yes; very low to moderate certainty evidence	Routine testing of both residents and staff, regardless of symptoms may reduce the number of infections<br><br>It is uncertain whether symptom-based surveillance testing reduces the number of infections<br>It is uncertain whether testing of new admissions or intensified testing of re-admissions of residents and staff after holidays increases or decreases the number of infections	It is uncertain whether routine testing of both residents and staff lowers the likelihood of outbreaks<br>It is uncertain whether symptom-based surveillance testing lowers the likelihood of outbreaks	Routine testing of residents may reduce the number of deaths among residents but the evidence on deaths among staff is unclear	Evidence from one observational study suggests that routine testing of both residents and staff may reduce, while the evidence from one modelling study suggests that it probably reduces hospitalization
(Johanna et al., 2020) GRADE: No	Mass testing may reduce the total number of infected people (based on one modelling study)<br><br>Combining mass testing with lock		Mass testing may a significantly reduce mortality rates<br>Combining mass testing with lock down measures may result in a greater reduction of mortality rate.	

CONFIDENTIAL

	down measures may result in a greater reduction of incidence			
(Jabs et al., 2022) GRADE: No	PCR testing may reduce risk and incidence of COVID-19. 1.9% of asymptomatic healthcare workers tested positive for SARS-CoV-2 using PCR testing. The proportion of positive test results ranged between 0 and 14.3%.			
(Mbwogge, 2021) GRADE: Yes; very low		Mass testing may be more effective than the conventional test strategy in reducing COVID-19 transmission in community settings		

iii. Test-based attendance in workplace compared with self-isolation (n=1 review)

We included one review on the use of testing (compared to self-isolation) with a search date of 2021, and rated as high confidence on the AMSTAR 2 instrument (Pizarro et al., 2022) (table 5). The review suggests that it is unclear whether a test-based attendance policy affects rates of PCR-positive COVID-19 infection compared to standard 10-day self-isolation amongst school and college staff (Pizarro et al., 2022). In addition, the test-based attendance policy may result in little to no difference in absence rates compared to standard 10-day self-isolation. The review applied GRADE and the outcome assessed was very low certainty evidence (tables 6 and 7).

Table 5: Characteristics of reviews

**Review *(rapid status in brackets)***	**Type of intervention**	**Population; Setting**	**# of studies included *(#RCT in bracket)***	**AMSTAR rating**	**Date last searched**	**Meta-analysis conducted**
(Pizarro et al., 2022)	Test-based attendance and standard 10-day self-isolation	Adults; Occupational setting	1 (1 RCT)	High	September, 2021	No

Table 6: COVID-19 epidemiological outcomes

**Review**	Risk and incidence

CONFIDENTIAL

(Pizarro et al., 2022)<br>GRADE: Yes; very low certainty evidence	The results between test-based attendance and standard 10-day self-isolation were inconclusive for the rate of symptomatic PCR-positive SARS-COV-2 infection rate ratio ((RR) 1.28, 95% confidence interval (CI) 0.74 to 2.21.

Table 7: Unintended health and socio-economic outcomes

**Review**	**Non-COVID-19 related health outcomes**
	**Absenteeism**
(Pizarro et al., 2022)<br><br>GRADE: Yes; very low certainty evidence	COVID-related absenteeism rates were 3704 absence days in 566,502 days-at-risk (6.5 per 1000 days at risk) in the control group and 2932 per 539,805 days-at-risk (5.4 per 1000 days at risk) in the intervention group (RR 0.83; 95% CI 0.55 to 1.25). Test-based attendance policy may result in little to no difference in absence rates compared to standard 10-day self-isolation.

iv. Contact tracing compared to contact tracing (n=3 reviews)

We included three reviews on the use of contact tracing (compared to no contact tracing); none with a search date after 2021, and with one rated as low and two as critically low confidence on the AMSTAR 2 instrument (table 8) (Hossain et al., 2022, Grekousis and Liu, 2021, Jenniskens et al., 2021). None of the reviews applied GRADE.

The findings from the three reviews suggest that contact tracing may reduce the risk, incidence, COVID-19 case growth rate and reproductive number (Rt) and mortality related to COVID-19 pandemic (table 9) (Grekousis and Liu, 2021, Hossain et al., 2022, Jenniskens et al., 2021).

Table 8: Characteristics of reviews

**Review *(rapid status in brackets)***	**Type of intervention**	**Population; Setting**	**# of studies included *(#RCT in bracket)***	**AMSTAR rating**	**Date last searched**	**Meta-analysis conducted**
(Hossain et al., 2022)	Wide range of tracing strategies (including phone calls, text messaging, and household contact tracing and personnel involved with the contact tracing (ranging from public health specialists to volunteers)	Not specified;	47	low	Not specified; 2021	No

(Grekousis and Liu, 2021)	Digital contact tracing	Not specified; Not specified	19	Critically low	March 31, 2021	No
(Jenniskens et al., 2021) (Rapid review)	Contact tracing apps (CTAs)	General population; Not limited to a setting	17	Critically low	Not specified; 2020	No

Table 9: COVID-19 epidemiological outcomes

**Review**	**Risk and incidence**	**Transmission-related outcomes**	**Mortality**
(Hossain et al., 2022) GRADE: No	Provider-led contact tracing efforts may result in a decrease in cases	Implementation of traditional contact tracing alongside digital contact tracing may reduce the basic reproduction number (R0) or Rt	
(Grekousis and Liu, 2021) GRADE: No		Digital contact tracing may reduce the effective reproduction number and the number of infected cases.	
(Jenniskens et al., 2021) GRADE: No	Contact tracing applications (CTAs) may reduce the total number of infections. An increased reduction may be observed at higher adoption rates of CTAs.	Contact tracing applications (CTAs) may reduce Rt.	Contact tracing applications (CTAs) may reduce mortality rates.

v. Asymptomatic PCR testing in workplace compared with combined IPC (n=1 review)

We included one rapid review on the use of asymptomatic PCR compared to combined infection prevention and control measures (IPC) and rated as critically low confidence on the AMSTAR instrument (table 10) (Ingram et al., 2021). The review did not apply GRADE (Ingram et al., 2021). Findings suggest that combined IPC may reduce employee COVID-19 positivity rates (0.2% positivity; 95% CI 0–0.4%) compared to asymptomatic PCR testing (1.7%; 95% CI 0.9–2.9%) (table 13). (Ingram et al., 2021) (Table 11).

Table 10: Characteristics of reviews

**Review *(rapid status in brackets)***	**Type of intervention**	**Population & Setting**	**Number of studies included *(#RCT in bracket)***	**AMSTAR rating**	**Date last searched**	**Meta-analysis conducted**
(Ingram et al., 2021) (Rapid Review)	Asymptomatic testing was carried out in a number of ways: universally, on a voluntary basis, following an outbreak,	Not Specified; Healthcare and occupational setting	33	Critically low	2020	Yes

CONFIDENTIAL

	according to environmental surface testing or contact tracing and during point prevalence surveys in hospitals and nursing homes<br><br>IPC measures comprised surveillance, outbreak investigation and response, PPE, changes in work arrangements, and worker education					

Table 11: COVID-19 epidemiological outcomes

**Review**	**Transmission-related outcomes**
(Ingram et al., 2021)<br>GRADE: No	A reduction in employee COVID-19 positivity rates (0.2% positivity; 95% CI 0–0.4%) as a result of combined IPC measures compared to asymptomatic PCR testing (1.7%; 95% CI 0.9–2.9%).

b. Response measures (n=15 reviews)

Fifteen reviews (11 systematic reviews and four rapid reviews) examined the association between response measures and COVID-19 epidemiological outcomes(n=7) as well as non-COVID-19 related health outcomes (n=8) (Ayouni et al., 2021, Bonati et al., 2022, Burns et al., 2021, Cavicchioli et al., 2021, Chai et al., 2021, Girum et al., 2020, Jin et al., 2021, Nussbaumer-Streit et al., 2020, Pizarro et al., 2022, Qathrin et al., 2021, Rajkumar et al., 2022, Schmidt et al., 2021, Stratil et al., 2021, Talic et al., 2021, Yaghoubi et al., 2021, Krishnaratne et al., 2022b) .

Below, we present the findings by comparison:

i. Quarantine compared with no quarantine (n=11 reviews)

We included 11 reviews on the use of quarantine (compared to no quarantine); none with a search date after 2021 and two rated as high, one as low and eight as critically low confidence on the AMSTAR instrument (table 12) (Bonati et al., 2022, Burns et al., 2021, Cavicchioli et al., 2021, Chai et al., 2021, Girum et al., 2020, Jin et al., 2021, Qathrin et al., 2021, Rajkumar et al., 2022, Schmidt et al., 2021, Stratil et al., 2021, Yaghoubi et al., 2021) . Among the 11 reviews, three applied GRADE (Girum et al., 2020, Stratil et al., 2021, Burns et al., 2021).

CONFIDENTIAL

In terms of COVID-19 epidemiological outcomes, the reviews with high confidence on the AMSTAR 2 instrument found the following (both of which applied GRADE): Quarantine for new admissions may reduce the number of infections in long-term care facilities (Stratil et al., 2021). Quarantine at borders may delay time to outbreak but it is uncertain whether it increases or decreases the number or proportion of cases or the proportion of imported cases. Differences in effects may depend on how long people were quarantined for and how well they followed the rules (Burns et al 2021). Findings from the reviews with low or critically low confidence on the AMSTAR 2 instrument suggest that quarantine implemented as self-quarantine and group quarantine may reduce the incidence of infection, shorten the duration of the epidemic and avert deaths. That review also found that implementation of early quarantine measures may make the strategy a more cost effective one, while integration of quarantine with other public health measures may increase the effectiveness and efficiency of the program (table 13) (Girum et al., 2020).

In terms of non-COVID-19 related health outcomes, these were examined by eight reviews with low or very low confidence on the AMSTAR 2 instrument. Findings from these reviews suggest that quarantine may be associated with psychological issues including stress, anxiety, post-traumatic stress disorder (PTSD), and disruptions in sleep patterns affecting both children and adults (Bonati et al., 2022, Cavicchioli et al., 2021, Chai et al., 2021, Jin et al., 2021, Qathrin et al., 2021, Rajkumar et al., 2022, Yaghoubi et al., 2021) as well as increase in alcohol use in certain segments of the population (table 14) (Schmidt et al., 2021). Findings from the meta-analysis and narrative synthesis were consistent (Chai et al., 2021, Jin et al., 2021).

In terms of socio-economic outcomes, findings from one review with critically low confidence on the AMSTAR 2 instrument suggest an association between quarantine and long absence from work among healthcare workers (table 15) (Yaghoubi et al., 2021).

<u>Table 12: Characteristics of reviews</u>

**Review *(rapid status in brackets)***	**Type of intervention**	**Population; Setting**	**# of studies included *(#RCTs in bracket)***	**AMSTAR rating**	**Date last searched**	**Meta-analysis conducted**
(Stratil et al., 2021) (Rapid Review)	Quarantine	Adults; Long-term care facilities	2	High Confidence	January 2021	No
(Girum et al., 2020)	Quarantine (national-based quarantine strategies, metropolitan-based quarantine strategies, and laboratory-	Population at risk and affected by COVID-19; (individuals who had contact with confirmed or suspected cases of COVID-19, who travelled from countries	5	Critically low	June 02, 2020	No

	based quarantine)	with a declared outbreak, or who live in regions with high disease transmission); Community setting				
(Cavicchioli et al., 2021)	Quarantine	General population; Not specified	21	Critically low	July 2020	No
(Chai et al., 2021)	Quarantine	Children/Adolescent; Not specified	12	Low	March 2021	Yes
(Rajkumar et al., 2022)	Quarantine	General population; Not specified	74	Critically low	August 2020	No
(Schmidt et al., 2021)	Quarantine	General population; Not specified	4	Critically low	March 2021	No
(Jin et al., 2021)	Quarantine	Children, adults, mixed groups, college students; Healthcare and educational setting	28	Critically low	October 2020	Yes
(Burns et al., 2021) (Rapid Review)	Quarantine	General population; Point of entry	12	High	November 2020	No
(Yaghoubi et al., 2021)	Quarantine	Healthcare workers; Healthcare facility	39	Critically low	August 2020	No
(Qathrin et al., 2021)	Quarantine	COVID-19 sufferers in quarantine centers/hospitals or suspected cases; Healthcare facility	7	Critically low	December 2020	No

CONFIDENTIAL

(Bonati et al., 2022)	Quarantine	General population; Community setting	105	Critically low	April 2021	No

Table 13: COVID-19 epidemiological outcomes

Review	Risk and incidence	Transmission related outcomes	Mortality
(Stratil et al., 2021)<br>(Rapid Review)<br>GRADE: Yes; low to very low certainty evidence	Isolating infected individuals from non-infected residents or staff may reduce infection rates. This separation might also reduce outbreaks and deaths, although evidence for the latter is uncertain.<br>Self-confinement of staff with residents may reduce the number of infections<br>Quarantine for new admissions may reduce the number of infections<br><br>Separating infected and non-infected residents or staff caring for them may reduce the number of infections<br><br>It is unclear whether isolation of cases reduces the number of infections	It is unclear whether isolation of cases reduces the probability of outbreaks<br><br>Separating infected and non-infected residents or staff caring for them may reduce the probability of outbreaks	Self-confinement of staff with residents may reduce the number of deaths.<br><br>It is uncertain whether Separating infected and non-infected residents or staff caring for them reduces the number of deaths
(Burns et al., 2021)<br>(Rapid Review)<br>GRADE: Yes, low to very low certainty evidence	It is uncertain whether quarantine reduces the number of cases in the community. Three modelling studies predicted that the reduction in the number of cases in the community may range from 450 to over 64,000 fewer cases, with such variations potentially related to the duration of quarantine and compliance.	Quarantine may delay time to outbreaks but it is uncertain whether it reduces the proportion of imported cases	
(Girum et al., 2020)<br><br>GRADE : Yes, but results not reported	Quarantine implemented as self-quarantine and group quarantine may reduce the incidence of infection<br><br>Implementation of early quarantine measures may make the strategy a more cost effective one. However, integration of quarantine with other public health measures may increase the effectiveness and efficiency of the program	Quarantine implemented as self-quarantine and group quarantine may reduce shorten the duration of the epidemic	Quarantine of people exposed to a confirmed case may avert deaths

Table 14: Unintended health and socio-economic outcomes

CONFIDENTIAL

Review	Non-COVID-19 related health outcomes		Socio-economic outcomes
	**Mental health**	**Substance use**	**Absenteeism**
(Cavicchioli et al., 2021)<br>GRADE: No	People exposed to quarantine for the management of pandemic infections reported clinically significant levels of psychological distress, especially PTSD (21%) and depressive (22.69%) symptoms		
(Chai et al., 2021)<br>GRADE: No	The pooled prevalence of mental problems was 28% (95% confidence interval, CI: 0.22–0.34), and the depression and anxiety problem for children and adolescents in China was 22% (95% CI: 0.16–0.30) and 25% (95% CI: 0.20–0.32) based on a random effect model, separately.		
(Rajkumar et al., 2022)<br>GRADE: No	Individuals subjected to quarantine experienced feelings of anxiety, depression, post-traumatic stress symptoms, sleep disturbances, and somatic difficulties. Several primary factors that increase the risk during quarantine include being young, identifying as female, having limited financial resources, fear of infection, poor sleep quality, reduced physical activity, increased sedentary behaviors, and a lack of social support. Protective factors included coping skills, home based exercise, leisure, recreational activities, maintaining relationships using social media and availability of mental health services.		
(Schmidt et al., 2021)<br>GRADE: No		Mixed effect: two studies found non-significant impacts of quarantine on alcohol use while one found it was associated with increased alcohol use and one found those who were self-isolating alone had lower alcohol use than those not in isolation	
(Jin et al., 2021)<br>GRADE: No	Quarantine exhibited a positive association with anxiety, depression, and stress. The correlation coefficients between		

CONFIDENTIAL

	quarantine and mental health ranged from 0.1 to 0.2, signifying a moderate positive correlation.		
(Yaghoubi et al., 2021) GRADE: No	A wide range of psychological disorders observed among healthcare workers: 5.2 to 71.2% in anxiety, 1.00 to 88.3% in stress, 8.27 to 61.67% in insomnia, and 4.5 to 50.4% in depression.		Long absence from work due to quarantine
(Qathrin et al., 2021) GRADE: No	Centralized quarantine has negative consequences for the mental health of sufferers of COVID-19. Depression and anxiety were reported to be health problems experienced by participants in all studies. Variables linked to mental health disorders include female gender, lower educational attainment, lower income, and marital status. Social stressors such as community stigma, insufficient information, and substandard quarantine facilities contribute to the deterioration of mental health conditions.		
(Bonati et al., 2022) GRADE: No	Anxiety, depression, distress and post-traumatic symptoms are frequently experienced during the COVID-19 quarantine and are often associated with changes in sleeping and eating habits.		

ii. Combined surveillance and/or response measures compared with no surveillance and/or response measures (n=6 reviews)

We included six reviews on the use of combined surveillance and response measures, of which three were systematic reviews and three rapid reviews; none with a search date after 2022 (table 15) (Ayouni et al., 2021, Girum et al., 2020, Nussbaumer-Streit et al., 2020, Talic et al., 2021). Two of the reviews were rated as high confidence on the AMSTAR 2 instrument.

All reviews examined epidemiological-related outcomes (table 16). The reviews with high confidence on the AMSTAR 2 instrument found the following: Combining quarantine and screening at borders may increase proportion of cases detected and reduce days at risk of transmitting the infection into the community, but it is uncertain whether it delays time to outbreak (Burns et al., 2021). It is uncertain whether mass testing and isolation, or symptomatic screening and isolations in school settings reduce transmission-related outcomes (reduction in the number or proportion of cases, reproduction number, number or proportion of deaths, shift in pandemic development), hospitalizations, and the number of days spent in schools (Krishnaratne et al., 2022b). The findings of the four reviews with very low confidence on the AMSTAR 2 instrument suggest that quarantine and isolation may reduce

CONFIDENTIAL

the incidence, transmission rates, case growth and mortality related to COVID-19 (Ayouni et al., 2021, Girum et al., 2020, Nussbaumer-Streit et al., 2020, Talic et al., 2021). Among the four reviews, two applied GRADE (Nussbaumer-Streit et al., 2020, Girum et al., 2020).

Table 15: Characteristics of reviews

**Review *(rapid status in brackets)***	**Type of Intervention**	**Population; Setting**	**# of studies included *(#RCTs in bracket)***	**AMSTAR rating**	**Date last searched**	**Meta-analysis conducted**
(Burns et al., 2021) (Rapid review)	Symptom/exposure-based screening or test-based screening (commonly specifying polymerase chain reaction (PCR) testing), or both	General population; point of entry	11	High	November, 2020	No
(Krishnaratne et al., 2022b) (Rapid Review)	Testing and isolation, symptomatic screening and isolation; quarantine,	More than one (children/ adolescent, adults); Educational setting	14	High	December, 2020	No
(Talic et al., 2021)	Quarantine or isolation	General population; Not specified	2	Critically low	November 2021	Yes
(Ayouni et al., 2021)	Isolation, quarantine of close contacts and home quarantine	Not specified; Not specified	6	Critically low	March 2021	No
(Nussbaumer-Streit et al., 2020) (Rapid review)	Quarantine, Isolation	Individuals who had contact with confirmed or suspected cases of COVID-19, who travelled from countries with a declared outbreak, or who live in regions with high disease transmission; Educational, Healthcare, Occupational,	15	Critically low	June 2020	No

CONFIDENTIAL

		household, and point of entry				
(Girum et al., 2020)	Quarantine, Isolation	Population at risk and affected by COVID-19 (individuals who had contact with confirmed or suspected cases of COVID-19, who travelled from countries with a declared outbreak, or who live in regions with high disease transmission); Healthcare and community settings	22	Critically low	June 2020	No

Table 16: COVID-19 epidemiological outcomes

Review	Risk and incidence	Transmission-related outcomes	Mortality
(Burns, Movsisyan et al. 2021) (Rapid review) GRADE: Yes, low certainty evidence		Combining quarantine and screening at borders may increase proportion of cases detected and reduce days at risk of transmitting the infection into the community, but it is uncertain whether it delays time to outbreak	
(Krishnaratne, Littlecott et al. 2022) (Rapid Review) GRADE: Yes, very low certainty evidence		It is uncertain whether mass testing and isolation, or symptomatic screening and isolations in school settings reduce transmission-related outcomes (reduction in the number or proportion of cases, reproduction number, shift in pandemic development), and the number of days spent in schools.	It is uncertain whether there is a reduction in the number or proportion of deaths or hospitalizations,

CONFIDENTIAL

(Talic et al., 2021) GRADE: No		A reduction in transmission of SARS-CoV-2 as a result of quarantine or isolation.	
(Ayouni et al., 2021) GRADE: No		A suppression in transmission of COVID-19 as a result of isolation, quarantine of close contacts and home quarantine.	
(Nussbaumer-Streit et al., 2020) (Rapid review) GRADE: Low certainty evidence	A reduction of incident cases as result of quarantine and isolation	A reduction in the basic reproduction number ranging from 37% to 88% as result of quarantine and isolation	A reduction of deaths as result of quarantine and isolation
(Girum et al., 2020) GRADE: Yes, but not reported	An increased effectiveness of quarantine in preventing COVID-19 infection when implemented with isolation		

c. Services (n=11 reviews)

Eleven reviews (seven systematic reviews and four rapid reviews) examined the association between PHSM targeting services such as businesses and schools on COVID-19 transmission-related outcomes (n=6) as well as non-COVID-19 related outcomes (n=6).

Below, we present the findings by comparisons.

i. School closure compared with no closure (n=6 reviews)

We included six reviews on school closure (compared to no closure); none with a search date after 2021, and five with critically low and one low confidence on the AMSTAR instrument (table 18). Among the six reviews, one applied GRADE and was rated as low certainty evidence (Chaabane et al., 2021).

COVID-19 epidemiological outcomes were examined by two reviews with very low confidence on the AMSTAR 2 instrument (table 19) (Talic et al., 2021, Walsh et al., 2021b). Findings suggest that school closure may reduce transmission rates, case growth rate and reproduction number, and mortality related to COVID-19, while the findings were mixed for incidence of COVID-19.

Non-COVID-19 related health outcomes were examined by three reviews with very low confidence on the AMSTAR 2 instrument (table 20) Findings suggest that school closure may be negatively associated with mental health status, physical activity and nutrition among children and adolescent (Chaabane et al., 2021, Freiberg et al., 2021a, Samji et al., 2021). Findings from one of the reviews also suggest that school closure may be associated with a

CONFIDENTIAL

significant decline in the number of hospital admissions and pediatric emergency department visits (Chaabane et al., 2021).

Socio-economic outcomes were examined by two reviews with very low confidence on the AMSTAR 2 instrument (table 20). Findings suggest that school closure may be negatively associated with students' academic achievement (mathematics, reading and science) (Hammerstein et al., 2021) as well as loss of access to school-based healthcare services, special services for children with disabilities, and nutrition programs (Chaabane et al., 2021).

Table 18: Characteristics of reviews

**Review *(rapid status in brackets)***	**Type of intervention**	**Population; Setting**	**# of studies included *(#RCTs in bracket)***	**AMSTAR rating**	**Date last searched**	**Meta-analysis conducted**
(Walsh et al., 2021b)	School closures and re-openings	Children/Adolescents; Educational settings	40	Low	January, 2021	No
(Hammerstein et al., 2021)	School closures	Children/Adolescent; Educational settings	11	Critically low	April, 2021	No
(Samji et al., 2021)	School closures	Children/Adolescent; Healthcare, Educational, home, and quarantine setting	63	Critically low	February, 2021	No
(Chaabane et al., 2021) (Rapid systematic review)	School Closure	Children/Adolescent; Educational setting	10	Critically low	September, 2020	No
(Talic et al., 2021)	School closures	Population at risk and affected by COVID-19; Not specified	5	Critically low	November, 2021	Yes
(Freiberg et al., 2021b) (Rapid Review)	School closures	Students and pupils; Educational institutions	3	Critically low	March, 2021	No

Table 19: COVID-19 epidemiological outcomes

CONFIDENTIAL

Review	Risk and incidence	Transmission-related outcomes	Mortality
(Walsh et al., 2021b)<br>GRADE: No		A reduction in community transmission by up to 60%.<br><br>No increase in transmission when schools reopen	Longer time periods of 26–28 days generally expected for mortality
(Talic et al., 2021)<br>GRADE: No	May reduce the incidence of COVID-19 pandemic	A reduction in COVID-19 transmission (i.e., reproduction number)	

Table 20: Unintended health and socio-economic outcomes

Review	Non-COVID-19 related health outcomes			Socio-economic outcomes
	**Mental health**	**Physical activity and /or nutrition**	**Health service utilization**	**Educational attainment**
(Hammerstein et al., 2021)<br>GRADE: No				Negative association between school closures and students' achievements (mathematics, reading and science), specifically in younger students and students from families with low socioeconomic status.
(Samji et al., 2021)<br>GRADE: No	A high prevalence of COVID-19-related fear was noted among children and adolescents, as well as more depressive and anxious symptoms compared with pre-pandemic estimates. Older adolescents, girls, and children and adolescents living with neuro-diversities and/or chronic physical conditions were			

CONFIDENTIAL

	more likely to experience negative mental health outcomes. Many of the included primary studies reported mental health deterioration among children and adolescents due to COVID-19 pandemic control measures.<br><br>Increased estimates of suicidal ideation in comparison to pre-pandemic estimates.			
(Chaabane et al., 2021) (Rapid review) GRADE: Yes; low certainty of evidence	School closure contributed to increased anxiety and loneliness in young people as well as child stress, sadness, frustration, indiscipline, and hyperactivity.	The longer the duration of school closure and reduction of daily physical activity, the higher was the predicted increase of Body Mass Index and childhood obesity prevalence.	COVID-19-related school closure associated with a significant decline in the number of hospital admissions and pediatric emergency department visits.	Children's loss of access to school-based healthcare services, special services for children with disabilities, and nutrition programs.
(Freiberg et al., 2021b) (Rapid Review) GRADE: No		School closure was associated with reduced or worsened children and adolescents' habitual activities (i.e., playing outside, walking and cycling, gardening, housework).		

ii. Combined school and/or business closures, and public event ban compared with no closure or ban (n=4 reviews)

We included four reviews on school and business closures and public event ban (compared to no closure and no ban) of which three were systematic reviews and one rapid review; none with a search date after 2022 (table 21) (Freiberg et al., 2021b, Iezadi et al., 2021, Mendez-

CONFIDENTIAL

Brito et al., 2021, Talic et al., 2021). The four reviews were rated as critically low confidence on the AMSTAR instrument. None of the reviews applied GRADE.

In terms of COVID-19 epidemiological outcomes, the findings of three reviews suggest that school and business closures and public event ban may reduce the risk, incidence, transmission rates, case growth rate and reproduction number and mortality related to COVID-19 (table 22) (Iezadi et al., 2021; Mendez-Brito et al., 2021; Talic et al., 2021).

In terms of non-COVID-19 related health outcomes, one review suggested a negative association between closure of common facilities and physical activity (table 23) (Freiberg et al., 2021).

<u>Table 21: Characteristics of reviews</u>

**Review *(rapid status in brackets)***	**Type of intervention**	**Population; Setting**	**# of studies included *(#RCT in bracket)***	**AMSTAR rating**	**Date last searched**	**Meta-analysis conducted**
(Mendez-Brito et al., 2021)	School, workplace, business and venue closing and public event bans	General population; Healthcare, educational, occupational, Point of entry, public, and community setting	27	Critically low	March, 2021	No
(Talic et al., 2021)	Business closures	Population at risk and affected by COVID-19; Not specified	2	Critically low	June, 2021	Yes
(Freiberg et al., 2021b)(Rapid Review)	Cultural and sports facilities and other non-essential businesses closures	General population; Educational, Healthcare, Occupational, and Entertainment setting	Not clearly stated	Critically low	March, 2021	No
(Iezadi et al., 2021)	Large events and school closures	General population; Community setting	2	Critically low	February, 2021	Yes

<u>Table 22: COVID-19 epidemiological outcomes</u>

**Review**	**Risk and incidence**	**Transmission-related outcome**	**Mortality**

CONFIDENTIAL

(Mendez-Brito et al., 2021) GRADE: No	A reduction in COVID-19 cases (school closing was the most effective measure on the risk and incidence, followed by workplace closing, business and venue closing and public event bans)	A reduction of reproduction number between 39% and 73% associated with school closure.	May reduce mortality-related outcomes. School closing seemed to be the most effective PHSM.
(Talic et al., 2021) GRADE: No		A reduction in transmission of SARS-CoV-2 as a result of business closures.	
(Iezadi et al., 2021) GRADE: No		May reduce the case growth rate when large events and school closure were examined individually.	

Table 23: Unintended health and socio-economic outcomes

Review	Non-COVID-19 related outcomes
	Physical activity
(Freiberg et al., 2021b) (Rapid Review) GRADE: No	A decrease in the physical activity and walking distance rate due to the closure of common facilities

iii. Measures reducing the opportunities for contacts compared with no measures (n=3 reviews)

We included three reviews on measures reducing the opportunities for contacts (compared to no measures); of which one was a systematic review and two rapid reviews; none with a search date after 2022 (table 24) (Hugelius et al., 2021, Stratil et al., 2021, Krishnaratne et al., 2022b). Two of the reviews were rated as high confidence and one low confidence on the AMSTAR instrument. Two of the reviews applied GRADE, with low to very low certainty evidence.

In terms of COVID-19 epidemiological outcomes, the two reviews with high confidence on the AMSTAR 2 instrument found the following (table 25): Within long-term care facilities, admission restrictions for visitors and new residents may reduce the number of infections. Admission restrictions for visitors may also reduce the number of deaths. It is uncertain whether cohorting residents and staff or contact reducing measures increase or decrease the number of infection or probability of outbreaks (Stratil et al., 2021). Within school settings, it is uncertain whether alternating attendance and reduced class size reduce transmission-related outcomes (i.e. a reduction in the number or proportion of cases, risk of infection, reproduction number, number or risk of deaths), and healthcare utilization (i.e. fewer hospitalizations and reduced number or proportion of cases requiring intensive care) (Krishnaratne et al., 2022b). Findings from the review with low confidence on the AMSTAR 2 instrument suggest that visiting restrictions in healthcare facilities may reduce the

CONFIDENTIAL

transmission of COVID-19 when family members followed the visiting restrictions (Hugelius et al., 2021).

In terms of socio-economic outcomes, this was examined by three reviews (table 26); findings from the two reviews with high confidence on the AMSTAR 2 instrument suggest it is uncertain whether alternating attendance and reduced class size affect the number of days spent in school (Krishnaratne et al., 2022b) and whether admission restrictions for visitors in long-term care facilities adversely affect the mental health of residents (Stratil et al., 2021). Findings from the review with low confidence on the AMSTAR 2 instrument suggest a negative association between visiting restrictions in healthcare facilities and physical activity, mental health, family relations and provision of care for both patients and healthcare providers (Hugelius et al., 2021).

Table 24: Characteristics of reviews

**Review *(rapid status in brackets)***	**Type of interventio n**	**Population; Setting**	**# of studies included *(#RCT in bracket)***	**AMSTAR rating**	**Date last searched**	**Meta-analysis conducted**
(Krishnaratne et al., 2022b) (Rapid Review)	Alternating attendance, reduced class size	More than one (children/ adolescent, adults); Educational setting	23	High confidence	December, 2020	No
(Stratil et al., 2021) (Rapid Review)	Separation of infected and non-infected residents or staff caring for infected and non-infected residents	Adults; Long-term care facilities	6	High confidence	January, 2021	No
(Hugelius et al., 2021)	Visiting restrictions	Not specified; Long-term care facilities, healthcare facilities	17	Critically low	November 2020	No

Table 25: COVID-19 epidemiological outcomes

**Review**	**Risk and incidence**	**Transmission-related outcomes**	**Mortality**	**Hospitalizations**

(Krishnaratne et al., 2022b) (Rapid Review) GRADE: Very low certainty evidence	May reduce the risk of infection and number or proportion of cases but the evidence is very uncertain	May slow the pandemic development or may reduce the length of an outbreak<br><br>May reduce the reproduction number, but the evidence is very uncertain	May reduce the number or proportion of deaths, but the evidence is very uncertain	May reduce healthcare utilization (i.e. fewer hospitalizations), but the evidence is very uncertain<br><br>May reduce the number or proportion of cases requiring intensive care, but the certainty of evidence is very low
(Stratil et al., 2021) (Rapid Review) GRADE: Yes; very low to low certainty evidence	May reduce the number of infections as a result of cohorting residents and staff, but the evidence is very uncertain<br><br>May reduce the number of infections as a result of separating infected and non-infected residents or staff caring for them, but the evidence is very	May reduce the probability of outbreaks as a result of separating infected and non-infected residents or staff caring for them (low certainty)	May reduce the number of deaths as a result of self-confinement of staff with residents, but the evidence is very uncertain.<br><br>May reduce the number of deaths as a result of separating infected and non-infected residents or staff caring for them, but the evidence is very uncertain	
(Hugelius, Harada et al. 2021) GRADE: No		A reduction in the transmission of COVID-19 when family members accepted and followed the visiting restrictions in healthcare settings.		

Table 26: Unintended health and socio-economic outcomes

Review	Socio-economic outcomes		Non-COVID-19 related health outcomes	
	**Number of days spent in school**	**Social cohesion**	**Mental Health**	**Physical Activity**
(Stratil et al., 2021) GRADE: Yes; very low certainty evidence	The evidence is very uncertain about admission restrictions for visitors increasing or decreasing the adverse mental			

CONFIDENTIAL

	health effects among residents.			
(Krishnaratne et al., 2022b) (Rapid Review) GRADE: Very low certainty evidence	Modelling studies suggest a reduction in the number of days spent in school due to the intervention, but in some cases, the initial reduction in days spent in school may be offset by an increase in the number of intended days spent in school due to their ability to prevent days lost due to quarantine or isolation.			
(Hugelius, Harada et al. 2021)<br><br>GRADE: No		A reduction in bonding of family members of neonatal intensive care unit patients with their child and family relation disturbances due to the restrictions. A reduction in the number of visits of family members such as siblings or elderly family members to neonatal intensive care units compared to before the pandemic.	A reduction in cognitive ability and overall dissatisfaction was observed in patients suffering from loneliness, depressive symptoms, agitation, aggression.<br><br>An increased need for information from care providers was reported for family members experiencing worry, anxiety and uncertainty.	A reduction in nutrition intake, decreased activities of daily living and increased physical pain and symptoms were reported.

d. Social interactions (n=9 reviews)

Nine systematic reviews examined the associations between social interaction measures and COVID-19 epidemiological outcomes (n=8) as well as non-COVID-19 related health outcomes (n=1).

CONFIDENTIAL

Below, we present the findings by comparisons.

i. Physical distancing compared with no physical distancing (n=4 reviews)

We included four reviews on the use of physical distancing (compared to no physical distancing); none with a search date after 2021, and with all rated as critically low confidence on the AMSTAR instrument (table 27). None of the reviews applied GRADE.

In terms of COVID-19 epidemiological outcomes, findings from three reviews suggest physical distancing may reduce risk and incidence, as well as transmission-related outcomes. Findings from the meta-analysis and narrative synthesis were consistent (table 28) (Ayouni et al., 2021, Khosravizadeh et al., 2021, Krishnaratne et al., 2022b).

In terms of non-COVID-19 related health outcomes, the findings from one critically low review suggest an increase in mental health among children and adolescents (table 29) (Samji et al., 2021).

Table 27: Characteristics of reviews

**Review** *(rapid status in brackets)*	**Type of Intervention**	**Population; Setting**	**# of studies included** *(#RCT in bracket)*	**AMSTAR rating**	**Date last searched**	**Meta-analysis conducted**
(Talic et al., 2021)	Contact-restriction procedures such as physical distancing	General population; Not specified	8	Critically low	Not specified; 2021	Yes
(Khosravizadeh et al., 2021)	Physical distancing strategies	Adults; Not specified	13	Critically low	March, 2021	No
(Samji et al., 2021)	Physical distancing	Children/Adolescent; Not specified	63	Critically low	February 22, 2021	No
(Ayouni et al., 2021)	Physical distancing	Not specified; Not specified	8	Critically low	Not specified; 2021	No

Table 28: COVID-19 epidemiological outcomes

**Review**	**Risk and incidence**	**Transmission-related outcomes**
(Talic et al., 2021) GRADE: No	A 25% reduction in incidence of covid-19 (relative risk 0.75, 95% confidence interval 0.59 to 0.95, I2=87%) as a result of physical distancing.	

CONFIDENTIAL

(Khosravizadeh et al., 2021) GRADE: No	May reduce the prevalence of COVID-19 pandemic	
(Ayouni et al., 2021) GRADE: No		May reduce the transmission of COVID-19

Table 29: Unintended health and socio-economic outcomes

**Review**	**Non-COVID-19 related outcomes**
	**Mental health**
(Samji et al., 2021) GRADE: No	Many studies reported the occurrence of negative emotions or mental health deterioration due to COVID-19 pandemic physical distancing measures. Physical distancing may result in increased loneliness.

ii. *Stay-at-home orders compared with no stay-at-home orders (n=2 reviews)*

We included two systematic reviews on stay-at-home orders (compared with no stay-at-home orders); with a search date of 2021 and rated as low to critically low confidence on the AMSTAR instrument (table 30) (Iezadi et al., 2021, Tully et al., 2021).

In terms of COVID-19 epidemiological outcome, this was examined by one review which suggested that stay at home order may decrease daily death growth rate and daily case growth rate in the communities (table 31). The results of the heterogeneity analysis showed that there is a high heterogeneity among studies (Iezadi et al., 2021).

In terms of non-COVID-19 related health outcomes, this was examined by one review which found that stay at home orders may reduce mobility through increased time at home and reductions in visits to shops, workplaces and use of public transport, thus, fostering transmission prevention behaviors (table 32) (Tully et al., 2021).

Table 30: Characteristics of reviews

**Review *(rapid status in brackets)***	**Type of Intervention**	**Population; Setting**	**# of studies included *(#RCT in bracket)***	**AMSTAR rating**	**Date last searched**	**Meta-analysis conducted**
(Tully et al., 2021)	Stay-at-home orders	General population Not specified	9	Critically low	February 01, 2021	No
Iezadi et al., 2021	Stay at home orders	General population; Community setting	6	Low	February 01, 2021	Yes

CONFIDENTIAL

Table 31: COVID-19 epidemiological outcomes

Review	**Transmission-related outcomes**	**Mortality**
Iezadi et al., 2021<br>GRADE: No	Daily case growth rate: -4.42% CI: -6.85 to -2; I^2%: 99.8	Daily mortality growth rate:<br>-1.42%; CI: -2.46 to -0.37; I^2%: 0.00

Table 32: Unintended health and socio-economic outcomes

**Review**	**Non-COVID-19 related health outcomes**
	**Mobility**
(Tully et al., 2021)<br>GRADE: No	A reduction in mobility as a result of stay-at-home orders through increased time spent at home and reductions in visits to shops, workplaces and use of public transport.

iii. Combined social interaction measures (n=4 reviews)

We included four reviews on the use of combined social interaction measures; none with a search date after 2021 and with the majority rated as critically low confidence on the AMSTAR instrument (table 33). Among the four reviews, only one applied GRADE (Girum et al., 2021).

In terms of COVID-19 epidemiological outcomes, the findings of three reviews suggest that combined social interaction measures may reduce transmission-related outcomes (table 34) (Girum et al., 2021; Iezadi et al., 2021; Mendez-Brito et al., 2021). Findings from the meta-analysis and narrative synthesis were consistent.

In terms of non-COVID-19 related health outcomes, the findings from one review suggest an increase in depression, stress and anxiety (table 35) (Rodriguez-Fernandez et al., 2021).

Table 33: Characteristics of reviews

**Review *(rapid status in brackets)***	**Type of intervention**	**Population; Setting**	**# of studies included *(#RCT in bracket)***	**AMSTAR rating**	**Date last searched**	**Meta-analysis conducted**
(Mendez-Brito et al., 2021)	Social interaction measures	General population; Healthcare, educational, occupational, Point of entry, public, and community setting	34 studies included overall (not clear how many focused specifically on this intervention)	Critically low	March, 2021	No

CONFIDENTIAL

(Girum et al., 2021)	Social distancing measures	Individuals who have contact a confirmed or suspected case of COVID-19, or individuals who live in areas with COVID-19 outbreak; or individuals considered to be high risk for COVID-19/suspected cases, or confirmed/probable cases of COVID-19 infection; Community setting	14	Critically low	Not specified; 2020	No
(Rodriguez-Fernandez et al., 2021)	Social distancing measures	General population; Not specified	26	Critically low	January 07, 2021	No
(Iezadi et al., 2021)	State-wide social distancing policies	General population; Community setting	8	Low	February 01, 2021	Yes

Table 34: COVID-19 epidemiological outcomes

**Review**	**Transmission-related outcomes**
(Girum et al., 2021)<br>GRADE: Yes; (12 no concerns to minor concern; 2 major concerns and moderate concerns)	A reduction in the daily growth rate by 9.1%, contacts by 7–9 folds, median number of infections by 92% and epidemic resolved in day 90 as a result of mandatory social distancing.
(Iezadi et al., 2021)<br>GRADE: No	May reduce case growth rate as a result of social distancing policies<br><br>Reproduction number: -2.36, CI: -2.81 to -1.91, I^2%: 100
(Mendez-Brito et al., 2021)<br>GRADE: No	A reduction in the transmission of and control of COVID as a result of social interactions measures.

Table 35: Unintended health and socio-economic outcomes

CONFIDENTIAL

Review	Non-COVID-19 related health outcomes
	**Mental health**
(Rodriguez-Fernandez et al., 2021)<br>GRADE: No	An increase in depression, stress and anxiety as a result of social distancing. Factors contributing to this increase include being females, young age, unemployed and people with previous mental health or psychiatric illnesses the most vulnerable.

e. Movement (n=5 reviews)

Five review (four systematic reviews and one rapid review) examined the association between movement intervention and COVID-19 epidemiological outcomes (Burns et al., 2021, Girum et al., 2021, Grépin et al., 2021, Mendez-Brito et al., 2021, Talic et al., 2021).

Below, we present the findings by comparisons.

i. Travel restrictions compared with no travel restrictions (n=5 reviews)

We included five reviews on travel restrictions (compared to no restrictions); none with a search date after 2021, and four with critically low and one high confidence on the AMSTAR instrument (table 36). Only two of the reviews applied GRADE (Burns et al., 2021, Girum et al., 2021).

All reviews examined COVID-19 epidemiological outcomes (table 37). The review with high confidence on the AMSTAR 2 instrument found the following: it is uncertain whether reducing or stopping cross-border travel decreases COVID-19 cases in the community or cases exported or imported, and deaths. It is also uncertain whether cross-border travel controls can slow the spread of COVID-19 (Burns et al., 2021). The review applied GRADE.

Findings from the four e reviews with critically low confidence on the AMSTAR 2 instrument provided mixed results. Whereas three reviews suggested that travel ban may reduce the risk, incidence, transmission rates and mortality related to COVID-19, (Mendez-Brito et al., 2021, Girum et al., 2021, Grépin et al., 2021)). Findings from the remaining review suggested an increase in COVID-19 incidence after border closure and a little effect on reducing COVID-19 transmission in African countries (Talic et al., 2021). Overall, the reviews reported that travel bans are particularly useful in the early stage of an outbreak before widespread distribution of the disease.

<u>Table 36: Characteristics of reviews</u>

**Review *(rapid status in brackets)***	**Type of Intervention**	**Population; Setting**	**# of studies included *(#RCT in bracket)***	**AMSTAR rating**	**Date last searched**	**Meta-analysis conducted**
(Talic et al., 2021)	Restricted travel and border closures	Population at risk and affected by COVID-19; Not specified	2	Critically low	June 7, 2021	Yes

(Burns et al., 2021) (Rapid Review)	Travel restrictions reducing or stopping cross-border travel	General population; Point of Entry (airport)	31	High	November, 13, 2020	No
(Grépin et al., 2021)	Travel-related measures (applied either at an international or domestic border)	General population; Point of entry (incl airport, ports, land borders)	29	Critically low	June 1, 2020	No
(Girum et al., 2021)	Travel ban	Individuals who have contact a confirmed or suspected case of COVID-19, or individuals who live in areas with COVID-19 outbreak; or individuals considered to be high risk for COVID-19/suspected cases, or confirmed/probable cases of COVID-19 infection.; Community setting	9	Critically low	Not specified	No
(Mendez-Brito et al., 2021)	, Travel restrictions: None/ Partial/ Complete border closure	General population; Healthcare, educational, occupational, Point of entry, public, and community setting	17	Critically low	March 9, 2021	No

Table 37: COVID-19 epidemiological outcomes

**Review**	**Risk and incidence**	**Transmission related outcomes**	**Mortality**

CONFIDENTIAL

(Talic et al., 2021)<br>GRADE: No	An increase in the incidence of COVID-19 was reported after border closure in the African countries. A minimal effect on the incidence of COVID-19 was concluded as a result of the implementation of border closures within African countries had	A minimal effect in reducing COVID-19 transmission was concluded as a result of the border closure in Africa, but the review was assessed as being at high risk of bias.<br><br>A reduction of 11% in SARS-CoV-2 transmission in the US, as a result of the restrictions on travel between states (one included primary study).	
(Burns et al., 2021)<br>(Rapid Review)<br>GRADE: Yes; Very low certainty evidence	It is uncertain whether travel restrictions reduce COVID-19 cases in the community,	It is uncertain whether cross-border travel controls can slow the spread of COVID-19. Most studies predicted positive effects, however, results from individual studies varied from a delay of less than one day to a delay of 85 days; very few studies predicted no effect of the measure.	It is uncertain whether travel restrictions reduce deaths
(Grépin et al., 2021)<br>GRADE: No		A reduction in the number of cases exported internationally by 70%–80%.<br><br>A reduction in transmission<br><br>A delay in outbreaks by 3-5 days due to bans<br><br>A reduction by more than half of the reproductive number within 2 weeks of the introduction of the ban	
(Girum et al., 2021)<br>GRADE: Yes; 12 low, 1 moderate, 1 high	A reduction in the number of infected individuals by 91.14%		
(Mendez-Brito et al., 2021)<br>GRADE: No	A reduction in the incidence-related outcomes was associated with both domestic and international	A reduction in the reproduction number and growth rate was associated with both	A reduction in mortality in 4 out of 7 studies (57%) was associated with

CONFIDENTIAL

	travel restrictions. Early implementation was associated with a higher effectiveness.	domestic and international travel restrictions.	international travel restrictions.

ii. International travel restrictions compared with domestic or national travel restrictions (n=1 review)

We included one review on international travel restrictions (compared to domestic or national travel restrictions); with a search date of 2021 and rated as critically low confidence on the AMSTAR instrument (table 38) (Mendez-Brito et al., 2021). The findings of this review suggest that policies pertaining to international travel restrictions may be more effective than those pertaining to domestic or national travel restrictions (table 39). This review did not apply GRADE.

Table 38: Characteristics of reviews

**Review *(rapid status in brackets)***	**Type of intervention**	**Population; Setting**	**# of studies included *(#RCT in bracket)***	**AMSTAR rating**	**Date last searched**	**Meta-analysis conducted**
(Mendez-Brito et al., 2021)	, Travel restrictions: none/ partial/ complete border closure	General population; Healthcare, educational, occupational, Point of entry, public, and community setting	34 studies included overall (not clear how many focused specifically on this intervention)	Critically low	March 9, 2021	No

Table 39: COVID-19 epidemiological outcomes

**Review**	**Risk and incidence**	**Transmission-related outcomes**
(Mendez-Brito et al., 2021)<br>GRADE: No	A greater reduction in incidence-related outcomes was associated with international travel restrictions than domestic or national travel restrictions in the studies analyzing both policies	A greater reduction in the reproduction number and growth rate was associated with international travel restrictions than domestic or national travel restrictions in the studies analyzing both policies.

f. Physical Environment (n=3 reviews)

CONFIDENTIAL

Three reviews (two systematic reviews and one rapid review) examined the association between physical environment related PHSM measures and COVID-19 epidemiological outcomes (Stratil et al., 2021, Talic et al., 2021, Krishnaratne et al., 2022a).

Below, we present the findings by comparisons.

i. ***Cleaning and/or environmental hygiene measures compared with no cleaning and/or environmental hygiene measures (n=3 reviews)***

We included three reviews on the use of cleaning and environmental hygiene measures (compared to no cleaning and/or environmental hygiene measures); none with a search date after 2021 and with two rated as high and one as critically low confidence on the AMSTAR instrument (table 40) (Krishnaratne et al., 2022b, Stratil et al., 2021, Talic et al., 2021). Two of the reviews applied GRADE (Stratil et al., 2021, Krishnaratne et al., 2022b).

All reviews examined epidemiological related outcomes (table 41). Findings from the two reviews with high confidence on the AMSTAR 2 instrument suggest the following: Within long-term care facilities, it is uncertain whether cleaning or environmental hygiene measures reduce or increase the number of infections. It is also uncertain whether higher frequency of cleaning increases or decreases the number of infections. Multicomponent cleaning and environmental hygiene measures in long-term care facilities may reduce the number of infections in home care settings but it is uncertain whether it decreases the number of deaths (Stratil et al., 2021). Within school settings, it is uncertain whether cleaning reduces the reproduction number and whether improved ventilation reduces the concentration of aerosol particles containing RNA virus in the room and inhaled dose of RNA virus for a susceptible person (Krishnaratne et al., 2022a, Stratil et al., 2021).

Findings from the review with critically low confidence on the AMSTAR 2 instrument suggest that the use of disinfectants may be associated with reduced risk of secondary transmission of SARS-CoV-2 within households (Talic et al., 2021).

<u>Table 40: Characteristics of reviews</u>

**Review *(rapid status in brackets)***	**Type of Interventio n**	**Populatio n; Setting**	**# of studies included *(#RCT in bracket)***	**AMSTAR Rating**	**Date last searched**	**Meta-analysis conducted**
(Stratil et al., 2021) (Rapid review)	Cleaning and environment al hygiene measures	Adults; Long-term care facilities	1	High confidence	2021	No
(Talic et al., 2021)	Disinfection in household	General population; Not Specified;	1	Critically low	2021	Yes

CONFIDENTIAL

(Krishnaratne et al., 2022b) (Rapid Review)	Cleaning and environmental hygiene measures; improved ventilation	More than one (children/ adolescent, adults); Educational setting	1	High confidence	December, 2020	No

<u>Table 41: COVID-19 epidemiological outcomes</u>

**Review**	**Risk and incidence**	**Transmission-related outcomes**
(Stratil et al., 2021) (Rapid review)<br><br>GRADE: Yes; very low certainty evidence	It is uncertain whether cleaning and environmental hygiene measure reduce the number of infections.<br><br>It is uncertain whether higher frequency of cleaning increases or decreases the number of infections.	
(Talic et al., 2021)<br>GRADE: No;		Association between disinfection of surfaces and reduced risk of secondary transmission of SARS-CoV-2 within households (odds ratio 0.23, 95% confidence interval 0.07 to 0.84) (based on one included study).
(Krishnaratne et al., 2022b)<br>GRADE: Yes; very low certainty evidence	It is uncertain whether cleaning reduces the reproduction number..<br><br>It is uncertain whether improving ventilation by using air purifiers equipped with high efficiency particulate air filters in a high school classroom reduces the concentration of aerosol particles containing RNA viruses in the room and the inhaled dose of RNA virus for a susceptible person.	

g. Individual protection (n=18 reviews)

Eighteen reviews (14 systematic reviews and four rapid reviews) examined the association between individual protection and COVID-19 epidemiological outcomes (n=12) as well as non-COVID-19 related health outcomes (n=5) (Asín-Izquierdo et al., 2022, Ayouni et al., 2021, Ford et al., 2021, Iezadi et al., 2021, Ingram et al., 2021, Kunstler et al., 2022, Mendez-Brito et al., 2021, Shekaraiah and Suresh, 2021, Stratil et al., 2021, Tabatabaeizadeh, 2021, Talic et al., 2021, Tully et al., 2021, Daniela et al., 2020, Byambasuren et al., 2021, Desye, 2021, Galanis et al., 2021, Khatib et al., 2022, Krishnaratne et al., 2022a).

CONFIDENTIAL

Below, we present the findings by comparisons.

i. *Face Mask compared with no face mask (n=13 reviews)*

We included 13 reviews on the use of face masks (compared to no use of face masks); none with a search date after 2021 and with one rated as high and 12 as critically low confidence on the AMSTAR instrument (table 42).

In terms of COVID-19 epidemiological outcomes, the review with high confidence on the AMSTAR 2 instrument found that it is uncertain whether face mask use in school settings reduces transmission-related outcomes (number or proportion of cases, reproduction number or the number or proportion of deaths) and healthcare utilization (number or proportion of hospitalization) (Krishnaratne et al., 2022a). In line with the above, findings from two reviews with critical low confidence review also suggest that masking alone may not provide sufficient protection from COVID-19 outbreaks in the workplace (Igram et al 2021; Kunstler et al 2022). One of the reviews found no statistically significant difference in the likelihood of healthcare workers wearing surgical masks or respirators becoming infected with COVID-19 (Kunstler et al 2022). The remaining 10 reviews with critically low confidence on the AMSTAR 2 instrument suggest that face mask use in general or in community settings (e.g. mandatory masking policies) may reduce the risk, incidence, transmission-related outcomes and mortality related to COVID-19 pandemic. Among the included reviews, two applied GRADE with the majority of outcomes falling under the low certainty category (Shekaraiah and Suresh, 2021, Krishnaratne et al., 2022a) (table 43).

In terms of non-COVID-19 related health outcomes, these were examined by four reviews with critically low confidence on the AMSTAR 2 instrument. Findings from two of the reviews suggest limited or no associated between face mask use and mobility or voice production (e.g. perceptual, acoustic, aerodynamic, physiological) (Shekaraiah and Suresh, 2021, Tully et al., 2021) while findings from the other two reviews suggest associations with physiological outcomes (Kunstler et al., 2022, Asín-Izquierdo et al., 2022). In one of the reviews, facemask use during exercise may be associated with increased heart rate, perceived exertion, and dyspnea, and reduced pulmonary function parameters (Kunstler et al., 2022). In the other review, healthcare workers wearing respirators reported more adverse events (including de novo headache, respiratory distress or shortness of breath, facial itching or irritation, and pressure-related injuries) compared to those wearing surgical masks (Asín-Izquierdo et al., 2022). Out of the four reviews, only one applied GRADE with the outcomes rated as low certainty evidence (Shekaraiah and Suresh, 2021) (table 44).

Findings from the meta-analysis and narrative synthesis were consistent.

CONFIDENTIAL

Table 42: Characteristics of reviews

**Review *(rapid status in brackets)***	**Type of intervention**	**Population; Setting**	**# of studies included *(#RCT in bracket)±***	**AMSTAR rating**	**Date last searched**	**Meta-analysis conducted**

(Krishnaratne et al., 2022b) (Rapid Review)	Mask wearing	More than one (children/ adolescent, adults); Educational setting	4	High confidence	December, 2020	No
(Ingram et al., 2021) (Rapid Review)	Universal masking policies	Not specified; Occupational setting	3	Critically low	Not Specified; 2020	Yes
(Mendez-Brito et al., 2021)	Mask wearing	General population; Healthcare, educational, occupational, point of entry, and public, community setting	7	Critically low	March, 2021	No
(Talic et al., 2021)	Mask wearing	General population; Not specified	11	Critically low	Not Specified; 2021	Yes
(Ayouni et al., 2021)	Mask wearing	Not Specified; Community setting	18 studies included overall (not clear how many focused specifically on this intervention)	Critically low	Not Specified; 2021	No
(Tully et al., 2021)	Mask	General population; Not Specified	2	Critically low	February, 2021	No
(Tabatabaeizadeh, 2021)	Face mask	General population; Not Specified	4	Critically low	October, 2020	Yes
(Kunstler et al., 2022)	P2/N95 (or equivalent) respirators and surgical masks	Healthcare workers; Healthcare Setting	21 (1 is RCT)	Critically low	June, 2021	Yes
(Asín-Izquierdo et al., 2022)	Face mask	Adults; Healthcare and occupational setting	8 (3 are RCTs)	Critically low	March, 2021	No

CONFIDENTIAL

(Shekaraiah and Suresh, 2021)	Masks (surgical, N95/KN95, and cloth masks)	General population; Not Specified	10	Critically low	April, 2021	No
(Daniela et al., 2020) (Rapid Review)	Face mask	General population; Household and community settings	7	Critically low	August, 2020	No
(Ford et al., 2021)	Mask wearing	General population; Community settings	21	Critically low	March, 2021	No
(Iezadi et al., 2021)	Mask wearing	General population; Community settings	1	Critically low	February, 2021	Yes

Table 43: COVID-19 epidemiological outcomes

**Review**	**Risk and incidence**	**transmission-related outcomes**	**Mortality**
(Ingram et al., 2021)<br>GRADE: No	Universal masking studies individually reported 11.5% (1129/9850) and 39.5% (725/1834) post-intervention positivity, accounting for 3.2% and 4.4% reductions in COVID-19 positivity, respectively.		
(Mendez-Brito et al., 2021)<br>GRADE: No	A reduction in COVID-19 cases as a result of mask-wearing requirements.		
(Talic et al., 2021)<br>GRADE: No	53% reduction in COVID-19 incidence (RR: 0.47, 95% CI: 0.19 -1.12, I2=12%).	29% reduction in COVID-19 transmission rates in states where mask wearing was mandatory.	45.7% reduction in COVID-19 related mortality where mask wearing was mandatory (RR: 0.71, 95% CI: 0.58 – 0.75).<br>.
(Ayouni et al., 2021)<br>GRADE: No		Compulsory mask wearing and community wide masking may contribute to COVID-19 control when implemented with other non-pharmaceutical control measures.	

CONFIDENTIAL

(Tabatabaeizadeh, 2021)<br>GRADE: No	A significant decreased risk of COVID-19 infection (RR: 0.12, 95% CI: 0.06-0.27 ($P < 0.001$) as a result of face mask		
(Kunstler et al., 2022)<br>GRADE: No	No statistically significant difference in the likelihood of healthcare workers wearing surgical masks or respirators becoming infected with SARS-CoV-2 (OR: 0.85, 95% CI: 0.72-1.01, I2 = 60%).,		
(Daniela et al., 2020)<br>GRADE: No		A reduction in the transmission of COVID-19 in community and household settings as a result of wearing mask according to observational studies. The reduction did not differ between mask types (cloth, surgical, N95 or other (unspecified) mask types).	
(Ford et al., 2021)<br>GRADE: No	"A rapid and significant reduction in incidence associated with mask wearing policies."		Mask wearing policies may have protective benefits in terms of reduced COVID-19 mortality.
(Iezadi et al., 2021)<br>GRADE: No		A reduction in case growth rate as a result of face mask use recommendations.	
(Krishnaratne et al., 2022b)<br><br>GRADE: Yes; very low certainty evidence	Uncertain whether face mask use in school settings reduces transmission-related outcomes (number or proportion of cases, reproduction number or the number or proportion of deaths)		

<u>Table 44: Unintended health and socio-economic outcomes</u>

**Review**	**Non-COVID-19 related outcomes**		
	**Mobility**	**Physiological outcomes**	**Voice production**
(Tully et al., 2021)<br>GRADE: No	Little to no association between face mask mandates and mobility.		

CONFIDENTIAL

(Asín-Izquierdo et al., 2022) GRADE: No		Face mask use during exercise increased heart rate, perceived exertion, and dyspnea, while reducing pulmonary function parameters. Some studies reported discomfort; no significant differences in SpO2, tissue oxygenation, BP, or blood lactate concentration.	
(Kunstler et al., 2022) GRADE: No		Healthcare workers experienced significantly more headaches (OR 2.62, [95%CI 1.18, 5.81]), respiratory distress (OR 4.21, [95%CI 1.46, 12.13]), facial irritation (OR 1.80, [95%CI 1.03, 3.14]) and pressure-related injuries (OR 4.39, [95%CI 2.37, 8.15]) when wearing respirators compared to surgical	
(Shekaraiah and Suresh, 2021) GRADE: Yes; low certainty			No significant association between wearing face mask (surgical, N95/KN95, and cloth masks) and voice measures, including self-reported measures of vocal fatigue, discomfort, and effort, as well as acoustic measures of fundamental frequency, intensity, and perturbation.

ii. Mask and PPE use compared with no mask and PPE (n=1 review)

We included one rapid review on the use of masks and PPE (compared to no mask and PPE); with a search date of 2021 and rated as high on the AMSTAR instrument (table 45). The review applied GRADE. It is uncertain whether the use of mask and PPE by staff may reduce or increase the number of infections, probability of outbreaks and number of deaths in long-term care facilities (Stratil et al., 2021) (table 46).

Table 45: Characteristics of reviews

**Review *(rapid status in***	**Type of interventi on**	**Population; Setting**	**# of studies included**	**AMSTAR rating**	**Date last searched**	**Meta-analysis conducted**

CONFIDENTIAL

brackets)			***(#RCT in bracket)***			
(Stratil et al., 2021) (Rapid Review)	Mask and PPE usage by staff	Adults; Long-term care facilities	4	High	Not Specified; 2021	No

Table 46: COVID-19 epidemiological outcomes

**Review**	**Risk and incidence**	**Transmission-related outcomes**	**Mortality**
(Stratil et al., 2021) GRADE: Very low certainty evidence	Measures may reduce the number of infections, yet evidence is subject to uncertainty.	Measure may increase the probability of outbreaks, yet evidence is subject to uncertainty.	The measure may reduce the number of deaths, but the evidence is very uncertain.

iii. Handwashing compared with no hand-washing (n=4 reviews)

We included four reviews on the use of handwashing (compared to no handwashing) of which three were systematic review and 1 rapid review; none with a search date after 2022 (table 47). One of the reviews was rated as high and three as critically low confidence on the AMSTAR instrument.

The review with high confidence on the AMSTAR 2 instrument suggest it is uncertain whether handwashing in school settings affects reproduction number (Krishnaratne et al., 2022a). Findings of the three reviews with critically low confidence on the AMSTAR 2 instrument suggest that handwashing may reduce the risk, incidence and transmission rates related to COVID-19 (Desye, 2021, Khatib et al., 2022, Talic et al., 2021). Among the four reviews, two applied GRADE and the outcomes wererated as very low certainty evidence (Khatib et al., 2022, Krishnaratne et al., 2022b).

Table 47: Characteristics of reviews

**Review *(rapid status in brackets)***	**Type of intervention**	**Population & Setting**	**Number of studies included *(#RCT in bracket)***	**AMSTAR rating**	**Date last searched**	**Meta-analysis conducted**
(Talic et al., 2021)	Handwashing	General population; Not specified	3	Critically low	Not Specified; 2021	Yes

(Khatib et al., 2022) (Rapid Review)	Handwashing	Healthcare workers; Healthcare Setting	3	Critically low	Not Specified; 2022	No
(Desye, 2021)	Hand washing with soaps	Not specified; Not specified	28 studies included overall (not clear how many focused specifically on this intervention)	Critically low	Not Specified; 2021	No
(Krishnaratne et al., 2022b)<br><br>(Rapid Review)	Handwashing	More than one (children/ adolescent, adults); Educational setting	2	High confidence	December, 2020	No

Table 48: COVID-19 epidemiological outcomes

**Review**	**Risk and incidence**	**Transmission-related outcomes**
(Krishnaratne et al., 2022a)		Uncertain whether handwashing in school settings affects reproduction number
(Talic et al., 2021)<br>GRADE: No	53% non-statistically significant reduction in COVID-19 incidence associated with handwashing (RR: 0.47, 95% CI: 0.19 - 1.12, I2=12%). A sensitivity analysis without adjustment showed a significant reduction in covid-19 incidence (0.49, 0.33 to 0.72, I^2=12%)	
(Khatib et al., 2022)<br>GRADE: Yes; very low certainty evidence	A significant reduction in the risk of developing COVID-19 by 59% when comparing optimal handwashing to suboptimal handwashing practices (based on one included study)	
(Desye, 2021)<br>GRADE: No		90% reduction in COVID-19 transmission as a result of handwashing with soap (based on one included study).

iv. PPE compared with no PPE (n=1 review)

CONFIDENTIAL

We included one systematic review on the use of PPEs (compared to no PPEs), with a search date of 2020; and rated as critically low confidence on the AMSTAR instrument (table 49). Findings suggest PPE usage may be associated with adverse events among healthcare workers (headache, dry skin, dyspnea, pressure injuries, itching, hyperhidrosis, dermatitis) (Galanis et al., 2021). The review did not apply GRADE (table 50).

Table 49: Characteristics of reviews

**Review *(rapid status in brackets)***	**Type of intervention**	**Population & Setting**	**Number of studies included *(#RCT in bracket)***	**AMSTAR rating**	**Date last searched**	**Meta-analysis conducted**
(Galanis et al., 2021)	PPE usage	Healthcare workers; Healthcare Setting	14	Critically low	December, 2020	Yes

Table 50: Unintended health and socio-economic outcomes

**Review**	**Non-COVID-19 related health outcomes**
	**Physiological outcomes**
(Galanis et al., 2021) GRADE: No	Estimated prevalence of adverse events among healthcare workers due to PEE (headache, dry skin, dyspnea, pressure injuries, itching, hyperhidrosis, dermatitis) was 78%, ranging from 42.8% to 95.1% across the included studies. The factors associated with an increased risk of adverse events among healthcare workers were obesity, diabetes, smoking, pre-existing headache, longer duration of shifts wearing PPE, increased consecutive days with PPE, and increased exposure to confirmed or suspected COVID-19 patients.

v. Universal masking compared with combined Infection Prevention and Control (IPC) (n=1 review)

We included one rapid review on the use of universal masking versus combined IPC; with a search date of 2020 and rated as critically low confidence on the AMSTAR instrument (table 51). Findings suggest lower COVID-19 positivity rates among employees with combined IPC compared to universal masking (Ingram et al., 2021). The review did not apply GRADE (table 52).

Table 51: Characteristics of reviews

CONFIDENTIAL

Review *(rapid status in brackets)*	Type of intervention	Population & Setting	Number of studies included *(#RCT in bracket)*	AMSTAR rating	Date last searched	Meta-analysis conducted
(Ingram et al., 2021) (Rapid Review)	IPC measures (contact tracing and case isolation, PPE, and facility zoning)	Not Specified; Healthcare and occupational setting	15	Critically low	Not Specified; 2020	Yes

Table 52; COVID-19 epidemiological outcomes

Review	Risk and incidence
(Ingram et al., 2021)<br>GRADE: No	Combined IPC measures (incorporating swift contact tracing and case isolation, PPE, and facility zoning) resulted in lower employee COVID-19 positivity rates (0.2% positivity; 95% CI 0–0.4%) than single measures such as universal masking (24%; 95% CI 3.4–55.5%).

vi. Eye Protection compared with no eye protection (n=1 review)

We included one systematic review on the use of eye protection (including face shields, goggles, or modified snorkel masks) with or without face mask (compared to no eye protection), with a search date of 2021 and rated as low confidence on the AMSTAR instrument (table 53). Findings suggest the use of eye protection may reduce the risk and incidence related to COVID-19 (Byambasuren et al., 2021). The review did not apply GRADE (table 54).

Table 53: Characteristics of reviews

Review *(rapid status in brackets)*	Type of intervention	Population) & Setting	Number of studies included *(#RCT in bracket)*	AMSTAR rating	Date last searched	Meta-analysis conducted

CONFIDENTIAL

(Byambasuren et al., 2021)	Any form of eye protection including face shields and variants, goggles, and glasses	General population; Community and Healthcare Setting	5	Low	June, 2021	Yes

Table 54: COVID-19 epidemiological outcomes

**Review**	**Risk and incidence**
(Byambasuren et al., 2021)<br>GRADE: No	A 96% to 40% reduction in COVID-19 incidence (OR:0.04 -0.6).

vii. Hand sanitizer compared with no hand sanitizer (n=1 review)

We included one systematic review on the use of hand sanitizer (compared to no hand sanitizer); with a search date of 2021 and rated as critically low confidence on the AMSTAR instrument (table 55). Findings of the review suggest that hand sanitizer may reduce the transmission rates related to COVID-19 (Desye, 2021). The review also suggests risk of toxicity due to absorption of disinfectant through dermal contact and accidental ingestion. The review did not apply GRADE (Desye, 2021) (table 56 and 57).

Table 55: Characteristics of reviews

**Review *(rapid status in brackets)***	**Type of Intervention**	**Population; Setting**	**# of studies included *(#RCT in bracket)***	**AMSTAR rating**	**Date last searched**	**Meta-analysis conducted**
(Desye, 2021)	Hand sanitizer with alcohol	Not Specified; Not Specified	28 studies included overall (not clear how many focused specifically on this intervention	Critically low	2021	No

CONFIDENTIAL

Table 56: COVID-19 epidemiological outcomes

Review	Transmission related outcomes
(Desye, 2021)<br>GRADE: No;	Hand sanitizer with alcohol may reduce transmission of COVID-19.

Table 57: Unintended health and socio-economic outcomes

Review	Non-COVID-19 related health outcomes
	**Physiological outcomes**
(Desye, 2021)<br>GRADE: No;	A risk of toxicity exists due to absorption through dermal contact and accidental ingestion.

h. Multicomponent interventions (n=64 reviews)

A total of 64 reviews examined the association between multi-component interventions and COVID-19 epidemiological outcomes as well as non-COVID-19 related health outcomes and socio-economic outcomes.

Below, we present the findings by comparison.

i. Combination of PHSMs compared with no PHSMs (n=35 reviews)

We included 35 reviews on the use of multicomponent PHSMs (compared with no PHSMs). Most of the reviews had search dates between 2020 and 2021 with only two reviews in 2022 (table 58). Four of the reviews were rated as high or moderate confidence while the remaining were rated as critically low confidence on the AMSTAR 2 instrument.

In terms of COVID-19 epidemiological outcomes, the reviews with high or moderate confidence on the AMSTAR 2 instrument found the following (table 59): Multi-component interventions in long-term care facilities (including entry regulation, transmission and contact control, surveillance, and outbreak control measures) may reduce the number of infections but it is unclear whether they reduce the number of deaths (Stratil 2021). Multicomponent

CONFIDENTIAL

interventions at mass gatherings (provision of hand sanitizer, wearing of face masks, ensuring adequate ventilation, health screening (i.e., temperature, symptom, travel or close contact screening) and contact tracing) may reduce the risk of COVID-19 transmission; however, it is unlikely that this risk can be eliminated entirely (Walsh et al., 2021a). Evidence on multicomponent PHSMs in school settings was mixed; while most of the studies suggested PHSMs may decrease the number or proportion of cases, some showed mixed or no effects (1 high confidence review; GRADE applied) (Krishnaratne et al., 2022a). A combination of quarantine and screening measure at borders may increase proportion of cases detected and reduce days at risk of transmitting the infection into the community, but it is uncertain whether it delay time to outbreak (Burns et al., 2021). Only one review reported findings from one study indicating that there was no association between multi-component PHSMs and COVID-19 transmission during swimming activities (Yaacoub et al., 2021).

Findings from the 13 reviews with critically low confidence on the AMSTAR 2 instrument suggest that multi-component interventions may reduce the risk, incidence, transmission rates, case growth rate, reproduction number, and mortality related to COVID-19 (Ingram et al., 2021, Talic et al., 2021, Ayouni et al., 2021, Hatami et al., 2022, Nussbaumer-Streit et al., 2020, Johanna et al., 2020, Khatib et al., 2022, Girum et al., 2020, Bou-Karroum et al., 2021, Iezadi et al., 2021, Regmi et al., 2021, Mbwogge, 2021, Girum et al., 2021). Furthermore, PHSMs were associated with a reduction in COVID-19 ICU admissions (Iezadi et al., 2021). Most of the included reviews reported better outcomes when several measures are combined together compared to single interventions alone. Ten of the reviews used GRADE (Bakaloudi et al., 2021b, Bou-Karroum et al., 2021, Chaabane et al., 2021, Girum et al., 2020, Girum et al., 2021, Khatib et al., 2022, Mbwogge, 2021, Nussbaumer-Streit et al., 2020, Runacres et al., 2021, Sohi et al., 2022, Krishnaratne et al., 2022a) (table 59).

Non-COVID-19 related health outcomes were examined by 17 reviews with critically low confidence on the AMSTAR 2 instrument, of which only three applied GRADE (table 60). In terms of nutrition, three reviews reported changes in food intake and eating behaviors, and an increase in unhealthy food consumption (Neira et al., 2021, Saulle et al., 2021, Viner et al., 2022). Similarly, four reviews reported associations with decreased physical activity and mobility, and increased sedentary time (Runacres et al., 2021, Viner et al., 2022, Saulle et al., 2021, Tully et al., 2021) including increased screen time especially among children and adolescents (Saulle et al., 2021, Viner et al., 2022). With regards to mental health outcomes, a negative association was reported in multiple reviews, including increased prevalence of anxiety, depression, stress, suicide and suicidal ideation, as well as other emotional and psychological concerns (Farooq et al., 2021, Castaldelli-Maia et al., 2021, Saulle et al., 2021, Viner et al., 2022, Rajmil et al., 2021, Samji et al., 2021, Chaabane et al., 2021). An increase in sleep problems was also observed among children and adolescents (Saulle et al., 2021, Viner et al., 2022). In addition, there was association with increased substance use and particularly alcohol use (Sohi et al., 2022, Schmidt et al., 2021).

The included reviews also found association between multicomponent PHSM and health service utilization and health outcomes. There was negative association with service utilization including a decrease in emergency department visits, hospital admissions, vaccination, and access to psychiatric services (Chaabane et al., 2021, Hawco et al., 2022, Saulle et al., 2021). Some reviews also reported association with increase in violence as well as a decrease in child abuse referrals and notifications during COVID-19 (Saulle et al., 2021,

CONFIDENTIAL

Viner et al., 2022, Rajmil et al., 2021, Lausi et al., 2021, Alex et al., 2021). In contrast, one review reported associations between PHSMs and reduction in influenza mortality and morbidity (Fricke et al., 2020) while another reported reduction in preterm birth during COVID-19 measures (Hawco et al., 2022).

Table 58: Characteristics of reviews

**Review** ***(rapid status in brackets)***	**Multi-component interventions**	**Population; Setting**	**# of studies included** ***(#RCT in bracket)*** **±**	**AMSTAR rating**	**Date last searched**	**Meta-analysis conducted**
(Krishnaratne et al., 2022b)<br><br>(Rapid Review)	Multicomponent PHSMs	More than one (children/ adolescent, adults); Educational setting	23	High confidence	December, 2020	No
(Yaacoub et al., 2021) (Rapid Review)	Social interactions, individual protection, physical environment	General population; Entertainment setting (swimming facilities)	3	Moderate	April, 2021	No
(Stratil et al., 2021) (Rapid Review)	Surveillance and individual protection	Adults; long-term care facilities	22	High	Not specified; 2021	No
(Farooq et al., 2021)	Response, Social interactions	General population; Not specified	38	Critically low	September, 2020	Yes
(Ingram et al., 2021) (Rapid Review)	Individual protection and surveillance	Not specified; healthcare and occupational setting	33	Critically low	Not specified; 2020	Yes
(Sohi et al., 2022)	Social interactions and services	General population; Not specified	27	Critically low	June, 2022	No
(Neira et al., 2021)	Response (quarantine) and social interactions	Adults; Not specified	7	Critically low	Not specified	No
(Schmidt et al., 2021)	Movement, services; social interactions	General population; educational, occupational, community, and	53	Critically low	Not specified; 2020	No

CONFIDENTIAL

		entertainment setting				
(Talic et al., 2021)	Lockdown and social interactions; services, response and social interactions; response, social interactions and lockdown; social interactions and individual protection; services and social interactions; surveillance, movement and services	General population; Not specified	72 (1 is RCT)	Critically low	Not specified; 2021	Yes
(Runacres et al., 2021)	Social distancing, quarantine, and lockdown/homestay requirements	General population; educational, occupational, and community setting	40	Low	Not specified; 2021	Yes
(Castaldelli-Maia et al., 2021)	physical distancing measures	General population; Not specified	60	Critically low	Not specified; 2020	Yes
(Ayouni et al., 2021)	Movement and Response; Lockdown, social interactions and movement; Lockdown; Surveillance and Response; Services and social interactions; Individual protection and movement	Not specified; Not specified	18	Critically low	Not specified; 2021	No

CONFIDENTIAL

(Tully et al., 2021)	Public health restrictions	General population; Not specified	14	Critically low	February, 2021	No
(Burns et al., 2021) (Rapid Review)	Response (Quarantine) and Surveillance	General population; Point of entry (Airport)	62	High	November , 2020	No
(Saulle et al., 2021)	Social distancing measures	Children/Adolescents; Not specified	42	Critically low	September , 2020	No
(Hatami et al., 2022)	Surveillance, Response, Lockdown	General population; Not specified	18	Critically low	April, 2020	No
(Saulle et al., 2021)	social distancing measures	Children/Adolescents; Not specified	64	Critically low	September , 2020	No
(Nussbaumer-Streit et al., 2020) (Rapid Review)	Lockdown, Individual protection, Response, Services, Social interactions	Individuals who had contact with confirmed or suspected cases of COVID-19, who travelled from countries with a declared outbreak, or who live in regions with high disease transmission; Educational, Healthcare, Occupational, household, point of entry, public institution	10	Critically low	June, 2020	No
(Viner et al., 2022)	Services	Children/Adolescent; Educational setting	36	Critically low	September , 2020	No

CONFIDENTIAL

(Fricke et al., 2020)	Surveillance, response, services, social interactions, individual protection	General population; Not specified	23	Critically low	July, 2020	No
(Johanna et al., 2020)	Mass testing and lockdown	General population; Not specified	18	Critically low	June, 2020	No
(Hawco et al., 2022)	Individual protection, Social interactions, Services, Movement	Pregnant women; Healthcare setting	38	Critically low	May, 2021	Yes
(Walsh et al., 2021a) (Rapid Review)	Surveillance, individual protection and physical environment; Surveillance, contact tracing, Response	General population; Community Settings (concerts or festivals, sporting events, graduation ceremony, Hajj pilgrimage, protest rally), Occupational setting and entertainment facility (business conference and a theatre performance)	11 (1 is RCT)	Moderate	June, 2021	No
(Khatib et al., 2022) (Rapid Review)	Lockdown, Response, Individual protection, Social interactions	healthcare workers; Healthcare setting	13	Critically low	Not specified	No
(Rajmil et al., 2021)	Services (School closures) and lockdown	Children/Adolescent; Not specified	22	Critically low	November, 2020	No

CONFIDENTIAL

(Girum et al., 2020)	Response and surveillance	Individuals who had contact with confirmed or suspected cases of COVID-19, who travelled from countries with a declared outbreak, or who live in regions with high disease transmission ; healthcare settings, Community Settings and national level	22	Critically low	June, 2020	No
(Lausi et al., 2021)	Stay at home (SAH) policies	General population; Not specified	19	Critically low	Not specified	No
(Alex et al., 2021)	Social interactions and lockdown	General population; Not specified	18	Critically low	January, 2021	Yes
(Bou-Karroum et al., 2021)	Movement and social interactions; services; Response; Surveillance	General population; Not specified	69	Critically low	Not specified; 2020	No
(Samji et al., 2021)	Response, social interactions	Children/Adolescent; Healthcare, Educational, home, quarantine setting	116	Critically low	February, 2021	No
(Chaabane et al., 2021) (Rapid Review)	Services (school closure) combined with Lockdown and Response	Children/Adolescent; Educational setting	10	Critically low	September, 2020	No

CONFIDENTIAL

(Iezadi et al., 2021)	Social distancing measures; Restriction on domestic mobility; response; Combination of NPHIs	General population; Community setting	35	Low	February, 2021	Yes
(Regmi et al., 2021)	Response, Services, Social interactions, Movement, Individual protection	General population; Community setting	33	Critically low	March, 2021	No
(Mbwogge, 2021)	Surveillance	General population; Community setting	35	Critically low	December, 2020	Yes
(Girum et al., 2021)	Movement and lockdown	Individuals who have contact a confirmed or suspected case of COVID-19, or individuals who live in areas with COVID-19 outbreak, or individuals considered to be high risk for COVID-19/suspected cases, or confirmed/probable cases of COVID-19 infection; Community setting	25	Critically low	Not specified; 2020	No
(Minozzi et al., 2021)	Social distancing measures (school closures and lockdowns)	Children/Adolescent; Not specified	27	Critically low	September, 2020	No

CONFIDENTIAL

± Number of included studies for the overall review and for the specific intervention

Table 59: COVID-19 epidemiological outcomes

Review	Risk and incidence	Transmission related outcomes	Mortality	Hospitalizations
(Krishnaratne et al., 2022b)<br><br>GRADE: Yes; very low certainty evidence	Evidence on multicomponent PHSMs in school settings was mixed; while most of the studies suggested PHSMs may decrease the number or proportion of cases, some showed mixed or no effects			
(Yaacoub et al., 2021)<br>GRADE: Yes; very low certainty		One study in this review found no association between implementing restrictions and COVID-19 transmission during swimming activities.		
(Stratil et al., 2021)<br>GRADE: Yes; very low to moderate	A reduction in the number of infections as a result of multicomponent infection-control measures and multicomponent cleaning and environmental hygiene measures.	A reduction in the probability of outbreaks as a result of multicomponent contact-regulating and transmission-reducing measures.	A reduction in the number of deaths as a result of multicomponent infection-control measures.	
(Ingram et al., 2021)<br>GRADE: No	Combined infection prevention and control (IPC) measures resulted in lower positivity rates (0.2% positivity; 95% CI 0–0.4%) compared to single measures.	Comprehensive IPC measures can prevent outbreaks in the workplace.		
(Talic et al., 2021)<br>GRADE: No	A reduction in COVID-19 incidence as a result of public health intervention packages.	A reduction in SARS-CoV-2 transmission as a result of public health intervention packages.	A reduction in COVID-19 mortality as a result of public health intervention packages.	

CONFIDENTIAL

(Ayouni et al., 2021)<br>GRADE: No		A reduction in the transmission of COVID-19 as a result of public health interventions and non-pharmaceutical measures, especially when combined with other measures.		
(Burns et al., 2021)<br>GRADE: Yes; moderate for one outcome and low or very low for the rest		Combining quarantine with screening at borders can be more effective in limiting the spread of COVID-19.		
(Hatami et al., 2022)<br>GRADE: No		Combining case detection and isolation with contact tracing or containment measures may be the most effective approach to manage the spread of COVID-19.		
(Nussbaumer-Streit et al., 2020)<br>GRADE: Yes; low to very low	A larger reduction of new cases as a result of combining quarantine with other prevention and control measures compared to individual measures alone.	A larger reduction of transmissions as a result of combining quarantine with other prevention and control measures compared to individual measures alone.	A larger reduction of deaths as a result of combining quarantine with other prevention and control measures compared to individual measures alone.	
(Johanna et al., 2020)<br>GRADE: No	A greater reduction of incidence as a result of combining lockdown with mass screening compared to lockdown alone.		A greater reduction of mortality rate as a result of combining lockdown with mass screening compared to lockdown alone.	
(Walsh et al., 2021a)<br>GRADE: No		The risk of SARS-CoV-2 transmission at mass gatherings can be reduced by implementing public health measures,		

CONFIDENTIAL

		however it cannot be fully eliminated. Moreover, implementing multiple measures may be more effective than single interventions.		
(Khatib et al., 2022) GRADE: Yes; very low	A reduction in the number of cases as a result of combining WASH interventions with other prevention and control measures.			
(Girum et al., 2020) GRADE: Yes; Not reported	A reduction in the number of cases as a result of quarantine, contact tracing, screening, and isolation, particularly when multiple measures were combined together.	A reduction in transmission when multiple measures were integrated together.	A reduction in deaths when multiple measures were integrated together.	
(Bou-Karroum et al., 2021) GRADE: Yes; low to very low	A reduction in the number of cases as a result of combining travel-related policies with other public health measures.	A reduction in transmission as a result of combining travel-related policies with other public health measures.	A reduction in deaths as a result of combining travel-related policies with other public health measures.	
(Iezadi et al., 2021) GRADE: No		A 4.68% (95% CI, -6.94 to -2.78) reduction in daily case growth rates and a 1.90 (95% CI, -2.23 to -1.58) reduction in the COVID-19 reproduction number as a result of Non-Pharmaceutical Public Health Interventions (NPHIs).	4.87% (95 CI, -8.34 to -1.40) decrease in daily death growth rates and 16.5% (95% CI, -19.68 to -13.32) decrease in COVID-19 daily ICU admission as a result of Non-Pharmaceutical Public Health Interventions (NPHIs).	, PHSMs were associated with a reduction in COVID-19 ICU admissions
(Regmi et al., 2021) GRADE: No		A greater reduction in COVID-19 transmission as a result of combining multiple measures compared to single		

CONFIDENTIAL

		non-pharmaceutical interventions (NPIs).		
(Mbwogge, 2021) GRADE: Yes; very low		Mass testing and contact tracing may be more effective than the conventional test and trace strategy in controlling COVID-19 transmission, especially when combined with social distancing and face coverings.		
(Girum et al., 2021) GRADE: Yes; Not reported	A reduction in COVID-19 incidence as a result of combining multiple interventions.	A reduction of transmission as a result of combining multiple interventions.<br><br>A reduction in the reproduction number as a result of combining multiple interventions.	A reduction of mortality as a result of combining multiple interventions.	

Table 60: Unintended health and socio-economic outcomes

Review	Non-COVID-19 related health outcomes
(Farooq et al., 2021) GRADE: No	**Mental health:** COVID-19 related restrictions were considered as one of the main risk factors for suicidal ideation. The pooled prevalence of suicidal ideation during COVID-19 was 12.1% (CI 9.3–15.2), which is higher than the prevalence prior to the pandemic.
(Sohi et al., 2022) GRADE: Yes; Not reported	**Substance use:** A reduction in alcohol use in some countries, and an increase in heavy episodic drinking (Alkatout et al.) and the proportion of individuals with problematic alcohol use as a result of pandemic-related policies.
(Neira et al., 2021) GRADE: No	**Nutrition:** COVID-19 preventive measures that restrict physical contact affected food intake and eating behaviors.
(Schmidt et al., 2021) GRADE: No	**Substance use:** An increase in substance use during the COVID-19 pandemic, and particularly alcohol use.
(Runacres et al., 2021) GRADE: Yes; 18 high, 39 moderate, and 7 low	**Physical activity:** An increase in sedentary time as a result of COVID-19 restrictions. Children were the most affected, with an increase of 159.5 ± 142.6 min day^{-1}.
(Castaldelli-Maia et al., 2021) GRADE: No	**Mental health:** There was no significant association between physical distancing measures and depression, while public transportation closures were associated with an increase in the prevalence of anxiety.

CONFIDENTIAL

(Tully et al., 2021)<br>GRADE: No	**Mobility:** A reduction in mobility as a result of public health restrictions during COVID-19.
(Saulle et al., 2021)<br>GRADE: No	**Health service utilization:** A reduction in access to Emergency Departments, hospital admissions, and vaccination, as well as an increase in acute cases with delayed admission.<br><br>**Violence:** An increase in domestic accidents and head trauma during COVID-19.<br><br>**Physical activity:** An increase in screen time and a reduction of physical activity among youth as a result of social distancing measures and lockdown.<br><br>**Sleep:** Sleep problems were reported among children and adolescents.<br><br>**Nutrition:** An increase in unhealthy food consumption during COVID-19.
(Saulle et al., 2021)<br>GRADE: No	**Mental health:** An increase in suicide among young individuals as a result of social distancing measures (school closures and lockdowns). Negative effects on children and adolescents' mental health (including anxiety, depression, emotional and behavioral difficulties) as a result of COVID-19 restrictive measures.<br><br>**Health service utilization:** A decrease in access to psychiatric services and admissions to the Emergency Department for psychiatric conditions.<br><br>**Violence:** A reduction in referrals and notifications of child abuse as a result of school closures and lockdowns.
(Viner et al., 2022)<br>GRADE: No	**Mental health:** School closures and social lockdown were associated with adverse mental health outcomes among children and adolescents, particularly anxiety, depression and distress. However, there was no significant association with suicide.<br><br>**Violence:** A reduction in child protection referrals during school closures and lockdown.<br><br>**Physical activity:** A decrease in physical activity levels and an increase in screen time and social media use among children and adolescents during school closures and lockdown.<br><br>**Sleep:** An increase in sleep problems among children during school closures and lockdown.<br><br>**Nutrition:** An increase in unhealthy food consumption and child obesity during school closures and lockdown.
(Fricke et al., 2020)<br>GRADE: No	**Change in incidence and mortality of diseases other than COVID-19:** A reduction in influenza burden as a result of non-pharmaceutical interventions (NPIs) targeted at COVID-19 transmission.
(Hawco et al., 2022)<br>GRADE: No	**Preterm birth:** A reduction in preterm birth at less than 37 weeks (OR 0.89, 95% CI 0.81-0.98) and 34 weeks (OR 0.56, 95% CI 0.37-0.83) for iatrogenic births and singleton pregnancies. A reduction in preterm births at less than 34 weeks in countries with COVID-19 measures above median stringency (OR 0.71, 95% CI 0.58-0.88).<br><br>**Health service utilization:** A reduction in NICU admission rates in countries with above median stringency scores (OR 0.87, 95% CI 0.78-0.97).

CONFIDENTIAL

(Rajmil et al., 2021)<br>GRADE: No	**Violence:** An increase in the risk of child abuse and violence as a result of lockdown and school closure.<br><br>**Mental health:** One study in this review showed an increase in symptoms of depression and anxiety and a decrease in life satisfaction during school closure and lockdown.
(Lausi et al., 2021)<br>GRADE: No	**Violence:** An increase in the incidents of intimate partner violence (IPV) during stay at home (SAH) policies.
(Alex et al., 2021)<br>GRADE: No	**Violence:** An increase in the incidents of domestic violence (overall mean effect size: 0.66 (CI: 0.08–1.24)) as a result of COVID-19-related restrictions (stay at home orders and lockdown orders).
(Samji et al., 2021)<br>GRADE: No	**Mental health:** An increase in depressive and anxious symptoms as well as other mental health concerns among children and adolescents as a result of COVID-19 control measures.
(Chaabane et al., 2021)<br>GRADE: Yes; low level	**Mental health:** One study in this review reported negative effects on children's mental health as a result of school closure and home quarantine.<br><br>**Health service utilization:** One study in this review reported a decrease in hospital admissions and pediatric emergency department visits for certain conditions as a result of school closure combined with lockdown measures.
(Iezadi et al., 2021)<br>GRADE: No	**Health service utilization:** A 16.5% (95% CI, -19.68 to -13.32) reduction in COVID-19 daily ICU admission as a result of Non-Pharmaceutical Public Health Interventions (NPHIs).

ii. Lockdown compared with no lockdown (n=39 reviews)

We included 39 reviews on the use of lockdown measures (compared to no lockdown); none with a search date after 2021 and with the majority reviews rated as critically low confidence on the AMSTAR instrument (table 61).

COVID-19 epidemiological outcomes were examined by six reviews (Caristia et al., 2020, Talic et al., 2021, Mendez-Brito et al., 2021, Johanna et al., 2020, Iezadi et al., 2021, Girum et al., 2020). Findings suggest that lockdown may reduce the risk, incidence, transmission rates and mortality related to COVID-19. All of the 6 reviews did not use GRADE (table 63).

Non-COVID-19 related health outcomes were examined by 34 reviews, of which only two applied GRADE (table 62).

Lockdowns may be associated with a general decrease in physical activity and increase in sedentary behaviour across several populations, including children and patients with a variety of medical conditions (Elisabeth et al., 2021, Freiberg et al., 2021a, Kharel et al., 2022, Knight et al., 2021, Rivera et al., 2021, Stephanie et al., 2021, Zaccagni et al., 2021, Rajmil et al., 2021). There were variations across population groups, from reduced outdoor play and increased indoor play and video game/screen time among children (Kourti et al., 2021b) to reduced mild physical activity among undergraduate students (Rivera et al., 2021).

CONFIDENTIAL

For nutrition-related outcomes, findings were mixed; while two reviews suggested negative associations, including increased snacking and unhealthy food consumption (Bakaloudi et al., 2021b, Mignogna et al., 2021), others suggested no association with food consumption (Elisabeth et al., 2021), or positive association with increased adherence to the Mediterranean diet (Della Valle et al., 2021). For physical activities, five reviews reported associations between lockdown and decrease in physical activity and increase in sedentary time (Elisabeth et al., 2021, Zaccagni et al., 2021, Stephanie et al., 2021, Rivera et al., 2021, Freiberg et al., 2021b).

Lockdowns may be negatively associated with mental health outcomes in adult population, including with an increased prevalence of anxiety, depression, stress and suicidal ideation (Bonati et al., 2022, Farooq et al., 2021, Panda et al., 2020, Rajmil et al., 2021, Samji et al., 2021, Wall and Dempsey, 2022, Suárez-González et al., 2021, Sideli et al., 2021). They may also be associated with children experiencing negative emotional symptoms (irritability, inattention, anxiety, depression, fear, boredom) as well as poor sleep (Camacho-Montaño et al., 2022, Panchal et al., 2021, Panda et al., 2020, Samji et al., 2021, Oliveira Carvalho et al., 2021). Lockdowns may be associated with increased prevalence of domestic violence cases (Lausi et al., 2021). In terms of substance use, varied patterns emerged during lockdown, with some countries experiencing a decrease in alcohol use but an uptick in heavy episodic drinking, potentially leading to an increase in alcohol-related issues. The observed changes in alcohol consumption and dietary habits showed significant heterogeneity, with an overall trend indicating increased alcohol consumption among adults during the lockdown period (Bakaloudi et al., 2021b, Freiberg et al., 2021b, Sohi et al., 2022).

The included reviews also found associations between COVID-19 lockdown and healthcare service utilization and health outcomes. Lockdowns may be negatively associated with interruptions in cancer screening, a decrease in emergency department visits, decreases in referrals for medical examinations for child protection and immunizations (Rajmil et al., 2021, Girum et al., 2021). They may also be associated with delays in diagnoses and increases in avoidable deaths from cancer (1 critically low-confidence review) (Alkatout et al., 2021). In contrast, lockdown may not be associated with a deterioration in glucose control, or may be associated with improvement in many glucose control parameters for patients with type 1 diabetes (Antonio et al., 2021, Garofolo et al., 2021).

Table 61: Characteristics of reviews

**Review** ***(rapid status in brackets)***	**Type of Intervention**	**Population; Setting**	**# of studies included** ***(#RCT in bracket)±***	**AMSTAR rating**	**Date last searched**	**Meta-analysis conducted**
(Panda et al., 2020)	Lockdown	Children/Adolescent; Healthcare, Educational setting	15	Critically low	August, 2020	Yes

CONFIDENTIAL

(Caristia et al., 2020) (Rapid review)	Lockdown	General population; Not Specified	19	Critically low	2020	No
(Elisabeth et al., 2021)	Lockdown	General population; Not Specified	27	Critically low	March, 2021	No
(Mignogna et al., 2021)	Lockdown	Children and Adults; Not Specified	95	Critically low	June, 2021	No
(Baumhardt et al., 2021)	Lockdown	Patients with ST-segment elevation myocardial infarction (STEMI) and/or non-ST-segment elevation myocardial infarction (NSTEMI); Health Setting	27	Low	April,2021	No
(Kourti et al., 2021b)	Lockdown	Children and/or Adolescent; Not Specified	17	Critically low	December, 2020	No
(Zaccagni et al., 2021)	Lockdown	Italian citizens; Not Specified	23	Critically low	December, 2020	No
(Farooq et al., 2021)	Lockdown	General population; Not Specified	38	Critically low	September, 2020	No
(Rezwanul Hasan et al., 2021)	Lockdown	Not Specified; Not Specified	35	Critically low	2020	No
(Sohi et al., 2022)	Lockdown	General population; Not Specified	27	Critically low	June, 2022	No
(Rivera et al., 2021)	Lockdown	Children and/or Adolescent; Not Specified	7	Low	January, 2021	No

CONFIDENTIAL

(Mendez-Brito et al., 2021)	Lockdown	General population; Healthcare, educational, occupational, Point of entry, public, and community setting	34	Critically low	March, 2021	No
(Talic et al., 2021)	Lockdown	General population; Not Specified	72 (1 is RCT)	Critically low	2021	Yes
(Chang et al., 2021)	Lockdown	Children and/or Adolescent; Not Specified	12	Critically low	2021	Yes
(Wall and Dempsey, 2022)	Lockdown	Perinatal women; Not Specified	16	Low	May, 2021	No
(Suárez-González et al., 2021) (Rapid review)	Lockdown	People with any kind of dementia or mild cognitive impairment; Not Specified	15	Low	February, 2021	No
(Stephanie et al., 2021)	Lockdown	General population; Not Specified	66	Low	June, 2020	No
(Freiberg et al., 2021b) (Rapid review)	Lockdown	General population; Educational, Healthcare, occupational, and entertainment setting	33	Critically low	March, 2020	No
(Oliveira Carvalho et al., 2021)	Lockdown	University students; Not Specified	13	Critically low	June, 2021	Yes
(Della Valle et al., 2021)	Lockdown	General population; Not Specified	12	Critically low	2020	No
(Bakaloudi et al., 2021a)	Lockdown	Adults and adolescents (>16 years old); Not Specified	36	Critically low	December, 2020	Yes

CONFIDENTIAL

(Knight et al., 2021)	Lockdown	Adults; Not Specified	64	Critically low	January, 2021	No
(Johanna et al., 2020)	Lockdown	General population; Not Specified	18	Critically low	June, 2020	No
(Sideli et al., 2021)	Lockdown	People with eating disorders; Not Specified	26	Critically low	January, 2021	Yes
(Panchal et al., 2021)	Lockdown	Children and/or Adolescent; Not Specified	61	Critically low	April, 2021	No
(Kharel et al., 2022)	Lockdown	Children and/or Adolescent; Not Specified	71	Low	2021	No
(Rajmil et al., 2021)	Lockdown	Children and/or Adolescent; Not Specified	22	Critically low	Novembe r, 2021	No
(Lausi et al., 2021)	Lockdown	General population; Not Specified	19	Critically low	Not specified	No
(Brakspear et al., 2022)	Lockdown	Children and/or Adolescent; Not Specified	16	Low	Decembe r 2021	No
(Alkatout et al., 2021)	Lockdown	General population; Not Specified	10	Critically low	January, 2021	No
(Antonio et al., 2021)	Lockdown	People with type 1 (T1DM) and type 2 (T2DM) diabetes; Not Specified	36	Critically low	March, 2021	Yes
(Bakaloudi et al., 2021b)	Lockdown	Adolescent (≥16 years) or adults; Not Specified	32	Critically low	Novembe r, 2020	No
(Garofolo et al., 2021)	Lockdown	People with type 1 (T1DM); Not Specified	17	Critically low	February, 2021	Yes

CONFIDENTIAL

(Camacho-Montaño et al., 2022)	Lockdown	Children; Household Setting	8	Critically low	March, 2021	No
(Samji et al., 2021)	Lockdown	Children and/or Adolescent; Healthcare, Educational, household, and quarantine settings	116	Critically low	February, 2021	No
(Kourti et al., 2021a)	Lockdown	General population; household settings	32	Critically low	2020	No
(Iezadi et al., 2021)	Lockdown	General population; Community setting	35	Low	February, 2021	Yes
(Bonati et al., 2022)	Lockdown	General population; Community setting	105	Critically low	April, 2021	No
(Girum et al., 2021)	Lockdown	Individuals who have contact a confirmed or suspected case of COVID-19, or individuals who live in areas with COVID-19 outbreak; or individuals considered to be high risk for COVID-19/suspected cases, or confirmed/probable cases of COVID-19 infection; Community setting	25	Critically low	2020	No

±Number of included studies for the overall review and for the specific intervention

Table 62: COVID-19 Transmission-related Outcomes

Review	Risk and incidence	Transmission related outcomes	Mortality	Hospitalizations

CONFIDENTIAL

(Caristia et al., 2020) GRADE: No	A daily reduction in infection rates ranging from -0.6% (in Sweden) to -11.3% (in Hubei and Guangdong provinces) as a result of lockdown.	An 82% reduction of reproductive number (Rt), ranging from a posterior Rt of 0.44 (95%CI 0.26-0.61) for Norway to a posterior Rt of 0.82 (95%CI 0.73- 0.93) for Belgium as a result of lockdown.		
(Mendez-Brito et al., 2021) GRADE: No	A reduction in COVID-19 cases as a result of lockdown measures.	A reduction in the reproduction number (Rt) and case growth rates as a result of lockdown measures.		
(Talic et al., 2021) GRADE: No	A reduction in new cases of covid-19 in countries that implemented universal lockdown compared to countries that did not (β coefficient −235.8 (standard error −11.04), P<0.01).	A reduction in SARS-CoV-2 transmission during the first few months of the pandemic as a result of universal lockdown.<br><br>A decrease in the reproductive number (R0) with variation across countries.	A reduction in COVID-19 mortality as a result of universal lockdown.	
(Johanna et al., 2020) GRADE: No	A reduction in COVID-19 incidence as a result of lockdown.	A reduction in COVID-19 transmission as a result of lockdown.	A reduction in COVID-19 mortality rate as a result of lockdown.	
(Iezadi et al., 2021) GRADE: No	A maximum reduction of 8.85% of incidence rate in Spain as a result of lockdown. (1 Study)		A maximum reduction of 19.00% for death rates in Spain as a result of lockdown. (1 Study)	A maximum reduction of 10.20% for hospital admission rates and 20.71% for ICU admission rates in Spain as a result of lockdown. (1 Study)
(Girum et al., 2020) GRADE: Yes (Not reported)			An 80% reduction in contacts and a decrease in the initial reproductive number from 3.0 [95% CI: 2.8, 3.2] to 0.68	A strong reduction in the number of hospitalizations and intensive care patients as a result of lockdown. (1 Study)

CONFIDENTIAL

			[95% CI: 0.62–0.73]) as a result of lockdown strategy. (1 Study)	

Table 63: Unintended health and socio-economic outcomes

Review	Non-COVID-19 related outcomes	Socio-economic outcomes	Other: Environmental outcomes
(Panda et al., 2020) GRADE: Yes; Moderate	**Mental health:** Percentages of children experiencing anxiety, depression, irritability, and inattention were 34.5%, 41.7%, 42.3%, and 30.8% respectively. The pandemic and quarantine negatively affected the behavior and psychological well-being of 79.4% of children. Additionally, at least 22.5% of children had a significant fear of COVID-19, while 35.2% and 21.3% experienced boredom and sleep disturbances. Similarly, 52.3% of caregivers developed anxiety, and 27.4% experienced depression while in isolation with children.		
(Elisabeth et al., 2021) GRADE: No;	**Nutrition:** Six of 10 papers reported no significant changes in quantity or quality of food consumption; 4 out of 10 studies reported unfavorable changes in diet during lockdown. **Physical activity:** Thirteen of 22 studies reported a decrease in physical activity or an increase in sedentary time; 9 studies reported no major changes in activity levels during lockdown.		
(Mignogna et al., 2021) GRADE: No;	**Nutrition:** An increase in food intake, number of daily meals and snacking during lockdown. An increased consumption in foods such as dairy products, fruits and vegetables, legumes, cereals, olive oil and unhealthy food (e.g., snacks and sweets). A decrease intake in foods such as fish, red and processed meat. An improved diet quality in Europe, especially among Mediterranean countries, with the exception of France. A switch to poor nutrient patterns in Colombia and Saudi Arabia.		

CONFIDENTIAL

(Baumhardt et al., 2021) GRADE: No;	**Hospitalization:** A significant decrease in the number of hospital admissions of patients with myocardial infarction during the lockdown (incidence rate ratio [IRR] = 0.516 [0.403; 0.660], I2 = 98%). This was true both for patients with STEMI (IRR = 0.620 [0.514; 0.746], I2 = 96%) and for patients with NSTEMI (IRR = 0.454 [0.354; 0.584], I2 = 96%).		
(Kourti et al., 2021b) GRADE: No;		**Early child development:** A change in children's play habits, with reduction in outdoor play and an increase in indoor play and in videogames-screen time during lockdown (play is a key factor for children's healthy psychological, emotional, social, and cognitive development)	
(Zaccagni et al., 2021) GRADE: No;	**Physical activity:** A significant reduction in the amount of performed physical activity in both the general population and in individuals with chronic conditions during lockdown.		
(Farooq et al., 2021) GRADE: No;	**Mental health:** a pooled prevalence of suicidal ideation was 12.1% (CI 9.3-15.2). Reported risk factors for suicidal ideations were: include low social support, high physical and mental exhaustion and poorer self-reported physical health in frontline medical workers, sleep disturbances, quarantine and exhaustion, loneliness, and mental health difficulties.		
(Rezwanul Hasan et al., 2021) GRADE: No;			**Air quality:** A substantial reduction in air pollution varied by region and period, with traffic-related NO2 exhibiting the largest decrease as a result of lockdown measures in China. The reported

CONFIDENTIAL

			reductions in air pollution were observed in urban, industrial and highly populated areas of China. Yet, the levels of pollutants in numerous areas continued to surpass the World Health Organization's (WHO) recommended average limit over a 24-hour period.
(Sohi et al., 2022) GRADE: Yes; (Results of the GRADE are not reported)	**Substance Use:** A decline in alcohol use in some countries and an increase in heavy episodic drinking and the prevalence of individuals with alcohol-related issues. Preliminary observations regarding shifts in alcohol consumption reveal significant heterogeneity.		
(Rivera et al., 2021) GRADE: No;	**Physical activity:** A significant reduction in mild physical activity (i.e., walking) among undergraduate students but not among graduate. A significant increase in sedentary time (i.e., sitting time) in undergraduate but not graduate students. Results also revealed that physical activity (moderate and/or vigorous) increased or did not change among students who were more sedentary prior to lockdown whereas, it decreased for those who were sedentary prior to lockdown.		
(Chang et al., 2021) GRADE: No;	**Nutrition: A** significant body weight gain during lockdown was observed by the pooled results (MD 2.67, 95% CI 2.12-3.23; $p < 0.00001$). No significant change in the BMI of children with comorbidities or obesity was observed. A significant increase in the BMI of the general population was observed during lockdown (MD 0.94, 95% CI 0.32-1.56; $p = 0.003$). Heterogeneity was high ($I2 = 84\%$). An increase in rates of obesity (OR 1.23, 95% CI 1.10-1.37; $p = 0.0002$) and overweight (OR 1.17, 95% CI 1.06-1.29; $p = 0.001$) was observed in terms of changes in weight classification.		

CONFIDENTIAL

(Wall and Dempsey, 2022) GRADE: No;	**Mental health:** A negative impact on perinatal mental health as a result of COVID-19 lockdowns. The reported risk factors for the reported impact include resilience, educational attainment, trimester, and ethnicity.		
(Suárez-González et al., 2021) GRADE: No;	**Mental health:** Among the 15 included studies, 14 studies reported worsening or new onset of behavioral and psychological symptoms, 9 studies reported changes in cognition and 6 studies reported a functional decline in daily activities as a result of lockdown.		
(Stephanie et al., 2021) GRADE: No;	**Physical activity:** A reduction in physical activity and increase in sedentary behaviors across several populations, including children and patients with a variety of medical conditions was observed during lockdown.		
(Freiberg et al., 2021b) GRADE: No;	**Physical activity:** A reduction in physical activity and increase in sedentary behavior among all age groups during the COVID-19 lockdown.<br>**Substance Use:** An increase in alcohol consumption and food intake and the deterioration of dietary quality was observed among adults during lockdown.		
(Oliveira Carvalho et al., 2021) GRADE: No;	**Mental health:** An increased rate of emotional symptoms in students was observed with an overall pooled prevalence rate of 55% (95% CI: 45-64%) for anxiety, 63% (95% CI: 52-73%) for depression and 62% (95% CI: 43-79%) for stress as a result of lockdown. The reported factors leading to the increase include Isolation, reduced social contact as well as duration of quarantine and restrictions.		
(Della Valle et al., 2021) GRADE: No;	**Nutrition:** An increase in the adherence to Mediterranean diet (MD) was observed in 6 out of the 7 included studies as a result of lockdown (rate of change of high-adherence to MD ranged between +3.3% and +21.9%). Results showed that the consumption of MD food items increased, yet it is subject to heterogeneity.		

CONFIDENTIAL

(Bakaloudi et al., 2021a) GRADE: No;	**Nutrition:** An increase in body weight was reported in a range 11.1-72.4% of individuals, whereas 7.2-51.4% of individuals reported weight loss. A significant increase in body weight was observed in the post-lockdown period with a weighted mean between-group difference (WMD) 1.57 (95% CI 1.01 to 2.14) and higher BMI 0.31 WMD (95% CI, 0.17 to 0.45) was observed before the lockdown period.		
(Knight et al., 2021) GRADE: No;	**Physical activity:** No effect of lockdown policy or COVID-19 restrictions on physical activity was observed.		
(Sideli et al., 2021) GRADE: No;	**Mental Health:** More than half of the participants experienced depression and anxiety.<br>**Nutrition:** A symptomatic deterioration in eating disorders (EDs) was observed with a pooled prevalence of 65% (95% CI [48,81], k = 10). An increased weight in obesity was observed with a 52% (95% CI [25, 78], k = 4). Shape, eating concerns and increased thinking about exercising were reported in at least 75% of the individuals with EDs. The combined analysis of longitudinal studies revealed no significant differences in Body Mass Index and ED symptoms between pre-pandemic levels to the first lockdown phase, whereas a limited number of studies suggested elevated levels distress, particularly among individuals with anorexia nervosa.		
(Panchal et al., 2021) GRADE: No;	**Mental health:** Increase in prevalence of psychological distress as a result of COVID-19 lockdown. Anxiety symptoms and depression symptoms were common in the included studies, with prevalence ranging from1.8% to 49.5% and 2.2% to 63.8%, respectively. Irritability (range = 16.7-73.2%) and anger (range = 30.0-51.3%), were frequently reported by children and adolescents. Special needs, the presence of mental disorders before the lockdown and excessive media exposure, were significant risk		

CONFIDENTIAL

	factors for anxiety. Parent-child communication was protective for anxiety and depression.		
(Kharel et al., 2022)<br>GRADE: No;	**Physical activity:** A reduction in physical activity and an increase in screen time and sleep hours were observed among children and adolescents as a result of lockdown. These changes were more pronounced in Children and adolescents facing strict lockdowns compared to those under mild restrictions.		
(Rajmil et al., 2021)<br>GRADE: No;	**Mental Health:** An increase in depressive symptoms and decrease in life satisfaction as a result of lockdown were observed in studies from Australia, Spain and China.<br>**Nutrition** An increase in unhealthy food consumption was observed as a result of lockdown.<br>**Physical activity:** A decrease in physical activity was observed as a result of lockdown.<br>**Health services utilization:** A decrease in emergency department visits in four countries, an increase in child mortality in Cameroon, a significant decline of over 50% in immunizations administered in Pakistan, and a substantial drop of 39% in child protection medical examination referrals in the UK during 2020 compared to previous years.		
(Lausi et al., 2021)<br>GRADE: No;	**Violence:** An increase in intimate partner violence (IPV) on women due to increase time spent together was observed as a result of forced cohabitation.		
(Brakspear et al., 2022)<br>GRADE: No;	**Nutrition:** A pattern towards healthier eating behaviors among children and adolescents during the COVID-19 lockdown observed. a tendency towards more unhealthy eating behaviors among young people from lower socioeconomic groups was observed. An association was found between mood difficulties and greater alterations in eating patterns.		

CONFIDENTIAL

(Alkatout et al., 2021) GRADE: No;	**Change in incidence and mortality of diseases other than COVID-19:** An interruption in the Cancer screening programs since the onset of the COVID-19 disease. Consequences include delayed diagnosis and marked increases in the numbers of avoidable cancer deaths.		
(Antonio et al., 2021) GRADE: No;	**Change in incidence and mortality of diseases other than COVID-19:** No significant change in Hba1c was observed in T1DM (WMD - 1.474 [-3.26; 0.31] mmol/mol, I2 = 93.9) and T2DM (WMD - 1.257 - 3.91; 1.39 mmol/mol, I2 = 98.3%) as a result of lockdown. A significant increase in Time in range (TIR) during and after lockdown (WMD: 2.73 1.47; 4.23 %, I2 = 81% and 3.73 [1.13; 5.33] %, I2 = 85%, respectively).		
(Bakaloudi et al., 2021b) GRADE: No;	**Nutrition:** A significant increase in snacking (18.9 - 45.1%) and alcohol consumption, and a decrease in fast food (15.0-41.3%) and ordered food (33.9%) was observed.<br>**Substance Use:** A significant increase in alcohol consumption, was observed.		
(Garofolo et al., 2021) GRADE: No;	**Change in incidence and mortality of diseases other than COVID-19:** an increase in Time in Range (TIR) within the range of 70-180 mg/dl by 3.05% in individuals with type 1 diabetes as a result of lockdown. A decline in Time Above Range (Freiberg et al.) above 180 mg/dl and above 250 mg/dl by 3.39% and 1.96% respectively. Both Time Below Range (TBR) below 70 mg/dl and below 54 mg/dl remained unchanged. Mean Blood Glucose (MBG) slightly decreased by 5.40 mg/dl along with a reduction in the coefficient of variation (%CV). Pooled estimated A1c (ea1c) and Glucose Management Indicator (Regmi et al.) decreased by 0.18% and 0.15% respectively. The use of sensors showed a slight, but not statistically significant, reduction during the lockdown period.		

CONFIDENTIAL

(Camacho-Montaño et al., 2022) GRADE: No;	**Sleep:** An increase in duration of sleep time, sleep latency, and daytime sleepiness were observed as a result of lockdown.		
(Samji et al., 2021) GRADE: No;	**Mental health:** A high prevalence of COVID-19-related fear, increase depressive and anxious symptoms were observed among children and adolescents. A high prevalence of negative mental health was observed among older adolescents, girls, children and adolescents living with neurodiversity and/or chronic physical conditions. An increase mental health deterioration was observed among children and adolescents. Reported protective factors for mental health include physical exercise, access to entertainment, positive familial relationships, and social support.		
(Kourti et al., 2021a) GRADE: No;	**Violence:** An increased prevalence of domestic violence cases was observed as a result of COVID-19 lockdown. An increase in child maltreatment and abuse cases were estimated in children as reported by specialists, whereas rate of police and social services' reports has declined during the COVID-19 pandemic		
(Bonati et al., 2022) GRADE: No;	**Mental health:** An increase in the sample that met the criteria for the general anxiety disorder (GAD) from 2% before the pandemic to 12% accordingly to a German study.		
(Girum et al., 2021) GRADE: No;	**Hospitalizations:** A reduction in the number of hospitalizations and intensive care patients as a result of lockdown according to a study. An 80% reduction in contacts.		

CONFIDENTIAL

REFERENCES

ALEX, R. P., ALEX, R. P., WESLEY, G. J., ERIN, J., CATHERINE, K. & FELICIA MARIA, K. 2021. Evidence from a systematic review and meta-analysis: Domestic Violence during the COVID-19 Pandemic. *Journal of Criminal Justice*.

ALKATOUT, I., BIEBL, M., MOMENIMOVAHED, Z., GIOVANNUCCI, E., HADAVANDSIRI, F., SALEHINIYA, H. & ALLAHQOLI, L. 2021. Has COVID-19 Affected Cancer Screening Programs? A Systematic Review. *Frontiers in oncology,* 11**,** 675038.

ANTONIO, S. G., CHIARA, D. P., ILARIA, D., MATTEO, M. & EDOARDO, M. 2021. Glucose control in diabetes during home confinement for the first pandemic wave of COVID-19: a meta-analysis of observational studies. *Acta diabetologica*.

ASÍN-IZQUIERDO, I., RUIZ-RANZ, E. & ARÉVALO-BAEZA, M. 2022. The Physiological Effects of Face Masks During Exercise Worn Due to COVID-19: A Systematic Review. *Sports health***,** 19417381221084661.

AYOUNI, I., MAATOUG, J., DHOUIB, W., ZAMMIT, N., FREDJ, S. B., GHAMMAM, R. & GHANNEM, H. 2021. Effective public health measures to mitigate the spread of COVID-19: a systematic review. *BMC public health,* 21**,** 1015.

BAKALOUDI, D. R., BARAZZONI, R., BISCHOFF, S. C., BREDA, J., WICKRAMASINGHE, K. & CHOURDAKIS, M. 2021a. Impact of the first COVID-19 lockdown on body weight: A combined systematic review and a meta-analysis. *Clinical nutrition (Edinburgh, Scotland)*.

BAKALOUDI, D. R., JEYAKUMAR, D. T., JAYAWARDENA, R. & CHOURDAKIS, M. 2021b. The impact of COVID-19 lockdown on snacking habits, fast-food and alcohol consumption: A systematic review of the evidence. *Clinical nutrition (Edinburgh, Scotland)*.

BAUMHARDT, M., DREYHAUPT, J., WINSAUER, C., STUHLER, L., THIESSEN, K., STEPHAN, T., MARKOVIC, S., ROTTBAUER, W., IMHOF, A. & RATTKA, M. 2021. The Effect of the Lockdown on Patients With Myocardial Infarction During the COVID- 19 Pandemic-a Systematic Review and Meta-Analysis. *Deutsches Arzteblatt international,* 118**,** 253.

BONATI, M., CAMPI, R. & SEGRE, G. 2022. Psychological impact of the quarantine during the COVID-19 pandemic on the general European adult population: a systematic review of the evidence. *Epidemiology and psychiatric sciences,* 31**,** e27.

BOU-KARROUM, L., KHABSA, J., JABBOUR, M., HILAL, N., HAIDAR, Z., ABI KHALIL, P., KHALEK, R. A., ASSAF, J., HONEIN-ABOUHAIDAR, G., SAMRA, C. A., HNEINY, L., AL-AWLAQI, S., HANEFELD, J., EL-JARDALI, F., AKL, E. A. & BCHERAOUI, C. E. 2021. Public Health Effects of Travel-Related Policies on the COVID-19 Pandemic: A Mixed-Methods Systematic Review. *The Journal of infection*.

BRAKSPEAR, L., BOULES, D., NICHOLLS, D. & BURMESTER, V. 2022. The Impact of COVID-19-Related Living Restrictions on Eating Behaviours in Children and Adolescents: A Systematic Review. *Nutrients,* 14.

BURNS, J., MOVSISYAN, A., STRATIL, J. M., BIALLAS, R. L., COENEN, M., EMMERT-FEES, K. M., GEFFERT, K., HOFFMANN, S., HORSTICK, O., LAXY, M., KLINGER, C., KRATZER, S., LITWIN, T., NORRIS, S., PFADENHAUER, L. M., VON PHILIPSBORN, P., SELL, K., STADELMAIER, J., VERBOOM, B., VOSS, S., WABNITZ, K. & REHFUESS, E. 2021. International travel-related

CONFIDENTIAL

control measures to contain the COVID-19 pandemic: a rapid review. *The Cochrane database of systematic reviews,* 3**,** CD013717.
BYAMBASUREN, O., BELLER, E., CLARK, J., COLLIGNON, P. & GLASZIOU, P. 2021. The effect of eye protection on SARS-CoV-2 transmission: a systematic review. *Antimicrobial resistance and infection control,* 10**,** 156.
CAMACHO-MONTAÑO, L. R., IRANZO, A., MARTÍNEZ-PIÉDROLA, R. M., CAMACHO-MONTAÑO, L. M., HUERTAS-HOYAS, E., SERRADA-TEJEDA, S., GARCÍA-BRAVO, C. & DE HEREDIA-TORRES, M. P. 2022. Effects of COVID-19 home confinement on sleep in children: A systematic review. *Sleep medicine reviews,* 62**,** 101596.
CARDWELL, K., JORDAN, K., BYRNE, P., HARRINGTON, P., RYAN, M., O'NEILL, M., CARDWELL, K., SMITH, S. M. & RYAN, M. 2021. The effectiveness of non-contact thermal screening as a means of identifying cases of Covid-19: a rapid review of the evidence. *Rev. Med. Virol.,* 31**,** e2192.
CARISTIA, S., FERRANTI, M., SKRAMI, E., RAFFETTI, E., PIERANNUNZIO, D., PALLADINO, R., CARLE, F., SARACCI, R., BADALONI, C., BARONE-ADESI, F., BELLEUDI, V., ANCONA, C. & LOCKDOWNS, A. I. E. W. G. O. T. E. O. T. E. O. 2020. Effect of national and local lockdowns on the control of COVID-19 pandemic: a rapid review. *Epidemiologia e prevenzione,* 44**,** 60-68.
CASTALDELLI-MAIA, J. M., MARZIALI, M. E., LU, Z. & MARTINS, S. S. 2021. Investigating the effect of national government physical distancing measures on depression and anxiety during the COVID-19 pandemic through meta-analysis and meta-regression. *Psychological medicine,* 51**,** 1-46.
CAVICCHIOLI, M., FERRUCCI, R., GUIDETTI, M., CANEVINI, M. P., PRAVETTONI, G. & GALLI, F. 2021. What Will Be the Impact of the Covid-19 Quarantine on Psychological Distress? Considerations Based on a Systematic Review of Pandemic Outbreaks. *Healthcare (Basel, Switzerland),* 9.
CHAABANE, S., DORAISWAMY, S., CHAABNA, K., MAMTANI, R. & CHEEMA, S. 2021. The Impact of COVID-19 School Closure on Child and Adolescent Health: A Rapid Systematic Review. *Children (Basel, Switzerland),* 8.
CHAI, J., XU, H., AN, N., ZHANG, P., LIU, F., HE, S., HU, N., XIAO, X., CUI, Y. & LI, Y. 2021. The Prevalence of Mental Problems for Chinese Children and Adolescents During COVID-19 in China: A Systematic Review and Meta-Analysis. *Frontiers in pediatrics,* 9**,** 661796.
CHANG, T. H., CHEN, Y. C., CHEN, W. Y., CHEN, C. Y., HSU, W. Y., CHOU, Y. & CHANG, Y. H. 2021. Weight Gain Associated with COVID-19 Lockdown in Children and Adolescents: A Systematic Review and Meta-Analysis. *Nutrients,* 13.
DANIELA, R., SUSAN, A., BARBARA, C., LAURA, C., SUSAN, S., KIERAN, A. W., PAUL, G. C., LINDA, D., TINA, B., SUSAN, M. S., MÁIRE, A. C., PATRICIA, H., MÁIRÍN, R. & MICHELLE, O. N. 2020. Effectiveness of face masks worn in community settings at reducing the transmission of SARS-CoV-2: A rapid review. 3.
DELLA VALLE, P. G., MOSCONI, G., NUCCI, D., VIGEZZI, G. P., GENTILE, L., GIANFREDI, V., BONACCIO, M., GIANFAGNA, F., SIGNORELLI, C., IACOVIELLO, L. & ODONE, A. 2021. Adherence to the Mediterranean Diet during the COVID-19 national lockdowns: a systematic review of observational studies. *Acta bio-medica : Atenei Parmensis,* 92**,** e2021440.
DESYE, B. 2021. COVID-19 Pandemic and Water, Sanitation, and Hygiene: Impacts, Challenges, and Mitigation Strategies. *Environmental health insights,* 15**,** 11786302211029447.
ELISABETH, A. L., KARLEN, S. B. & MAGKOS, F. 2021. The Effect of COVID-19-related Lockdowns on Diet and Physical Activity in Older Adults: A Systematic Review. *Aging and disease,* 12**,** 1935-1947.
FAROOQ, S., TUNMORE, J., ALI, W. & AYUB, M. 2021. Suicide, self-harm and suicidal ideation during COVID-19: A systematic review. *Psychiatry research,* 306**,** 114228.

CONFIDENTIAL

FORD, N., HOLMER, H. K., CHOU, R., VILLENEUVE, P. J., BALLER, A., VAN KERKHOVE, M. & ALLEGRANZI, B. 2021. Mask use in community settings in the context of COVID-19: A systematic review of ecological data. *EClinicalMedicine,* 38**,** 101024.
FREIBERG, A., HORVATH, K., HAHNE, T. M., DRÖSSLER, S., KÄMPF, D., SPURA, A., BUHS, B., REIBLING, N., DE BOCK, F., APFELBACHER, C. & SEIDLER, A. 2021a. [Impact of wearing face masks in public to prevent infectious diseases on the psychosocial development in children and adolescents: a systematic review]. *Bundesgesundheitsblatt, Gesundheitsforschung, Gesundheitsschutz*.
FREIBERG, A., SCHUBERT, M., ROMERO STARKE, K., HEGEWALD, J. & SEIDLER, A. 2021b. A Rapid Review on the Influence of COVID-19 Lockdown and Quarantine Measures on Modifiable Cardiovascular Risk Factors in the General Population. *International journal of environmental research and public health,* 18.
FRICKE, L. M., GLÖCKNER, S., DREIER, M. & LANGE, B. 2020. Impact of non-pharmaceutical interventions targeted at COVID-19 pandemic on influenza burden - a systematic review. *The Journal of infection,* 82**,** 1-35.
GALANIS, P., VRAKA, I., FRAGKOU, D., BILALI, A. & KAITELIDOU, D. 2021. Impact of personal protective equipment use on health care workers' physical health during the COVID-19 pandemic: a systematic review and meta-analysis. *American journal of infection control*.
GAROFOLO, M., ARAGONA, M., RODIA, C., FALCETTA, P., BERTOLOTTO, A., CAMPI, F., DEL PRATO, S. & PENNO, G. 2021. Glycaemic control during the lockdown for COVID-19 in adults with type 1 diabetes: A meta-analysis of observational studies. *Diabetes research and clinical practice***,** 109066.
GIRUM, T., LENTIRO, K., GEREMEW, M., MIGORA, B. & SHEWAMARE, S. 2020. Global strategies and effectiveness for COVID-19 prevention through contact tracing, screening, quarantine, and isolation: a systematic review. *Trop. Med. Health,* 48**,** 91.
GIRUM, T., LENTIRO, K., GEREMEW, M., MIGORA, B., SHEWAMARE, S. & SHIMBRE, M. S. 2021. Optimal strategies for COVID-19 prevention from global evidence achieved through social distancing, stay at home, travel restriction and lockdown: a systematic review. *Archives of public health = Archives belges de sante publique,* 79**,** 150.
GREKOUSIS, G. & LIU, Y. 2021. Digital contact tracing, community uptake, and proximity awareness technology to fight COVID-19: a systematic review. *Sustainable cities and society,* 71**,** 102995.
GRÉPIN, K. A., HO, T. L., LIU, Z., MARION, S., PIPER, J., WORSNOP, C. Z. & LEE, K. 2021. Evidence of the effectiveness of travel-related measures during the early phase of the COVID-19 pandemic: a rapid systematic review. *BMJ global health,* 6.
HAMMERSTEIN, S., KÖNIG, C., DREISÖRNER, T. & FREY, A. 2021. Effects of COVID-19-Related School Closures on Student Achievement-A Systematic Review. *Frontiers in psychology,* 12**,** 746289.
HATAMI, H., QADERI, S., SHAH, J., REZAEIAN, A. R., FARSI, Y., ALINASAB, F., QADERI, F., KHOSRAVI, A., BAZGIR, N. & SHAH, A. 2022. COVID-19: National Pandemic Management Strategies and their Efficacies and Impacts on the Number of Secondary Cases and Prognosis: A Systematic Review. *International journal of preventive medicine,* 13**,** 100.
HAWCO, S., ROLNIK, D. L., WOOLNER, A., CAMERON, N. J., WYNESS, V., MOL, B. W. & BLACK, M. 2022. The impact of mitigation measures on perinatal outcomes during the first nine months of the COVID-19 pandemic: A systematic review with meta-analysis. *European journal of obstetrics, gynecology, and reproductive biology,* 274**,** 117-127.
HOSSAIN, A. D., JAROLIMOVA, J., ELNAIEM, A., HUANG, C. X., RICHTERMAN, A. & IVERS, L. C. 2022. Effectiveness of contact tracing in the control of infectious diseases: a systematic review. *The Lancet. Public health*.
HUGELIUS, K., HARADA, N. & MARUTANI, M. 2021. Consequences of visiting restrictions during the COVID-19 pandemic: An integrative review. *International journal of nursing studies,* 121**,** 104000.

CONFIDENTIAL

IEZADI, S., GHOLIPOUR, K., AZAMI-AGHDASH, S., GHIASI, A., REZAPOUR, A., POURASGHARI, H. & PASHAZADEH, F. 2021. Effectiveness of non-pharmaceutical public health interventions against COVID-19: A systematic review and meta-analysis. *PloS one,* 16**,** e0260371.
INGRAM, C., DOWNEY, V., ROE, M., CHEN, Y., ARCHIBALD, M., KALLAS, K. A., KUMAR, J., NAUGHTON, P., UTEH, C. O., ROJAS-CHAVES, A., SHRESTHA, S., SYED, S., CLÉIRIGH BÜTTNER, F., BUGGY, C. & PERROTTA, C. 2021. COVID-19 Prevention and Control Measures in Workplace Settings: A Rapid Review and Meta-Analysis. *International journal of environmental research and public health,* 18.
JABS, J. M., SCHWABE, A., WOLLKOPF, A. D., GEBEL, B., STADELMAIER, J., ERDMANN, S., RADICKE, F., GRUNDMANN, H., KRAMER, A., MONSEF, I., RÜCKER, G., RUPP, J., SCHEITHAUER, S., SCHMUCKER, C., SIMON, A. & MUTTERS, N. T. 2022. The role of routine SARS-CoV-2 screening of healthcare-workers in acute care hospitals in 2020: a systematic review and meta-analysis. *BMC infectious diseases,* 22**,** 587.
JENNISKENS, K., BOOTSMA, M. C. J., DAMEN, J., OERBEKKE, M. S., VERNOOIJ, R. W. M., SPIJKER, R., MOONS, K. G. M., KRETZSCHMAR, M. E. E. & HOOFT, L. 2021. Effectiveness of contact tracing apps for SARS-CoV-2: a rapid systematic review. *BMJ open,* 11**,** e050519.
JIN, Y., SUN, T., ZHENG, P. & AN, J. 2021. Mass quarantine and mental health during COVID-19: A meta-analysis. *Journal of affective disorders,* 295**,** 1335-1346.
JOHANNA, N., CITRAWIJAYA, H. & WANGGE, G. 2020. Mass screening vs lockdown vs combination of both to control COVID-19: A systematic review. *Journal of public health research,* 9**,** 2011.
KHAREL, M., SAKAMOTO, J. L., CARANDANG, R. R., ULAMBAYAR, S., SHIBANUMA, A., YAROTSKAYA, E., BASARGINA, M. & JIMBA, M. 2022. Impact of COVID-19 pandemic lockdown on movement behaviours of children and adolescents: a systematic review. *BMJ global health,* 7.
KHATIB, M. N., SINHA, A., MISHRA, G., QUAZI, S. Z., GAIDHANE, S., SAXENA, D., GAIDHANE, A. M., BHARDWAJ, P., SAWLESHWARKAR, S. & ZAHIRUDDIN, Q. S. 2022. WASH to control COVID-19: A rapid review. *Frontiers in public health,* 10**,** 976423.
KHOSRAVIZADEH, O., AHADINEZHAD, B., MALEKI, A., NAJAFPOUR, Z. & GOLMOHAMMADI, R. 2021. Social distance capacity to control the COVID-19 pandemic: A systematic review on time series analysis. *The International journal of risk & safety in medicine*.
KNIGHT, R. L., MCNARRY, M. A., SHEERAN, L., RUNACRES, A. W., THATCHER, R., SHELLEY, J. & MACKINTOSH, K. A. 2021. Moving Forward: Understanding Correlates of Physical Activity and Sedentary Behaviour during COVID-19-An Integrative Review and Socioecological Approach. *International journal of environmental research and public health,* 18.
KOURTI, A., STAVRIDOU, A., PANAGOULI, E., PSALTOPOULOU, T., SPILIOPOULOU, C., TSOLIA, M., SERGENTANIS, T. N. & TSITSIKA, A. 2021a. Domestic Violence During the COVID-19 Pandemic: A Systematic Review. *Trauma, violence & abuse***,** 15248380211038690.
KOURTI, A., STAVRIDOU, A., PANAGOULI, E., PSALTOPOULOU, T., TSOLIA, M., SERGENTANIS, T. N. & TSITSIKA, A. 2021b. Play Behaviors in Children during the COVID-19 Pandemic: A Review of the Literature. *Children (Basel, Switzerland),* 8.
KRISHNARATNE, S., LITTLECOTT, H., SELL, K., BURNS, J., RABE, J. E., STRATIL, J. M., LITWIN, T., KREUTZ, C., COENEN, M., GEFFERT, K., BOGER, A. H., MOVSISYAN, A., KRATZER, S., KLINGER, C., WABNITZ, K., STRAHWALD, B., VERBOOM, B., REHFUESS, E., BIALLAS, R. L., JUNG-SIEVERS, C., VOSS, S. & PFADENHAUER, L. M. 2022a. Measures implemented in the school setting to contain the COVID-19 pandemic. *Cochrane Database Syst Rev,* 1**,** Cd015029.
KRISHNARATNE, S., LITTLECOTT, H., SELL, K., BURNS, J., RABE, J. E., STRATIL, J. M., LITWIN, T., KREUTZ, C., COENEN, M., GEFFERT, K., BOGER, A. H., MOVSISYAN, A., KRATZER, S., KLINGER, C., WABNITZ, K., STRAHWALD, B., VERBOOM, B., REHFUESS, E., BIALLAS, R. L., JUNG-SIEVERS, C., VOSS, S. & PFADENHAUER, L. M. 2022b. Measures implemented in the school setting to contain the COVID-19 pandemic: a rapid review. *The Cochrane database of systematic reviews,* 1**,** CD015029.

CONFIDENTIAL

KUNSTLER, B., NEWTON, S., HILL, H., FERGUSON, J., HORE, P., MITCHELL, B. G., DEMPSEY, K., STEWARDSON, A. J., FRIEDMAN, D., COLE, K., SIM, M. R., FERGUSON, B., BURNS, P., KING, N., MCGLOUGHLIN, S., DICKS, M., MCCARTHY, S., TAM, B., HAZELTON, B., MCGURGAN, C., MCDONALD, S. & TURNER, T. 2022. P2/N95 respirators & surgical masks to prevent SARS-CoV-2 infection: Effectiveness & adverse effects. *Infection, disease & health*.

LAUSI, G., PIZZO, A., CRICENTI, C., BALDI, M., DESIDERIO, R., GIANNINI, A. M. & MARI, E. 2021. Intimate Partner Violence during the COVID-19 Pandemic: A Review of the Phenomenon from Victims' and Help Professionals' Perspectives. *International journal of environmental research and public health,* 18.

MBWOGGE, M. 2021. Mass Testing With Contact Tracing Compared to Test and Trace for the Effective Suppression of COVID-19 in the United Kingdom: Systematic Review. *JMIRx med,* 2**,** e27254.

MENDEZ-BRITO, A., BCHERAOUI, C. E. & POZO-MARTIN, F. 2021. Systematic review of empirical studies comparing the effectiveness of non-pharmaceutical interventions against COVID-19. *The Journal of infection*.

MIGNOGNA, C., COSTANZO, S., GHULAM, A., CERLETTI, C., DONATI, M. B., DE GAETANO, G., IACOVIELLO, L. & BONACCIO, M. 2021. Impact of Nationwide Lockdowns Resulting from The First Wave of the COVID-19 Pandemic on Food Intake, Eating Behaviours and Diet Quality: A Systematic Review. *Advances in nutrition (Bethesda, Md.)*.

MINOZZI, S., SAULLE, R., AMATO, L. & DAVOLI, M. 2021. [Impact of social distancing for covid-19 on the psychological well-being of youths: a systematic review of the literature.]. *Recenti progressi in medicina,* 112**,** 360-370.

NEIRA, C., MARDONES, R., NEIRA, C., GODINHO, R., RINCON, F., PEDROSO, J. & PEDROSO, J. 2021. Consequences of the covid-19 syndemic for nutritional health: A systematic review. *Nutrients,* 13.

NUSSBAUMER-STREIT, B., MAYR, V., DOBRESCU, A. I., CHAPMAN, A., PERSAD, E., KLERINGS, I., WAGNER, G., ZACHARIAH, C., GARTLEHNER, G., SIEBERT, U., LEDINGER, D., SIEBERT, U., SIEBERT, U., SIEBERT, U. & GARTLEHNER, G. 2020. Quarantine alone or in combination with other public health measures to control COVID-19: a rapid review. *Cochrane Database of Systematic Reviews,* 2020**,** CD013574.

OLIVEIRA CARVALHO, P., HÜLSDÜNKER, T. & CARSON, F. 2021. The Impact of the COVID-19 Lockdown on European Students' Negative Emotional Symptoms: A Systematic Review and Meta-Analysis. *Behavioral sciences (Basel, Switzerland),* 12.

PANCHAL, U., SALAZAR DE PABLO, G., FRANCO, M., MORENO, C., PARELLADA, M., ARANGO, C. & FUSAR-POLI, P. 2021. The impact of COVID-19 lockdown on child and adolescent mental health: systematic review. *European child & adolescent psychiatry*.

PANDA, P. K., GUPTA, J., CHOWDHURY, S. R., KUMAR, R., MEENA, A. K., MADAAN, P., SHARAWAT, I. K. & GULATI, S. 2020. Psychological and Behavioral Impact of Lockdown and Quarantine Measures for COVID-19 Pandemic on Children, Adolescents and Caregivers: A Systematic Review and Meta-Analysis. *Journal of tropical pediatrics,* 67.

PIZARRO, A. B., PERSAD, E., DURAO, S., NUSSBAUMER-STREIT, B., ENGELA-VOLKER, J. S., MCELVENNY, D., RHODES, S., STOCKING, K., FLETCHER, T., MARTIN, C., NOERTJOJO, K., SAMPSON, O., VERBEEK, J. H., JØRGENSEN, K. J. & BRUSCHETTINI, M. 2022. Workplace interventions to reduce the risk of SARS-CoV-2 infection outside of healthcare settings. *The Cochrane database of systematic reviews,* 5**,** CD015112.

QATHRIN, N., SARYONO, S. & MEKAR DWI, A. 2021. The Impact of Centralized Quarantine on Mental Health of People Affected By Covid-19: A Systematic Review. 6.

RAJKUMAR, E., RAJAN, A. M., DANIEL, M., LAKSHMI, R., JOHN, R., GEORGE, A. J., ABRAHAM, J. & VARGHESE, J. 2022. The psychological impact of quarantine due to COVID-19: A systematic review of risk, protective factors and interventions using socio-ecological model framework. *Heliyon,* 8**,** e09765.

CONFIDENTIAL

RAJMIL, L., HJERN, A., BORAN, P., GUNNLAUGSSON, G., KRAUS DE CAMARGO, O., RAMAN, S. & RAMAN, S. 2021. Impact of lockdown and school closure on children's health and well-being during the first wave of COVID-19: A narrative review. *BMJ Paediatr. Open,* 5**,** e001043.
REGMI, K., REGMI, K. & LWIN, C. M. 2021. Factors associated with the implementation of non-pharmaceutical interventions for reducing coronavirus disease 2019 (Covid-19): A systematic review. *Int. J. Environ. Res. Public Health,* 18.
REZWANUL HASAN, R., SYED AFROZ, K., JEFF, G., JEFF, G. & JEFF, G. 2021. A Systematic Literature Review of the Impact of COVID-19 Lockdowns on Air Quality in China. *Aerosol and Air Quality Research,* 21.
RIVERA, P. A., NYS, B. L. & FIESTAS, F. 2021. Impact of COVID-19 induced lockdown on physical activity and sedentary behavior among university students: A systematic review. *Medwave,* 21**,** e8456.
RODRIGUEZ-FERNANDEZ, P., GONZALEZ-SANTOS, J., SANTAMARIA-PELAEZ, M., SOTO-CAMARA, R., GONZALEZ-BERNAL, J. J. & SANCHEZ-GONZALEZ, E. 2021. Psychological effects of home confinement and social distancing derived from covid-19 in the general population—a systematic review. *Int. J. Environ. Res. Public Health,* 18.
RUNACRES, A., MACKINTOSH, K. A., KNIGHT, R. L., SHEERAN, L., THATCHER, R., SHELLEY, J. & MCNARRY, M. A. 2021. Impact of the COVID-19 Pandemic on Sedentary Time and Behaviour in Children and Adults: A Systematic Review and Meta-Analysis. *International journal of environmental research and public health,* 18.
SAMJI, H., WU, J., LADAK, A., VOSSEN, C., STEWART, E., DOVE, N., LONG, D. & SNELL, G. 2021. Review: Mental health impacts of the COVID-19 pandemic on children and youth - a systematic review. *Child and adolescent mental health*.
SANTESSO, N., GLENTON, C., DAHM, P., GARNER, P., AKL, E. A., ALPER, B., BRIGNARDELLO-PETERSEN, R., CARRASCO-LABRA, A., DE BEER, H. & HULTCRANTZ, M. 2020. GRADE guidelines 26: informative statements to communicate the findings of systematic reviews of interventions. *Journal of clinical epidemiology,* 119**,** 126-135.
SAULLE, R., MINOZZI, S., AMATO, L. & DAVOLI, M. 2021. [Impact of social distancing for covid-19 on youths' physical health: a systematic review of the literature.]. *Recenti progressi in medicina,* 112**,** 347-359.
SCHMIDT, R. A., GENOIS, R., JIN, J., VIGO, D., REHM, J. & RUSH, B. 2021. The early impact of COVID-19 on the incidence, prevalence, and severity of alcohol use and other drugs: A systematic review. *Drug and alcohol dependence,* 228**,** 109065.
SHEKARAIAH, S. & SURESH, K. 2021. Effect of Face Mask on Voice Production During COVID-19 Pandemic: A Systematic Review. *Journal of voice : official journal of the Voice Foundation*.
SIDELI, L., LO COCO, G., BONFANTI, R. C., BORSARINI, B., FORTUNATO, L., SECHI, C. & MICALI, N. 2021. Effects of COVID-19 lockdown on eating disorders and obesity: A systematic review and meta-analysis. *European eating disorders review : the journal of the Eating Disorders Association*.
SOHI, I., CHRYSTOJA, B. R., REHM, J., WELLS, S., MONTEIRO, M., ALI, S. & SHIELD, K. D. 2022. Changes in alcohol use during the COVID-19 pandemic and previous pandemics: A systematic review. *Alcoholism, clinical and experimental research,* 46**,** 498-513.
STEPHANIE, S., MIKE, T., MARK, T., JAE, S., YVONNE, B., LAURIE, B., DARAGH, M., FELIPE, S. & LEE, S. 2021. Changes in physical activity and sedentary behaviours from before to during the COVID-19 pandemic lockdown: a systematic review. *BMJ open sport and exercise medicine,* 7**,** e000960.
STRATIL, J. M., BIALLAS, R. L., BURNS, J., ARNOLD, L., GEFFERT, K., KUNZLER, A. M., MONSEF, I., STADELMAIER, J., WABNITZ, K., LITWIN, T., KREUTZ, C., BOGER, A. H., LINDNER, S., VERBOOM, B., VOSS, S. & MOVSISYAN, A. 2021. Non-pharmacological measures implemented in the setting of long-term care facilities to prevent SARS-CoV-2 infections and

CONFIDENTIAL

their consequences: a rapid review. *The Cochrane database of systematic reviews,* 9**,** CD015085.

SUÁREZ-GONZÁLEZ, A., RAJAGOPALAN, J., LIVINGSTON, G. & ALLADI, S. 2021. The effect of COVID-19 isolation measures on the cognition and mental health of people living with dementia: A rapid systematic review of one year of quantitative evidence. *EClinicalMedicine,* 39**,** 101047.

TABATABAEIZADEH, S. A. 2021. Airborne transmission of COVID-19 and the role of face mask to prevent it: a systematic review and meta-analysis. *European journal of medical research,* 26**,** 1.

TALIC, S., SHAH, S., WILD, H., GASEVIC, D., MAHARAJ, A., ADEMI, Z., LI, X., XU, W., MESA-EGUIAGARAY, I., ROSTRON, J., THEODORATOU, E., ZHANG, X., MOTEE, A., LIEW, D. & ILIC, D. 2021. Effectiveness of public health measures in reducing the incidence of covid-19, SARS-CoV-2 transmission, and covid-19 mortality: systematic review and meta-analysis. *BMJ (Clinical research ed.),* 375**,** e068302.

TULLY, M. A., MCMAW, L., ADLAKHA, D., BLAIR, N., MCANENEY, J., MCANENEY, H., CARMICHAEL, C., CUNNINGHAM, C., ARMSTRONG, N. C. & SMITH, L. 2021. The effect of different COVID-19 public health restrictions on mobility: A systematic review. *PloS one,* 16**,** e0260919.

VINER, R., RUSSELL, S., SAULLE, R., CROKER, H., STANSFIELD, C., PACKER, J., NICHOLLS, D., GODDINGS, A. L., BONELL, C., HUDSON, L., HOPE, S., WARD, J., SCHWALBE, N., MORGAN, A. & MINOZZI, S. 2022. School Closures During Social Lockdown and Mental Health, Health Behaviors, and Well-being Among Children and Adolescents During the First COVID-19 Wave: A Systematic Review. *JAMA pediatrics*.

VISWANATHAN, M., KAHWATI, L., HILL, C., JAHN, B., GIGER, K., DOBRESCU, A. I., KLERINGS, I., MEIXNER, J., PERSAD, E., TEUFER, B. & GARTLEHNER, G. 2020. Universal screening for SARS-CoV-2 infection: a rapid review. *Cochrane Database of Systematic Reviews,* 2020**,** CD013718.

WALL, S. & DEMPSEY, M. 2022. The effect of COVID-19 lockdowns on women's perinatal mental health: a systematic review. *Women and birth : journal of the Australian College of Midwives*.

WALSH, K. A., BRODERICK, N., AHERN, S., FAWSITT, C. G., O'BRIEN, K. M., CARRIGAN, M., HARRINGTON, P., O'NEILL, M., SMITH, S. M., SPILLANE, S., TELJEUR, C. & RYAN, M. 2022. Effectiveness of rapid antigen testing for screening of asymptomatic individuals to limit the transmission of SARS-CoV-2: A rapid review. *Reviews in medical virology***,** e2350.

WALSH, K. A., TYNER, B., BRODERICK, N., HARRINGTON, P., O'NEILL, M., FAWSITT, C. G., CARDWELL, K., SMITH, S. M., CONNOLLY, M. A. & RYAN, M. 2021a. Effectiveness of public health measures to prevent the transmission of SARS-CoV-2 at mass gatherings: A rapid review. *Reviews in medical virology***,** e2285.

WALSH, S., CHOWDHURY, A., BRAITHWAITE, V., RUSSELL, S., BIRCH, J. M., WARD, J. L., WADDINGTON, C., BRAYNE, C., BONELL, C., VINER, R. M. & MYTTON, O. T. 2021b. Do school closures and school reopenings affect community transmission of COVID-19? A systematic review of observational studies. *BMJ open,* 11**,** e053371.

YAACOUB, S., KHABSA, J., EL-KHOURY, R., EL-HARAKEH, A., LOTFI, T., SAAD, Z., ITANI, Z., KHAMIS, A. M., EL MIKATI, I., CUELLO-GARCIA, C. A., VERDUGO-PAIVA, F., RADA, G., SCHÜNEMANN, H. J., RIZK, N. & AKL, E. A. 2021. COVID-19 transmission during swimming-related activities: a rapid systematic review. *BMC infectious diseases,* 21**,** 1112.

YAGHOUBI, M., SALIMI, M. & MESKARPOUR-AMIRI, M. 2021. Systematic review of productivity loss among healthcare workers due to Covid-19. *The International journal of health planning and management*.

ZACCAGNI, L., TOSELLI, S. & BARBIERI, D. 2021. Physical Activity during COVID-19 Lockdown in Italy: A Systematic Review. *International journal of environmental research and public health,* 18.

CONFIDENTIAL

CONFIDENTIAL

APPENDICES

Appendix 1: Detailed search srategy

Appendix 2: Characteristics of each includd review

Appendix 3: AMSTAR scoring for each included review

Funding sources/sponsors

This review is funded by the NIPH

Conflicts of interest

None reported

CONFIDENTIAL

CONFIDENTIAL
